# Trends in burden of multidrug-resistant tuberculosis in countries, regions, and worldwide from 1990 to 2017: results from the Global Burden of Disease study

**DOI:** 10.1186/s40249-021-00803-w

**Published:** 2021-03-06

**Authors:** Ze-Jin Ou, Dan-Feng Yu, Yuan-Hao Liang, Wen-Qiao He, Yong-Zhi Li, Ya-Xian Meng, Hu-Sheng Xiong, Min-Yi Zhang, Huan He, Yu-Han Gao, Fei Wu, Qing Chen

**Affiliations:** 1 Guangdong Provincial Key Laboratory of Tropical Disease Research, Department of Epidemiology, School of Public Health, Southern Medical University, 1838 Guangzhou North Road, Guangzhou, 510515 China; 2 Department of MICU, Guangdong Women and Children Hospital, Guangzhou, China

**Keywords:** Multidrug-resistant tuberculosis, Global burden of disease, Age-standardized rate, Estimated annual percentage change, Epidemiological trend

## Abstract

**Background:**

Antituberculosis-drug resistance is an important public health issue, and its epidemiological patterns has dramatically changed in recent decades. This study aimed to estimate the trends of multidrug-resistant tuberculosis (MDR-TB), which can be used to inform health strategies.

**Methods:**

Data were collected from the Global Burden of Disease study 2017. The estimated annual percentage changes (EAPCs) were calculated to assess the trends of MDR-TB burden at global, regional, and national level from 1990 to 2017 using the linear regression model.

**Results:**

Globally, the age-standardized rate (ASR) of MDR-TB burden including incidence, prevalence, death and disability-adjusted life years (DALYs) had pronounced increasing trends from 1990 to 1999, with the EAPCs were 17.63 [95% confidence interval (*CI*): 10.77–24.92], 17.57 (95% *CI* 11.51–23.95), 21.21 (95% *CI* 15.96–26.69), and 21.90 (95% *CI* 16.55–27.50), respectively. Particularly, the largest increasing trends were seen in areas and countries with low and low-middle sociodemographic index (SDI). However, the trends in incidence, prevalence, death and DALYs of MDR-TB decreased globally from 2000 to 2017, with the respective EAPCs were − 1.37 (95% *CI* − 1.62 to − 1.12), − 1.32 (95% *CI* − 1.38 to − 1.26), − 3.30 (95% *CI* − 3.56 to − 3.04) and − 3.32 (95% *CI* − 3.59 to − 3.06). Decreasing trends of MDR-TB were observed in most regions and countries, particularly that of death and DALYs in Slovenia were − 18.96 (95% *CI* − 20.82 to − 17.06) and -19.35 (95% *CI* − 21.10 to − 17.55), respectively. Whereas the pronounced increasing trends of MDR-TB occurred in Papua New Guinea, Singapore, and Australia.

**Conclusions:**

The ASR of MDR-TB showed pronounced decreasing trends from 2000 to 2017. However, the MDR-TB burden remains a substantial challenge to the TB control globally, and requires effective control strategies and healthcare systems.
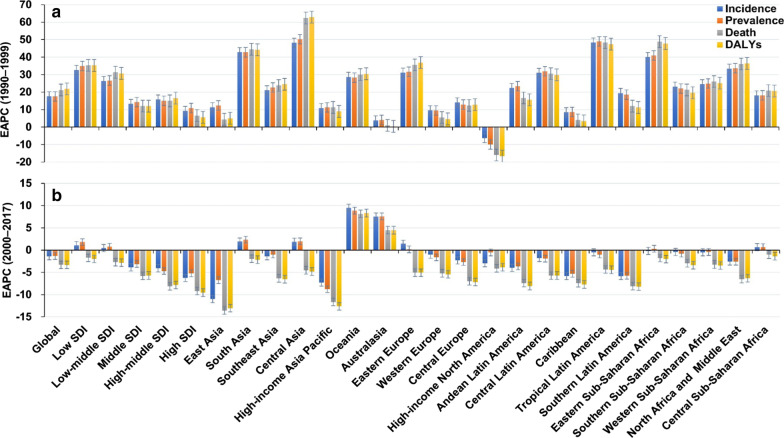

## Background

Resistance to anti-tuberculosis (TB) drugs has been an increasing challenge to global TB control in recent years [1, 2]. Multidrug-resistant tuberculosis (MDR-TB) is the major part of drug-resistant tuberculosis [3], and its trends are an important reference to the strategies of TB control.

MDR-TB is defined as the *Mycobacterium tuberculosis* that is resistant to isoniazid and rifampicin, which is strongly related to previous TB disease and its treatment [4, 5]. In the 1990s, MDR-TB outbreaks were reported in the USA and Europe [6, 7], and global surveillance of antituberculosis drug resistance was considered urgent and necessary. In 1994, a global program on surveillance of drug-resistant tuberculosis was launched by the World Health Organization (WHO), and the results showed that MDR-TB had increased dramatically worldwide [8, 9]. During 1994 and 1997, the global prevalence of MDR-TB was 1.4% in new TB patients, and 13% for TB patients treated previously [10]. It was reported that global MDR-TB occurred in an estimated 460,000 cases and resulted in 230,000 deaths in 2017, and accounted for 3.6% of all new cases and 17% of treated cases [11]. The highest burden of MDR-TB has been reported to be in China, India, Russia, and South Africa, and those countries have > 60% of all cases worldwide [12]. Meanwhile, the increasing MDR-TB has elicited problems with decreasing cure rates and survival times [13]. Furthermore, the MDR-TB prevalence has soaked up 47% of the expense for the response to anti-TB programs globally [14], and strained local health resources [8, 15]. These alarming data showed the urgent need for MDR-TB control.

The WHO set a “End TB” strategy based on a 90% decrease in the TB incidence documented in 2015 to be reached by 2035 [16]. More efficient strategies and increasing investment and commitment to MDR-TB control will be needed [17]. The Global Burden of Disease (GBD) study had provided a methodological and conceptual framework to quantify health loss, which facilitates the assessment of progress and challenges in the MDR-TB control. Here, we estimated trends in the MDR-TB burden from 1990 to 2017 using data derived from the GBD 2017, which would facilitate the improvement of strategies for TB control.

## Materials and methods

### Data source

The data of MDR-TB were acquired from the Global Burden of Disease (GBD) study 2017 with the Global Health Data Exchange query tool (http://ghdx.healthdata.org/gbd-results-tool). According to the GBD online tools instruction, the number and rate of MDR-TB incidence, prevalence, death, and disability-adjusted life years (DALYs) were extracted based on age, sex, sociodemographic index (SDI) area, geographic region, and country, without any inclusion/exclusion criteria. Age was stratified into five groups: < 5, 5–14, 15–49, 50–69, and > 70 years. The SDI areas were categorized into five levels: “low”, “low-middle”, “middle”, “high-middle”, and “high”. According to geographic factor, the world was divided into 21 geographic regions, and 195 countries/territories. The human development index (HDI) reflects the level of human development, and the availability of health resources in regions and countries. The data of HDI at national level were obtained from the United Nations Development Program (http://hdr.undp.org/en/data).

### Statistical analyses

The age-standardized rate (ASR) is a critical and representative parameter when considering differences in the age structure of multiple populations. The ASR is a weighted mean of age-specific rates. The ASR is calculated on basis of the age structure of standard populations using the following formula:

$${\text{ASR }} = \frac{{\sum_{i = 1}^{A} a_{i} w_{i} }}{{\sum_{i = 1}^{A} w_{i} }} \times 100,000$$

where *a*_*i*_ is the age-specific rate in the *i*^th^ age group, *w*_*i*_ is the number of persons (or the weight) in the corresponding *i*^th^ age subgroup of the selected reference standard population, and A is the number of age groups.

The ASR trend is a widely accepted measure that estimates the changing patterns of disease burden. The estimated annual percentage change (EAPC) is a common index reflecting the temporal trend of ASR, which has been described in detail elsewhere [18]. A regression line was fitted to the natural logarithm of the ASR. EAPC and its 95% confidence interval (*CI*) were estimated with the linear regression model. The formula is as following:

$${\text{y}} = \alpha + \beta {\text{x}} + \varepsilon$$

$${\text{EAPC}} = {1}00 \times \left( {{\text{exp}}\left( \beta \right) - {1}} \right)$$

where y = ln (ASR), and x is the calendar year. The trends were judged: if the EAPC and its 95% *CI* were > 0, the ASR was in an increasing trend; if EAPC and its 95% *CI* were < 0, the ASR was in a decreasing trend; other values meant that the ASR was stable over time. For the aim to analyze the impact factors of EAPC, the respective associations between EAPCs and ASR in 2000, and between EAPCs and HDI in 2017, were calculated by using the Pearson correlation analysis in the Statistical Package for Social Sciences (SPSS; version 25.0; SPSS Inc., Chicago, IL, USA). The choropleth maps were drawn using the statistical software R 3.6.2 (Lucent Technologies, Jasmine Mountain, USA). A *P* value of less than 0.05 was deemed to be statistically significant.

## Results

### Trends in MDR-TB incidence

**Fig. 1:**
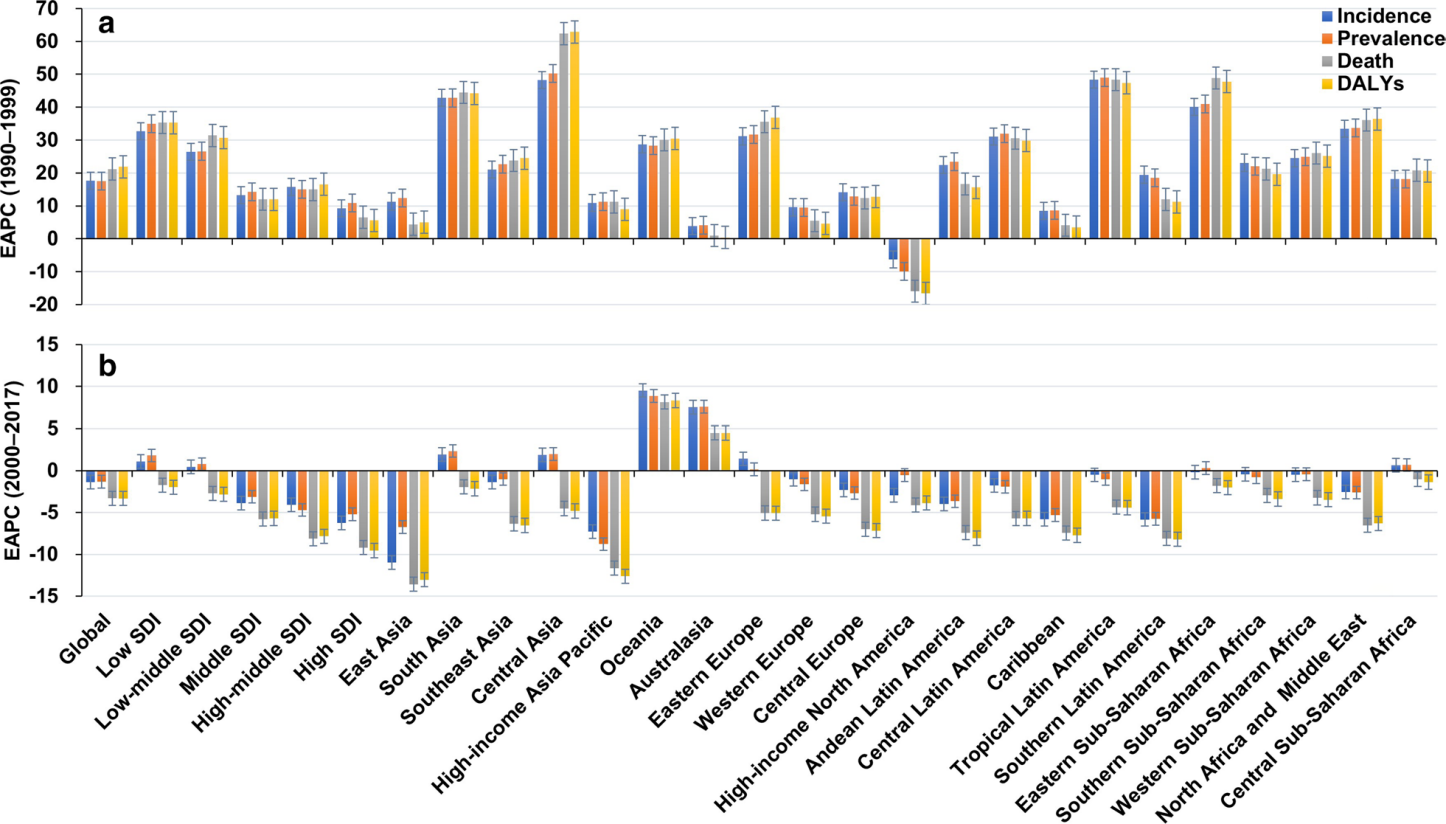
The trends of MDR-TB burden including incidence, prevalence, death, and DALYs globally, and in SDI areas and geographic regions. **a** The EAPCs of MDR-TB burden from 1990 to 1999; **b** The EAPCs of MDR-TB burden from 2000 to 2017. MDR-TB: multidrug resistant tuberculosis; DALYs: disability-adjusted life-years; EAPC: estimated annual percentage change; SDI: socio-demographic index.

During 1990 and 1999, the incidence of MDR-TB had a pronounced rising trend globally, with the overall age-standardized incidence rate (ASIR) increasing by an annual average of 17.63% (EAPC = 17.63, 95% *CI* 10.77–24.92). The upward trends of ASIR occurred in all SDI areas and geographic regions, expect high-income North America (Table 1; Fig. 1a). At national level, the ASIR of MDR-TB showed increasing trends in 186 countries/territories, particularly Turkmenistan, Somalia, and Kyrgyzstan, with the EAPCs were 66.78 (95% *CI* 55.50–78.89), 66.08 (95% *CI* 55.93–76.89), and 63.94 (95% *CI* 51.99–76.84), respectively. However, the downward trends of ASIR were observed only in United States (EAPC =  − 6.78, 95% *CI* − 8.19 to − 5.36) (Additional file 1: Table S4; Figure S7A-C).

**Table 1:** the percentage changes in absolute number and EAPCs of MDR-TB incidence from 1990 to 2017 in global, sexes, SDI areas and geographic regions

	1999		1990–1999		2017		2000–2017	
Characteristics	Number × 10^3^ (95% *UI*)	ASR/100 000 (95% *UI*)	Change in number (%)	EAPC (95% *CI*)	Number × 10^3^ (95% *UI*)	ASR/100 000 (95% *UI*)	Change in number (%)	EAPC (95% *CI*)
Overall	383.50 (290.12–517.25)	6.41 (4.85–8.64)	558.06	17.63 (10.77–24.92)	432.77 (254.61–726.95)	5.55 (3.29–9.29)	8.29	− 1.37 (- 1.62 to − 1.12)
Sex								
Male	215.27 (165.10–285.45)	7.36 (5.63–9.77)	575.03	17.89 (11.09–25.10)	240.92 (145.81–396.46)	6.17 (3.75–10.14)	7.13	− 1.58 (− 1.81 to − 1.34)
Female	168.23 (124.10–232.42)	5.55 (4.10–7.66)	537.55	17.30 (10.36–24.67)	191.85 (109.57–334.02)	4.99 (2.86–8.66)	9.80	− 1.13 (− 1.40 to − 0.86)
SDI								
Low	57.85 (31.12–110.89)	8.17 (4.30–16.14)	2054.73	32.73 (24.72–41.25)	121.88 (60.13–247.13)	11.23 (5.28–23.28)	90.22	1.08 (0.85–1.30)
Low-middle	82.58 (47.27–145.63)	7.59 (4.25–13.67)	1243.54	26.40 (18.82–34.46)	145.89 (70.89–263.95)	9.32 (4.44–17.28)	61.69	0.47 (0.11–0.84)
Middle	135.47 (87.38–194.95)	8.14 (5.26–11.8)	366.99	13.27 (6.4–20.59)	96.80 (48.14–175.83)	4.44 (2.22–8.10)	− 28.73	− 3.90 (− 4.17 to − 3.63)
High-middle	98.64 (66.20–143.87)	7.90 (5.26–11.56)	463.10	15.79 (8.51–23.55)	64.68 (42.19–96.12)	4.12 (2.70–6.14)	− 35.78	− 4.08 (- 4.63 to − 3.54)
High	6.05 (3.90–10.40)	0.54 (0.34–0.94)	183.49	9.29 (4.32–14.50)	2.86 (1.75–5.15)	0.22 (0.14–0.38)	− 52.36	− 6.25 (- 6.93 to − 5.57)
Regions								
East Asia	174.58 (118.01–246.69)	13.08 (8.89–18.48)	272.72	11.31 (4.02–19.11)	35.78 (8.19–111.13)	2.22 (0.51–6.96)	− 78.58	− 10.98 (− 11.66 to − 10.29)
South Asia	88.80 (32.12–200.49)	7.82 (2.84–17.74)	4328.87	42.85 (32.57–53.93)	216.16 (59.52–491.95)	12.96 (3.54–29.61)	112.02	1.91 (1.34–2.49)
Southeast Asia	18.74 (9.13–47.79)	4.01 (1.98–10.04)	891.37	21.07 (11.74–31.17)	20.14 (11.97–32.22)	3.11 (1.85–4.94)	4.47	− 1.36 (− 1.60 to − 1.13)
Central Asia	5.85 (3.87–9.25)	8.23 (5.42–12.93)	6722.47	48.26 (33.64–64.48)	15.00 (11.24–19.68)	16.14 (12.07–21.23)	112.47	1.87 (− 0.35–4.14)
High-income Asia Pacific	1.54 (1.13–2.20)	0.71 (0.51–1.03)	254.03	10.85 (5.12–16.88)	0.77 (0.18–2.12)	0.29 (0.07–0.82)	− 50.42	− 7.30 (− 8.70 to − 5.87)
Oceania	0.06 (0.01–0.20)	0.88 (0.18–2.77)	1528.54	28.75 (21.89–36)	0.57 (0.29–1.00)	4.80 (2.44–8.35)	710.75	9.53 (7.93–11.15)
Australasia	0.01 (0.005–0.02)	0.05 (0.02–0.10)	61.42	3.86 (1.31–6.47)	0.04 (0.03–0.07)	0.15 (0.09–0.24)	323.96	7.54 (7.09–8.00)
Eastern Europe	30.93 (22.15–42.10)	12.70 (9.12–17.28)	1667.60	31.15 (21.59–41.46)	41.35 (27.21–57.89)	17.64 (11.54–24.59)	21.56	1.41 (0.16–2.66)
Western Europe	0.70 (0.59–0.81)	0.18 (0.15–0.21)	144.69	9.58 (8.21–10.97)	0.73 (0.57–0.95)	0.18 (0.14–0.23)	0.56	− 1.01 (− 1.51 to − 0.51)
Central Europe	0.79 (0.53–1.30)	0.60 (0.39–0.97)	354.42	14.17 (6.6–22.27)	0.58 (0.39–0.82)	0.45 (0.29–0.63)	− 27.56	− 2.29 (− 3.28 to − 1.29)
High-income North America	0.23 (0.19–0.28)	0.07 (0.05–0.08)	− 38.50	-6.28 (-7.68 to − 4.86)	0.12 (0.08–0.18)	0.03 (0.02–0.05)	− 41.23	-2.96 (− 3.33 to − 2.59)
Andean Latin America	3.23 (1.59–5.84)	7.64 (3.76–13.79)	773.25	22.40 (18.12–26.83)	2.81 (2.05–4.19)	4.62 (3.36–6.9)	− 18.48	− 3.98 (− 4.41 to − 3.54)
Central Latin America	1.02 (0.59–1.81)	0.62 (0.35–1.08)	2211.58	31.07 (20.52–42.54)	1.42 (0.66–2.92)	0.56 (0.26–1.15)	28.99	− 1.76 (− 2.35 to − 1.16)
Caribbean	0.17 (0.07–0.48)	0.44 (0.17–1.22)	167.35	8.51 (4.22–12.98)	0.10 (0.04–0.23)	0.20 (0.08–0.49)	− 44.12	− 5.79 (− 7.83 to − 3.71)
Tropical Latin America	1.40 (0.38–3.57)	0.84 (0.23–2.11)	5272.18	48.39 (39.68–57.64)	2.12 (0.48–5.78)	0.90 (0.21–2.46)	37.90	− 0.51 (− 1.42–0.41)
Southern Latin America	0.17 (0.10–0.30)	0.32 (0.18–0.54)	511.06	19.46 (16.21–22.79)	0.11 (0.03–0.32)	0.16 (0.05–0.49)	− 40.47	− 5.83 (− 6.87 to − 4.79)
Eastern Sub-Saharan Africa	17.55 (10.87–28.72)	8.75 (5.23–14.98)	3920.28	40.07 (29.48–51.53)	34.27 (21.73–53.29)	9.62 (6.20–14.99)	78.79	− 0.17 (− 0.70–0.35)
Southern Sub-Saharan Africa	8.03 (4.20–17.60)	12.38 (6.47–27.75)	707.05	23.11 (22.54–23.67)	11.15 (6.21–20.66)	13.79 (7.65–25.24)	20.43	− 0.46 (− 1.86–0.96)
Western Sub-Saharan Africa	17.21 (7.77–38.43)	8.32 (3.76–18.33)	1164.55	24.50 (17.01–32.47)	30.44 (13.54–64.69)	8.59 (3.83–18.26)	62.28	− 0.48 (− 1.02–0.06)
North Africa and Middle East	6.76 (4.11–10.36)	1.74 (1.06–2.67)	1760.45	33.46 (28.65–38.46)	7.10 (4.65–11.81)	1.21 (0.8–2.00)	− 2.84	− 2.56 (− 2.73 to − 2.39)
Central Sub-Saharan Africa	5.72 (1.73–16.90)	10.17 (3.12–29.55)	673.46	18.11 (10.74–25.97)	12.01 (2.87–32.58)	12.33 (2.97–34.12)	99.63	0.65 (0.45–0.85)

MDR-TB: Multidrug resistant tuberculosis; EAPC: Estimated annual percentage change; ASR: Age-standardized rate; *CI* Confidence interval; *UI*: uncertainty interval; SDI: Socio-demographic index

**Fig. 2:**
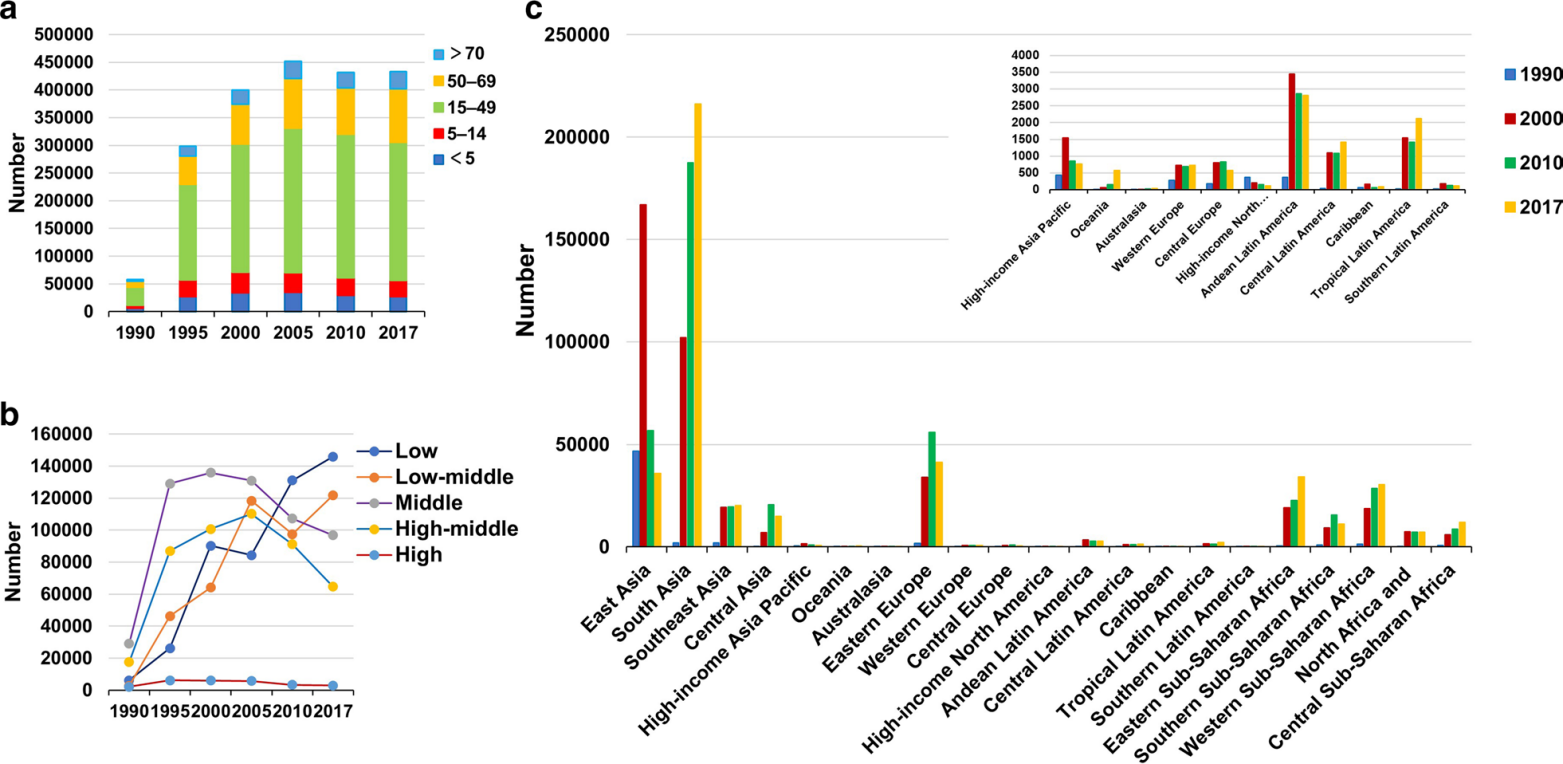
The distribution of the incident number of MDR-TB globally, and in SDI areas and geographic regions from1990 to 2017. **a** The incident number of MDR-TB in age groups; **b** The changing of incident number of MDR-TB in SDI areas; **c** The incident number of MDR-TB in geographical regions. MDR-TB: multidrug resistant tuberculosis; SDI: socio-demographic index

**Fig. 3:**
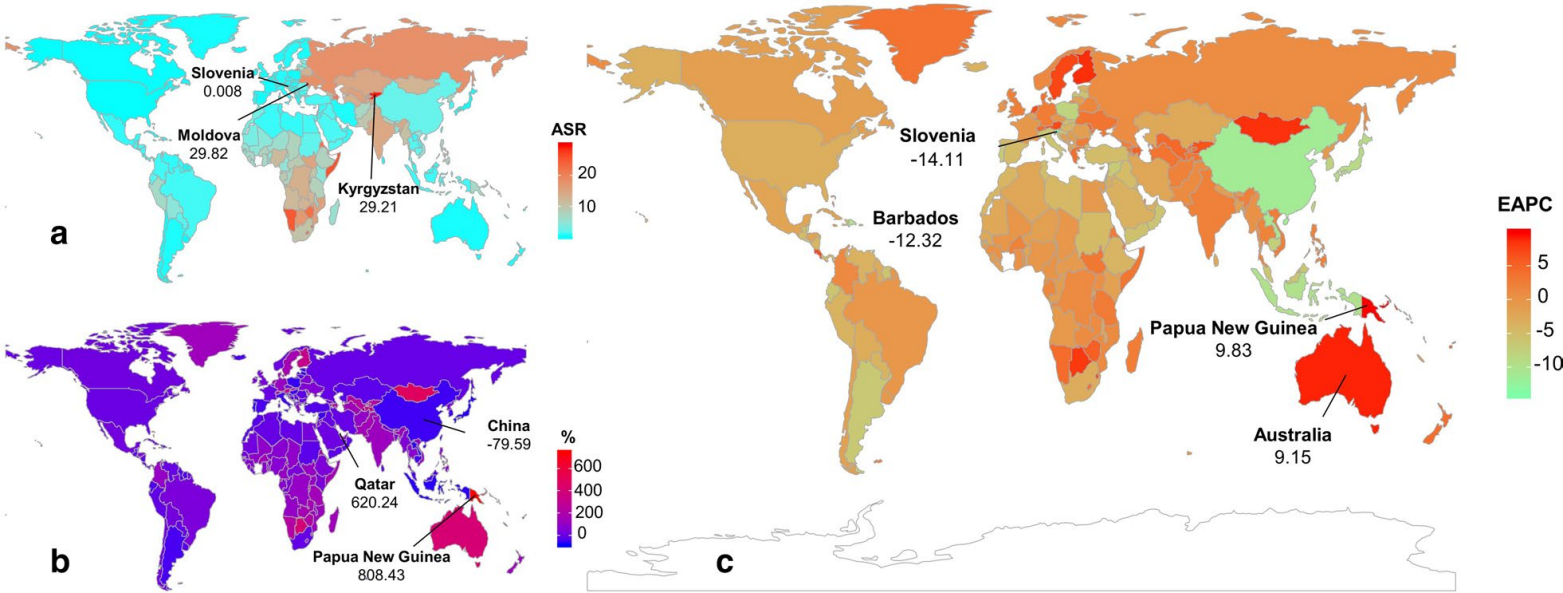
The distribution of ASR, percentage changes in absolute number, and EAPC of MDR-TB incidence at a national level during 2000–2017. **a** The ASR of MDR-TB incidence in 2017 in countries/territories; **b** The percentage changes in absolute incident number of MDR-TB between 2000 and 2017 in countries/territories; **c** The EAPCs of MDR-TB incidence in countries/territories from 2000 to 2017. Countries/territories with an extreme value were annotated. MDR-TB: multidrug resistant tuberculosis; ASR: Age-standardized rate; EAPC: Estimated annual percentage change

**Fig. 4:**
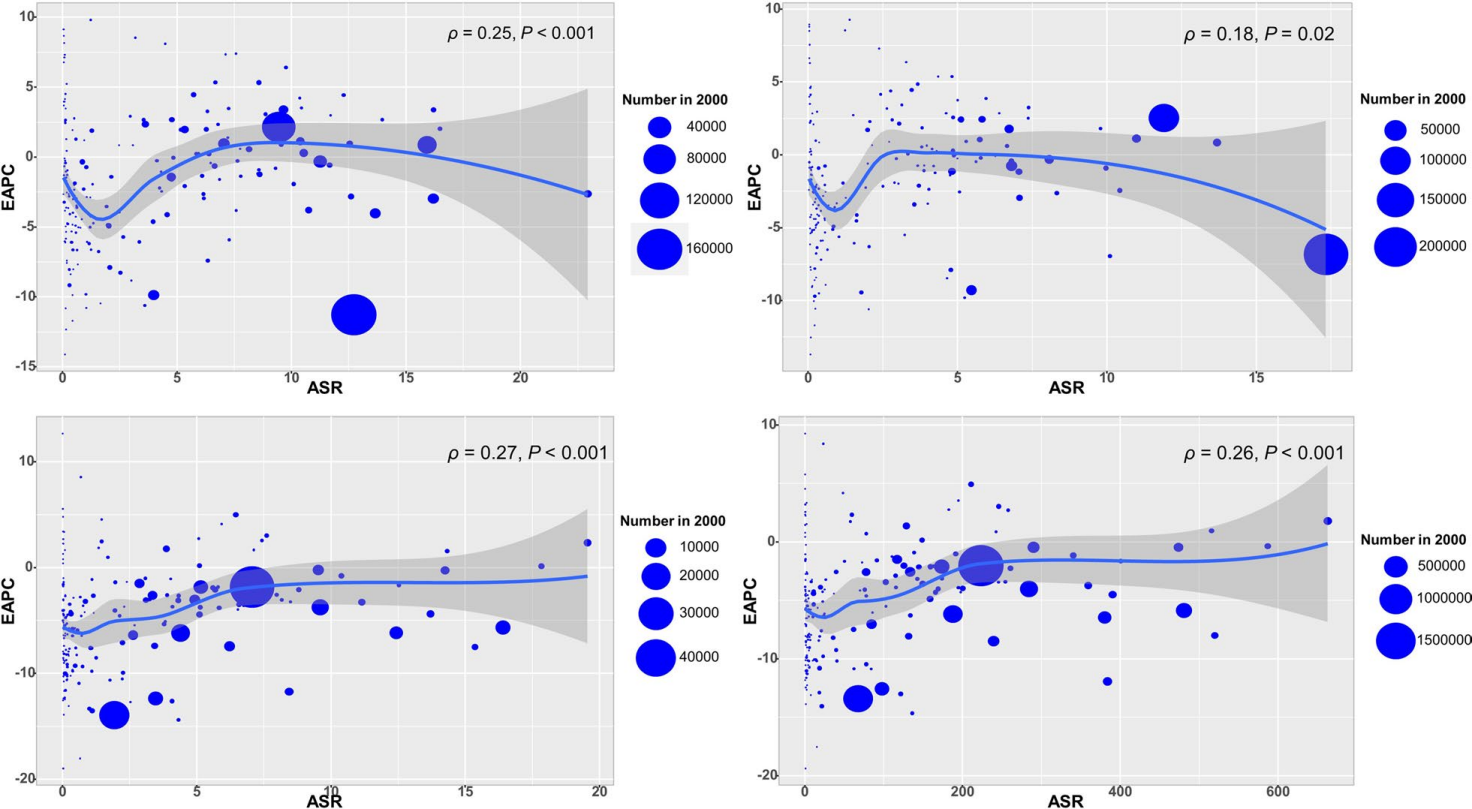
The correlation between EAPCs (2000–2017) and ASR in 2000 at a national level. The EAPCs of MDR-TB burden including incidence (**a**), prevalence (**b**), death (**c**), and DALYs (**d**) had a positive correlation with the ASR in 2000, respectively. The correlation was calculated with Pearson correlation analysis. The size of circle increases with the numbers in 2000 in the corresponding countries. MDR-TB: multidrug resistant tuberculosis; ASR: Age-standardized rate; EAPC: Estimated annual percentage change; HDI: Human development index; DALYs: disability-adjusted life-years.

Globally, the incident number of MDR-TB was 432.70 × 10^3^ (95% uncertainty interval (*UI*): 254.61 × 10^3^–726.95 × 10^3^) in 2017, with an increase of 8.29% from 2000 to 2017. During 2000 and 2017, the ASIR had a decreasing trend (EAPC =  − 1.37, 95% *CI* − 1.62 to − 1.12) (Table 1; Fig. 1b). The downward trends of ASIR occured in SDI areas, expect low and low-middle SDI areas. In terms of 21 regions, the most pronounced decreasing trends were observed in East Asia and High-income Asia Pacific, in which EPACs were –10.98(95% *CI* − 11.66 to − 10.29) and − 7.30 (95%*CI* − 8.70 to − 5.87). Whereas the largest increasing trends occurred in Oceania (EAPC = 9.53, 95% *CI* 7.93–11.15) (Table 1; Figs. 1b, 2a–c). Among 195 countries/territories, the ASIR showed downward trends in 112 countries, and the largest one was in Slovenia from 2000 to 2017 (EAPC =  − 14.11, 95% *CI* − 15.36 to − 12.83). On the other hand, the ASIR showed rising trends in 54 countries, particularly Papua New Guinea (EAPC = 9.83, 95% *CI* 8.23–11.45), followed by Australia and Finland (Additional file 1: Table S5; Fig. 3a–c). The EAPCs (2000–2017) had a positive correlation with the ASIR in 2000 at a national level (ρ = 0.25, *P* < 0.001) (Fig. 4a), but no with the HDI in 2017.

### Trends in MDR-TB prevalence

Globally, the ASR of prevalence showed an obvious upward trend from 1990 to 1999, with the EAPC was 17.57 (95% *CI* 11.51–23.95). In SDI areas and geographic regions, the ASR of prevalence had the similar trends to that of ASIR from 1990 to 1999 (Additional file 1: Table S1; Fig. 1a). With regard to national level, from 1990 to 1999, the rising trends were observed in 185 countries/territories, and the largest one was Somalia (EAPC = 67.92, 95% *CI* 58.28–78.16), followed by Turkmenistan, and Kyrgyzstan. However, the trends of prevalence decreased only in United States, with the EAPC of – 10.84 (95% *CI* − 11.40 to − 10.28) (Additional file 1: Table S4, Figure S8A-C ).

The number of MDR-TB prevalence was 464.12 × 10^3^ (95% *UI*: 229.12 × 10^3^–863.33 × 10^3^) worldwide in 2017, with an increase of 9.92% from 2000 to 2017. During 2000 and 2017, the prevalence of MDR-TB had a downward trend globally, with the EAPC was − 1.32 (95% *CI* − 1.38 to − 1.26) (Additional file 1: Table S1; Fig. 1). The trends of prevalence rose in areas of low- and low-middle SDI, while decreasing in other areas. Among 21 geographical regions, the most pronounced downward trends were observed in High-income Asia Pacific (EAPC =  − 8.77, 95% *CI* − 9.98 to − 7.55), while the largest increasing trends occurred in Oceania and Australasia (Additional file 1: Table S1; Fig. 1b, and Additional file 1: Figure S1A-C). At national level, the decreasing trends of prevalence were observed in 104 countries from 2000 to 2017, particularly Slovenia (EAPC =  − 13.69, 95% *CI* − 15.04 to − 12.31). While the rising trends occurred in 55 countries, and the largest one was in Papua New Guinea (EAPC = 9.28, 95% *CI* 7.81–10.78), followed by Finland and Australia (Additional file 1: Table S5; Figure S4A-C). The EAPCs (2000–2017) had a positive correlation with the ASR of prevalence in 2000 at a national level (ρ = 0.18, *P* = 0.02) (Fig. 4b), but no with the HDI in 2017.

### Trends in MDR-TB related death

Globally, the age-standardized death rate (ASDR) of MDR-TB had a rising trend from 1990 to 1999 (EAPC = 21.21, 95% *CI* 15.96–26.69). During 1990 and 1999, the upward trends of ASDR were observed in all SDI areas and most geographic regions, whereas the decreasing trend occurred only in High-income North America (EAPC =  − 15.91, 95% *CI* − 18.52 to − 13.22) (Additional file 1: Table S2; Fig. 1a). At national level, the upward trends were documented in 186 countries/territories, and the largest increasing occurred in Somalia (EAPC = 81.08, 95% *CI* 72.54–90.05), followed by Kyrgyzstan, and Turkmenistan. However, the decreasing trends were seen in six countries, particularly United States, in which the EAPC was − 16.72 (95% *CI* − 19.41 to − 13.94) (Additional file 1: Table S4, Figure S9A- C).

**Fig. 5:**
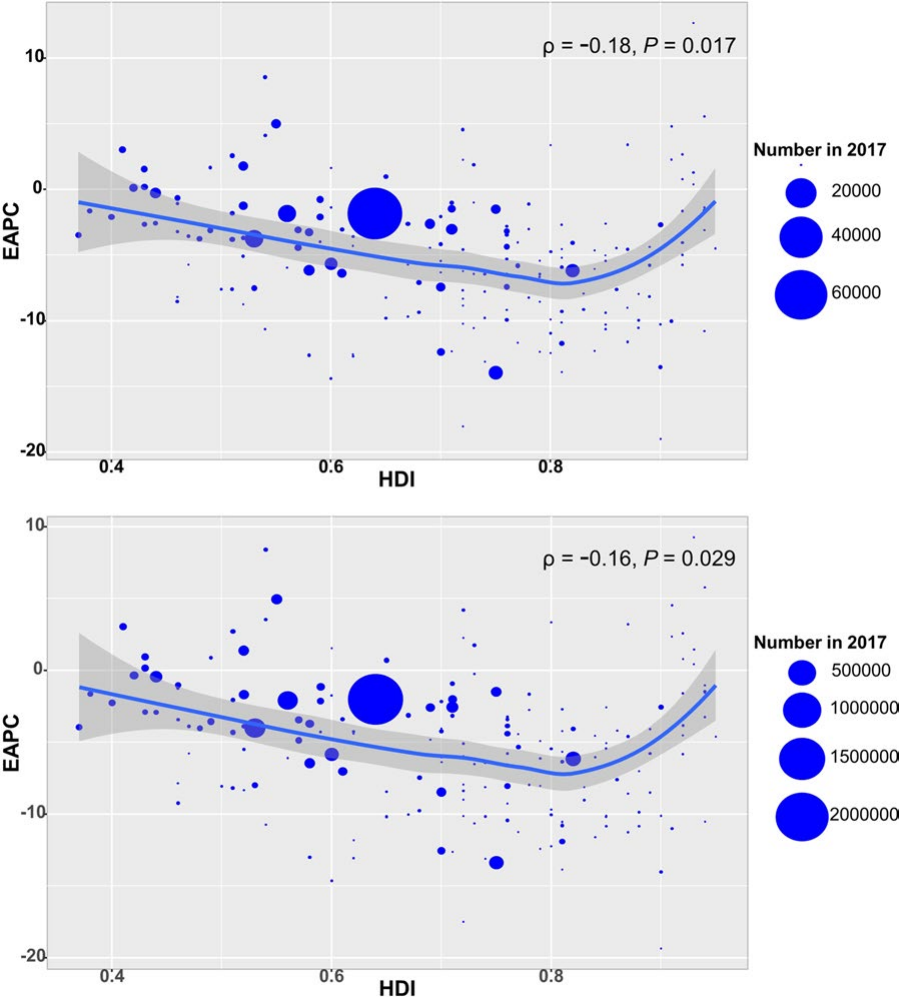
The correlation between EAPCs (2000–2017) and HDI in 2017 at a national level. **a** The EAPCs of death due to MDR-TB had a negative correlation with the HDI in 2017; **b** The EAPCs of DALYs due to MDR-TB had a negative correlation with the HDI in 2017. The correlation was calculated with Pearson correlation analysis. The circles represent countries that were available on HDI data, and the size of circle increases with the numbers in 2017 in the corresponding countries. MDR-TB: multidrug resistant tuberculosis; ASR: Age-standardized rate; EAPC: Estimated annual percentage change; HDI: Human development index

The number of deaths attributed to MDR-TB was 126.89 × 10^3^ (95% *UI*: 70.06 × 10^3^–202.17 × 10^3^) in 2017, with a decrease of 10.17% from 2000 to 2017. Globally, the ASDR of MDR-TB had a decreasing trend from 2000 to 2017 (EAPC =  − 3.30, 95% *CI* − 3.56 to − 3.04) (Additional file 1: Table S2; Fig. 1b). The ASDR had downward trends in all SDI areas and regions from 2000 to 2017, expect Oceania and Australasia. The most pronounced decreasing trends were documented in East Asia (EAPC =  − 13.57, 95% *CI* − 14.21 to − 12.92), followed by high-income Asia Pacific and Southern Latin America (Additional file 1: Table S2; Fig. 1b, and Additional file 1: Figure S2A-C). At national level, the decreasing trends were documented in 164 countries/territories from 2000 to 2017, and the largest one was in Slovenia (EAPC =  − 18.96, 95% *CI* − 20.82 to − 17.06), followed by Maldives, and Laos. On the other hand, the upward trends of ASDR were observed in seventeen countries, particularly Singapore (EAPC = 12.67, 95% *CI* 10.60–14.78) (Additional file 1: Table S5; Figure S5A-C). The EAPCs (2000–2017) had a positive correlation with the ASDR in 2000, and a negative correlation with the HDI in 2017 at a national level (ρ = 0.27, *P* < 0.001, Fig. 4c; ρ =  − 0.18, *P* = 0.017, Fig. 5a, respectively).

### Trends in MDR-TB-related DALYs

Pronounced increasing trend was observed in DALYs associated with MDR-TB from 1990 to 1999, with an EAPC was 21.90 (95% *CI* 16.55–27.50). The increasing trends occurred in all SDI areas and geographic regions. The largest rising trends of DALYs were observed in Central Asia (EAPC = 62.87, 95% *CI* 47.83–79.44), followed by Tropical Latin America, and South Asia (Additional file 1: Table S3; Fig. 1a). Rising trends of DALYs were observed in 163 countries/territories, with the most pronounced one being in Somalia (EAPC = 79.93, 95% *CI* 70.87–89.47), followed by Turkmenistan, and Kyrgyzstan. However, seven countries had decreasing trends, particularly United States (EAPC =  − 17.26, 95% *CI* − 19.91 to − 14.54) (Additional file 1: Table S4; Figure S10A-C).

Globally, the number of DALYs due to MDR-TB was 4647.99 × 10^3^ (95% *UI*: 2663.04 × 10^3^–7224.23 × 10^3^) in 2017, with a decrease of 17.71% from 2000 to 2107. The ASR of DALYs had a decreasing trend (EAPC =  − 3.32, 95% *CI* − 3.59 to − 3.06) (Additional file 1: Table S3; Fig. 1b). During 2000 and 2017, the downward trends of DALYs were observed in SDI areas and regions, expect Oceania and Australasia. The largest decreasing trends in DALYs were observed in East Asia and high-income Asia Pacific, in which the EAPCs were − 13.02 (95% *CI* − 13.58 to − 12.46) and − 12.62 (95% *CI* − 13.85 to − 11.38), respectively (Additional file 1: Table S2; Fig. 1b, and Additional file 1: Figure S3A-C). At national level, the downward trends occurred in 164 countries/territories, with the largest decreasing trend being in Slovenia, Maldives, and Laos, in which the EAPCs were − 19.35 (95% *CI* − 21.10 to − 17.55), − 17.50 (95% *CI* − 18.33 to − 16.66), and − 14.63 (95% *CI* − 15.65 to − 13.59), respectively. While upward trends were observed in nineteen countries/territories, with the largest one being in Singapore (EAPC = 9.28, 95% *CI* 7.78–10.79), followed by Papua New Guinea and Zimbabwe (Additional file 1: Table S5; Figure S6A-C). The EAPCs (2000–2017) had a positive correlation with the ASR of DALYs in 2000, and a negative correlation with the HDI in 2017 at a national level (ρ = 0.26, *P* < 0.001, Fig. 4d; ρ = –0.16, *P* = 0.029, Fig. 5b, respectively).

## Discussion

In present study, the authors observed that the ASR of MDR-TB presented a parabolic distribution during the period 1990–2017, which peaked around 1999, and the lowest values were in 1990 and in 2017, respectively. For example, the largest overall ASIR were 6.41 in 1999, and the lowest values were 1.12 in 1990 and 5.55 in 2017, respectively. Therefore, 1999 was selected as the time cut-off point to describe its trends in two time periods, including 1990–1999, and 2000–2017. The ASR of MDR-TB burden, including incidence, prevalence, death, and DALYs, dramatically increased globally between 1990 and 1999, but showed decreasing trends from 2000 to 2017, which objectively reflected the changing trends.

During 1990–1999, the absolute number of MDR-TB rapidly rose globally, and the ASR had the largest increasing trends in the areas of low and low-middle SDI areas. In these areas, there existed many stumbling blocks to TB control including population expansion, poverty, and overloaded health systems [19, 20]. These factors could also explain why the EAPCs were negatively associated with HDI. Among geographic regions, central Asia showed the largest increasing trends of MDR-TB from 1990 to 1999. TB patients living in former Soviet countries had a high risk of MDR-TB development [21], probably attributed to a transmissible branch of the *M. tuberculosis* Beijing genotype: central Asia outbreak clade [22]. Somalia, Turkmenistan, and Kyrgyzstan had the most pronounced increasing trends in the ASR of MDR-TB from 1990 to 1999. In these countries, poverty, malnutrition and poor health infrastructure are considerable barriers to health and wellbeing [23, 24]. Furthermore, the situation has been accelerated by an inundation of drug use and human immunodeficiency virus (HIV) infection [25, 26]. Whereas a decreasing trend of MDR-TB was observed in High-income North America, where the robust healthcare systems could treat TB effectively, and consequently to reduce the development of MDR-TB [27].

However, the decreasing trends of MDR-TB occurred in countries, regions and worldwide from 2000 to 2017, which was probably due to effective management and control of TB in recent years. The global tuberculosis report 2018 revealed that TB incidence was falling at about 2% per year, and the overall reduction of mortality rate during 2000–2017 was estimated to be 42% [28]. The testing, detection and treatment of MDR-TB had achieved apparent progress globally, for example, up to 41% of TB patients were tested for rifampicin resistance in 2017 [29], and recommendations on the treatment and care of drug-resistant tuberculosis has been compiled and issued by WHO from 2011 to 2019[30]. Meanwhile, management of MDR-TB contacts had been developed in recent years, and the preventive therapy (TPT) could reduce the risk to development of MDR-TB by up to 90% [31]. Particularly, governments and political organizations have taken action for DR-TB control through action plans, financial initiatives, and health infrastructure worldwide [32, 33]. Pronounced decreasing trends of MDR-TB were found in Slovenia, Laos, and China. Due to the effective TB prevention and therapeutic programs, the combined MDR-TB prevalence was 1% or less in Slovenia [34]. Meanwhile, the efficient managements of TB were implemented in Slovenia, and the completeness of TB notification was as much as 100% [35]. Benefiting from the United Nation's Sustainable Development Goals (SDGs), the universal health coverage index had been substantially improved in Laos during the period 2000–2016 [36], which facilitated the control of TB. China was one of the countries with the largest population of TB in the world. The Chinese government started to revitalize anti-TB programs in 1990s, and carried out forceful measures to achieve considerable successes in MDR-TB control [37], which could provide a good example to other developing counties. A meta-analysis confirmed that the prevalence of MDR-TB had a decreasing trend in China from 1996 to 2014 [38]. Simultaneously, the government carried out national initiatives, including reducing poverty, improving health infrastructure, and implementing a new medical-care system in rural areas [39]. Surprisingly, several countries with a low incidence of tuberculosis exhibited obvious increasing trends of MDR-TB from 2000 to 2017, including Singapore, Australia and Papua New Guinea. These countries hold the most frequent population migration all over the world. In Singapore, 80% of MDR-TB patients occurred among the foreign population during 2000–2010, with an increasing trend after 2004 [40]. Papua New Guinea has a high TB burden, and causes challenges to cross-border management of MDR-TB in the Torres Strait, Australia [41]. Therefore, the importance of pre-immigration screening to detect the infectious disease should be emphasized [42].

Our study had three main limitations. First, estimation of disease burden was dependent mainly on the quality and quantity of data, and the accuracy and robustness of GBD estimates may have been impaired by a potential bias, including unreported cases, incomplete testing and reporting, and the test technology of MDR-TB varied across countries and over time. However, the GBD tuberculosis collaborators applied various modelling methods to estimates for locations with sparse data. For example, in countries without vital registration data, the tuberculosis death was assessed using the verbal autopsy studies, which had proven to be credible and sensitive [43]. Second, because of the limitation of ASR estimates, the trend of MDR-TB in age groups only were presented using percentage changes in absolute numbers. Third, the risk factors and clinical information of MDR-TB were not available, so the reason for the changing trends could not be investigated further.

## Conclusions

The trends of MDR-TB burden dramatically increased during the period 1990–1999, but decreased from 2000 to 2017, indicating that the current control strategies are effective and feasible. However, the MDR-TB burden remains a substantial challenge to the public health globally, and more efficient strategies and increasing investment to the prevention and control of MDR-TB are required.

## Supplementary Information

**Additional file 1.** The supplementary figures and tables of trends in MDR-TB from 1990 to 2017.

## Data Availability

All data during this study are included in this published article and its additional files.

## Abbreviations

DALYs: Disability-adjusted life-years
GBD: Global burden of disease
SDGs: Sustainable development goals
EAPC: Estimated annual percentage change
ASR: Age-standardized rate
CI: Confidence interval
UI: Uncertainty interval
SDI: Socio-demographic index
HDI: Human development index
MDR-TB: Multidrug-resistant tuberculosis
TPT: The preventive therapy
