# Supplementary material for: Trends in burden of multidrug-resistant tuberculosis in countries, regions, and worldwide from 1990 to 2017: results from the Global Burden of Disease study
Source: Infect Dis Poverty. 2021 Mar 6;10:24. doi: 10.1186/s40249-021-00803-w (PMC7936417; doi:10.1186/s40249-021-00803-w)
Supplement: Supplementary file 1 — Additional file 1. The supplementary figures and tables of trends in MDR-TB from 1990 to 2017. [file 40249_2021_803_MOESM1_ESM.docx]

**Figure S1**. The distribution of the number of MDR-TB prevalence globally, and in SDI areas and geographic regions from1990 to 2017. (A) the number of MDR-TB prevalence in age groups; (B) the changing of number of MDR-TB prevalence in SDI areas; (C) the number of MDR-TB prevalence in geographical regions. Abbreviations: MDR-TB: multidrug resistant tuberculosis; SDI: socio-demographic index.

**
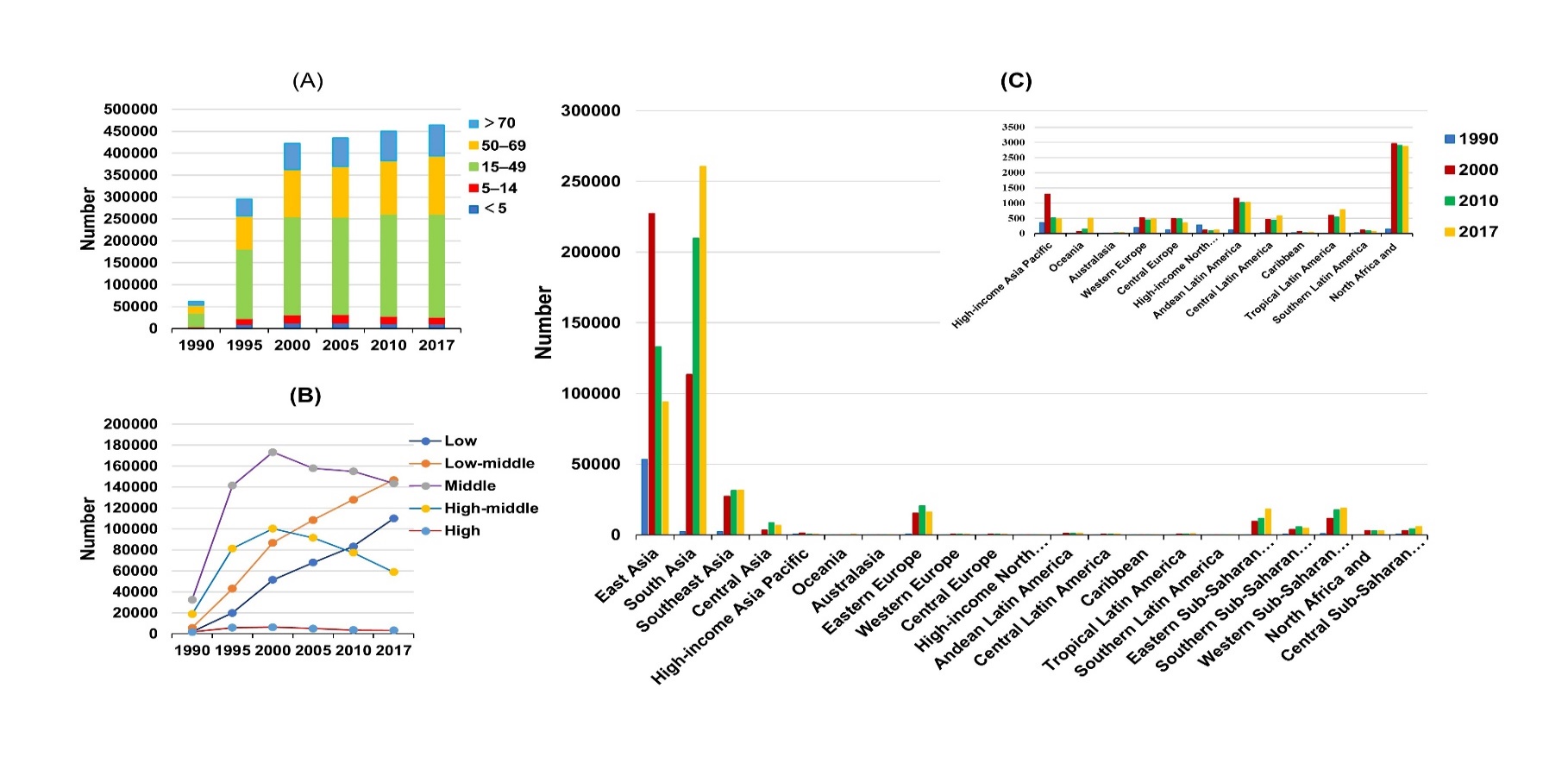
**

**Figure S2**. The distribution of the death number caused by MDR-TB globally, and in SDI areas and geographic regions from1990 to 2017. (A) the number of MDR-TB death in age groups; (B) the changing of number of MDR-TB death in SDI areas; (C) the number of MDR-TB death in geographical regions. Abbreviations: MDR-TB: multidrug resistant tuberculosis; SDI: socio-demographic index.


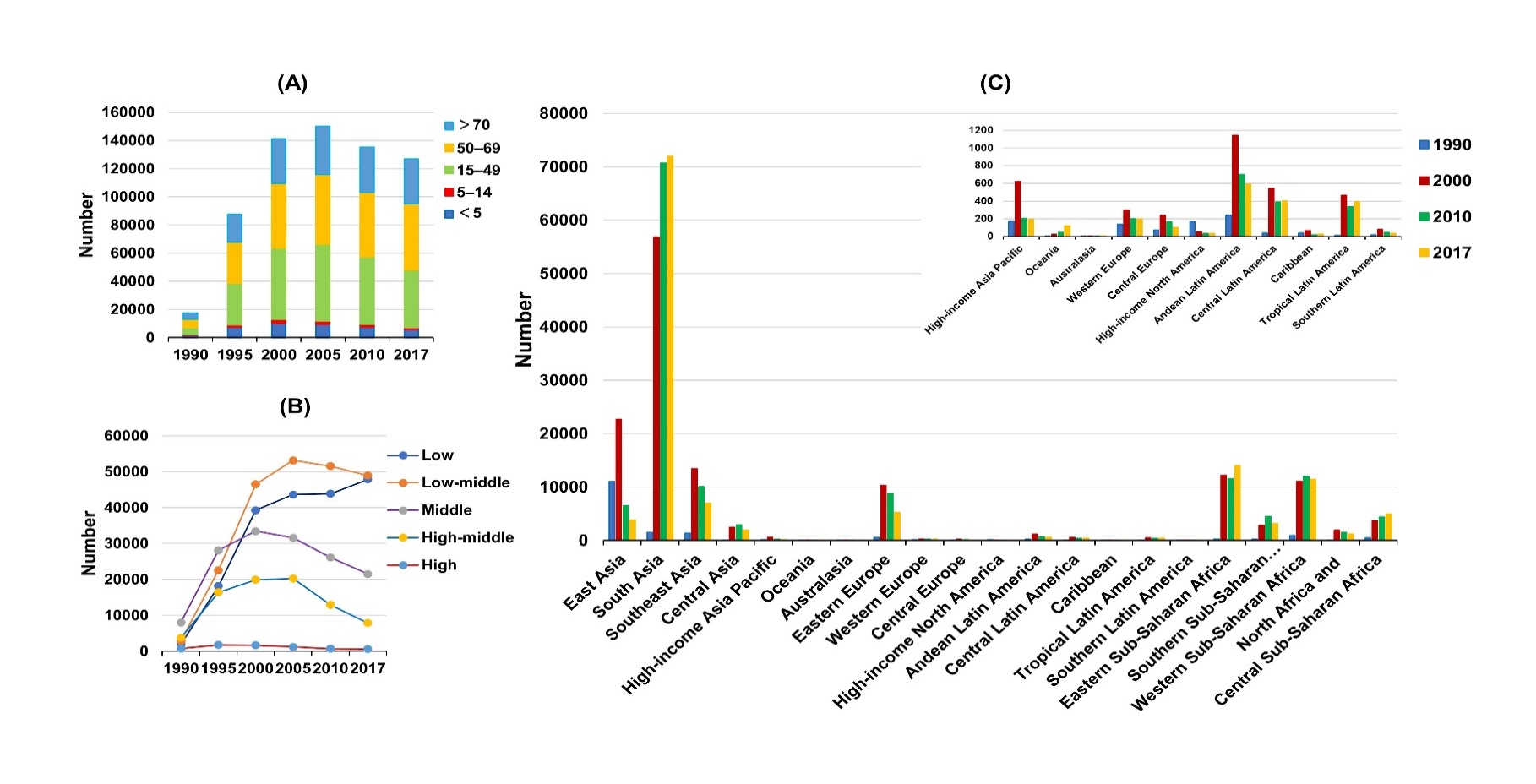


**Figure S3**. The distribution of the DALYs number caused by MDR-TB globally, and in SDI areas and geographic regions from1990 to 2017. (A) the number of MDR-TB DALYs in age groups; (B) the changing of number of MDR-TB DALYs in SDI areas; (C) the number of MDR-TB DALYs in geographical regions. Abbreviations: MDR-TB: multidrug resistant tuberculosis; SDI: socio-demographic index; DALYs: disability-adjusted life-years.

**
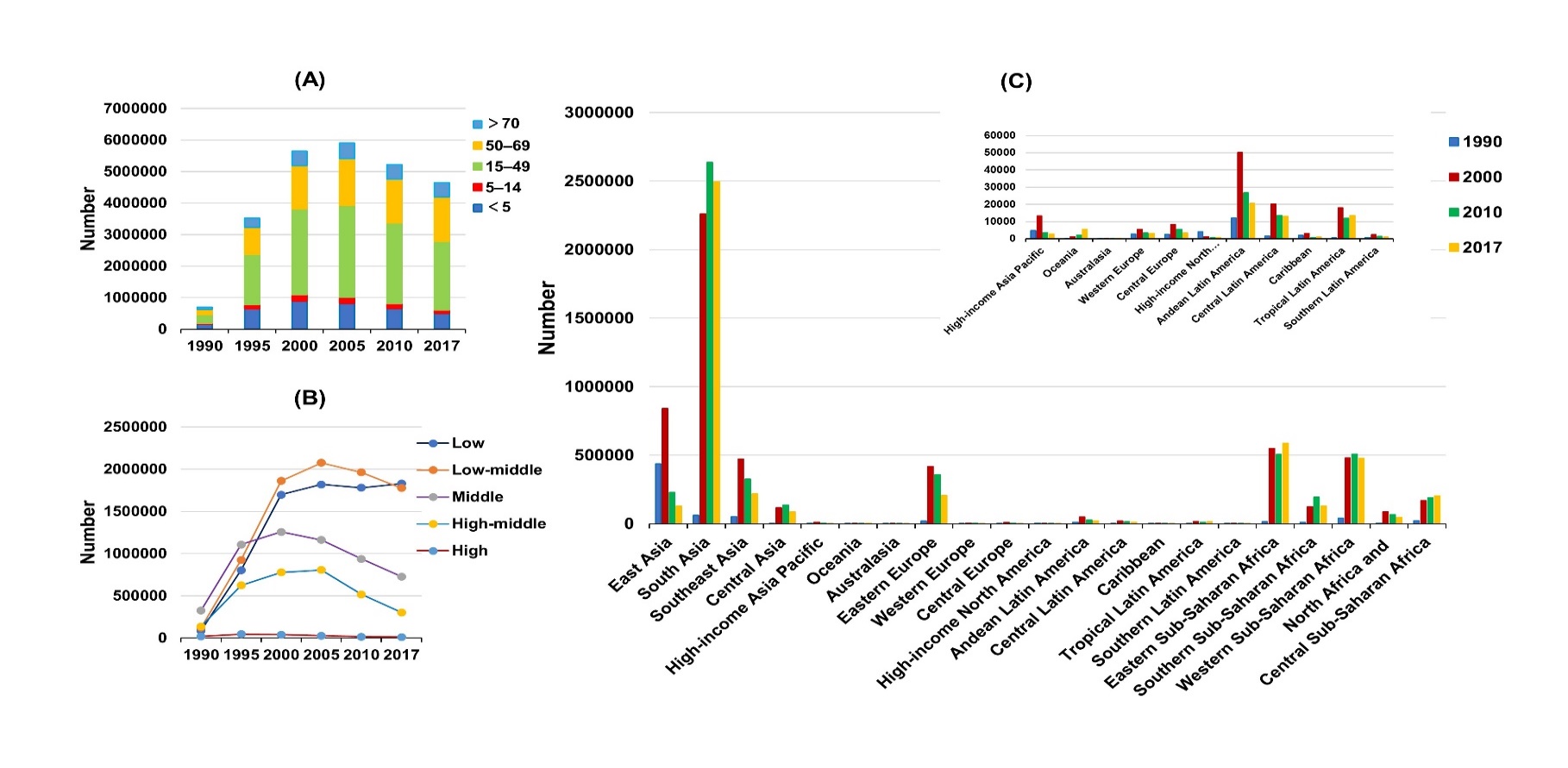
**

**Figure S4**. The distribution of ASR, percentage changes in absolute number, and EAPC of MDR-TB prevalence at a national level during 2000–2017. (A) the ASR of MDR-TB prevalence in 2017 in countries/territories; (B) the percentage changes in number of MDR-TB prevalence between 2000 and 2017 in countries/territories; (C) the EAPCs of MDR-TB prevalence in countries/territories from 2000 to 2017. Countries/territories with an extreme value were annotated. Abbreviations: MDR-TB: multidrug resistant tuberculosis; ASR, age-standardized rate; EAPC, estimated annual percentage change.


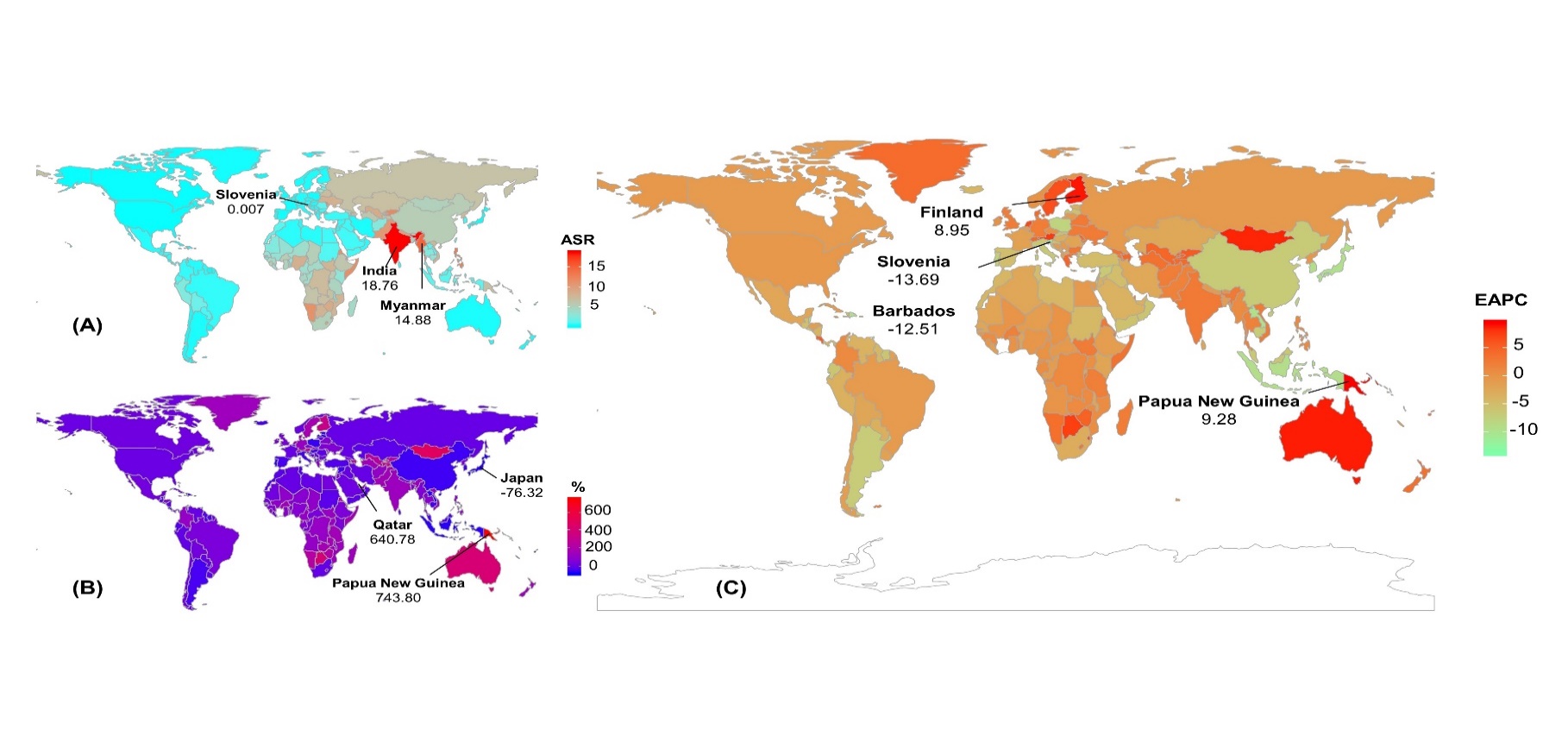


**Figure S5**. The distribution of ASR, percentage changes in absolute number, and EAPC of MDR-TB death at a national level during 2000–2017. (A) the ASR of MDR-TB death in 2017 in countries/territories; (B) the percentage changes in absolute number of MDR-TB death between 2000 and 2017 in countries/territories; (C) the EAPCs of MDR-TB death in countries/territories from 2000 to 2017. Countries/territories with an extreme value were annotated. Abbreviations: MDR-TB: multidrug resistant tuberculosis; ASR, age-standardized rate; EAPC, estimated annual percentage change.

**
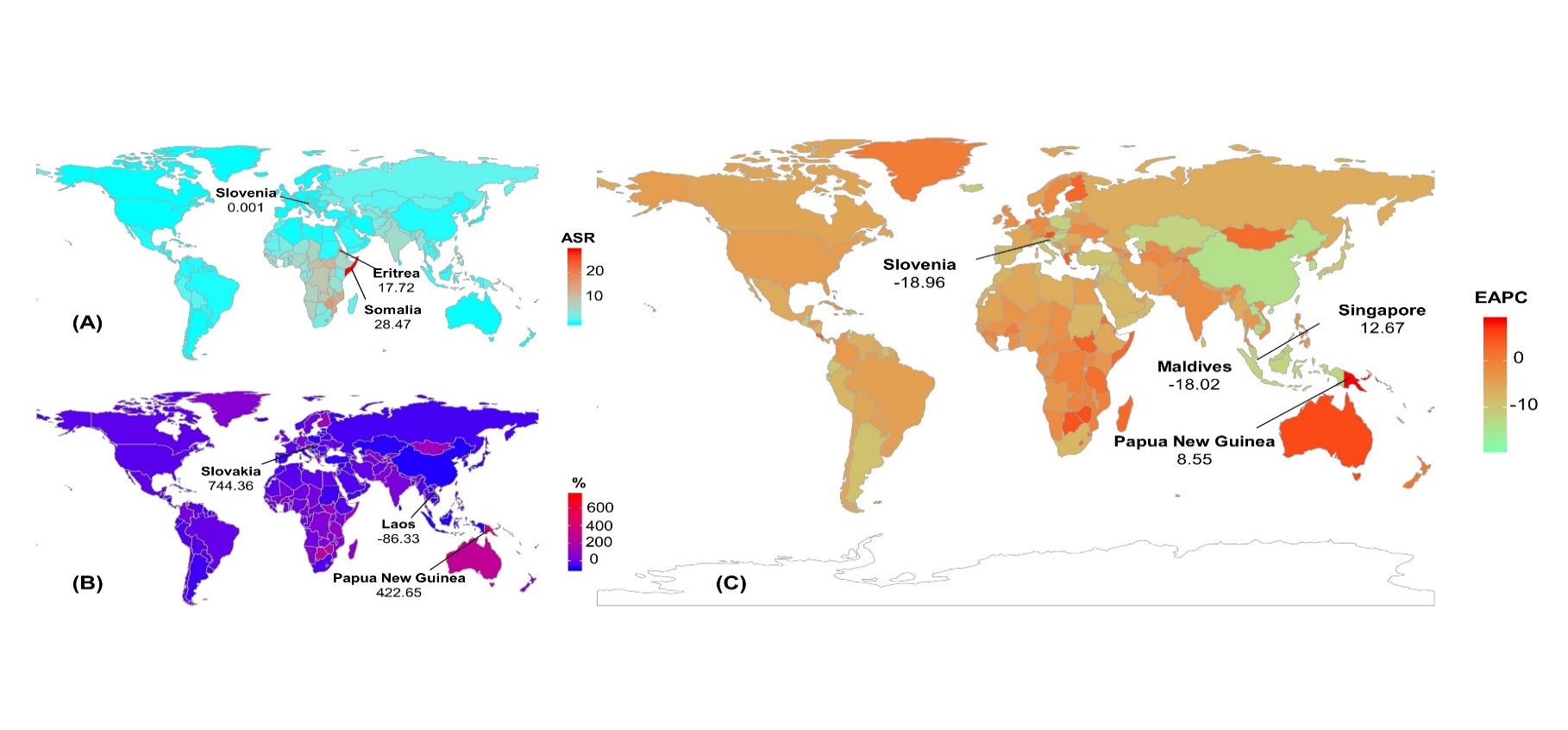
**

**Figure S6**. The distribution of ASR, percentage changes in absolute number, and EAPC of MDR-TB DALYs at a national level during 2000–2017. (A) the ASR of MDR-TB DALYs in 2017 in countries; (B) the percentage changes in absolute number of MDR-TB DALYs between 2000 and 2017 in countries; (C) the EAPCs of MDR-TB DALYs in countries/territories from 2000 to 2017. Countries/territories with an extreme value were annotated. Abbreviations: MDR-TB: multidrug resistant tuberculosis; DALYs, disability- adjusted life-years; ASR, age-standardized rate; EAPC, estimated annual percentage change.

**
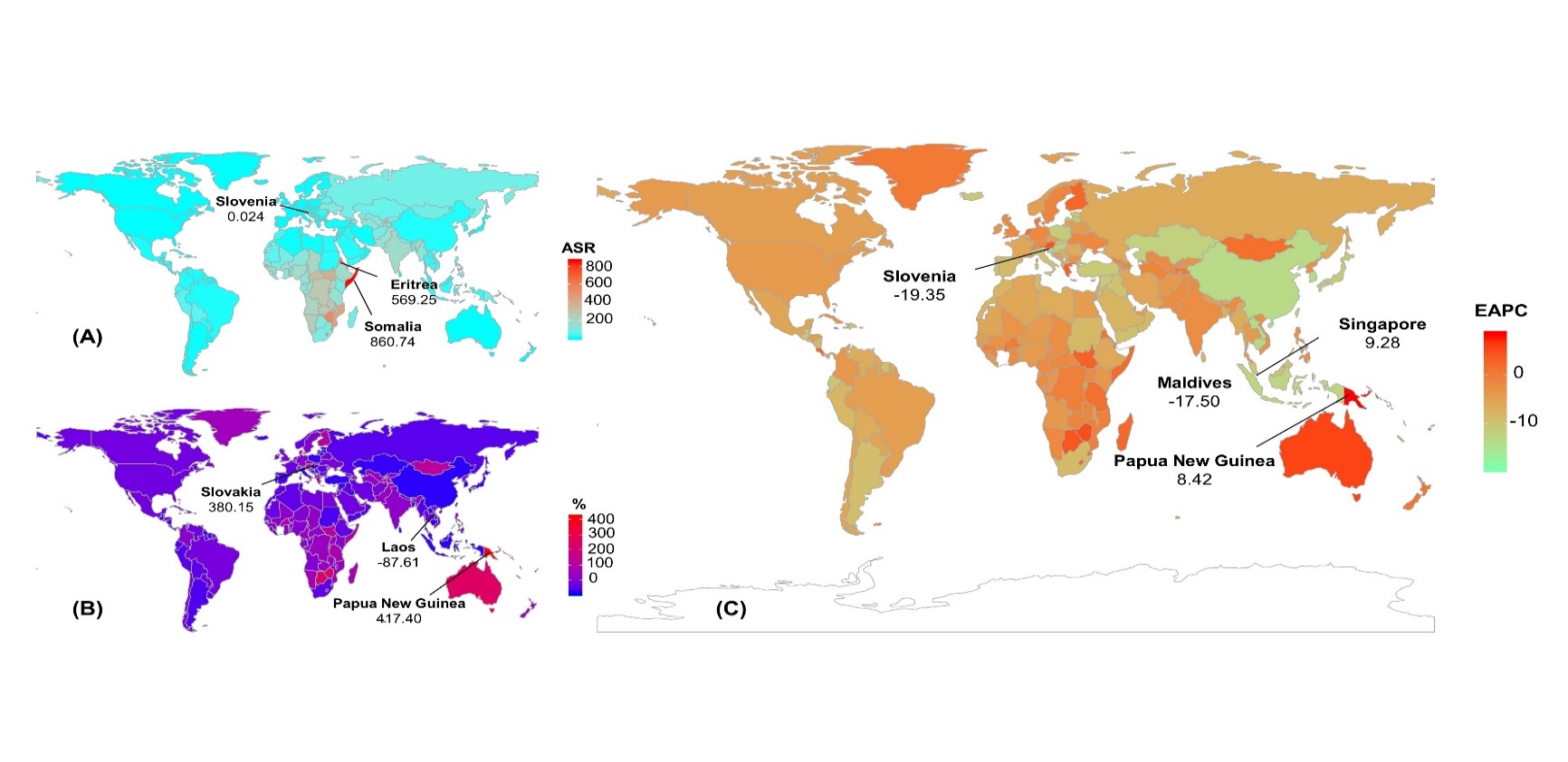
**

**Figure S7**. The distribution of ASR, percentage changes in absolute number, and EAPC of MDR-TB incidence at a national level during 1990–1999. (A) the ASR of MDR-TB incidence in 1999 in countries/territories; (B). the percentage changes in absolute incident number of MDR-TB between 1990 and 1999 in countries/territories; (C). the EAPCs of MDR-TB incidence in countries/territories from 1990 to 1999. Countries/territories with an extreme value were annotated. Abbreviations: MDR-TB: multidrug resistant tuberculosis; ASR, age-standardized rate; EAPC, estimated annual percentage change.

**
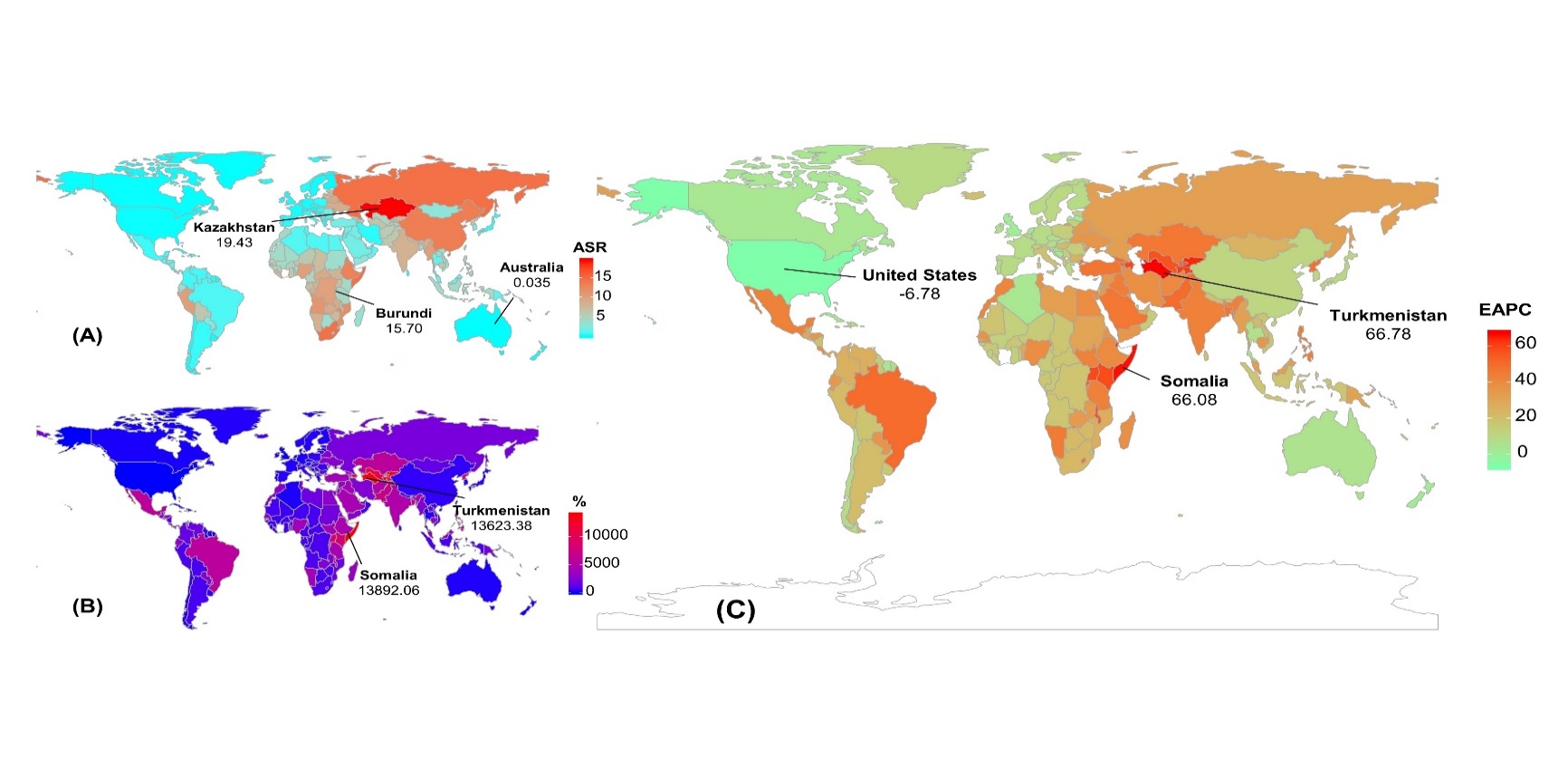
**

**Figure S8**. The distribution of ASR, percentage changes in absolute number, and EAPC of MDR-TB prevalence at a national level during 1990–1999. (A) the ASR of MDR-TB prevalence in 1999 in 195 countries/territories; (B). the percentage changes in absolute number of MDR-TB prevalence between 1990 and 1999 in countries; (C). the EAPCs of MDR-TB prevalence in countries from 1990 to 1999. Countries/territories with an extreme value were annotated. Abbreviations: MDR-TB: multidrug resistant tuberculosis; ASR, age-standardized rate; EAPC, estimated annual percentage change.

**
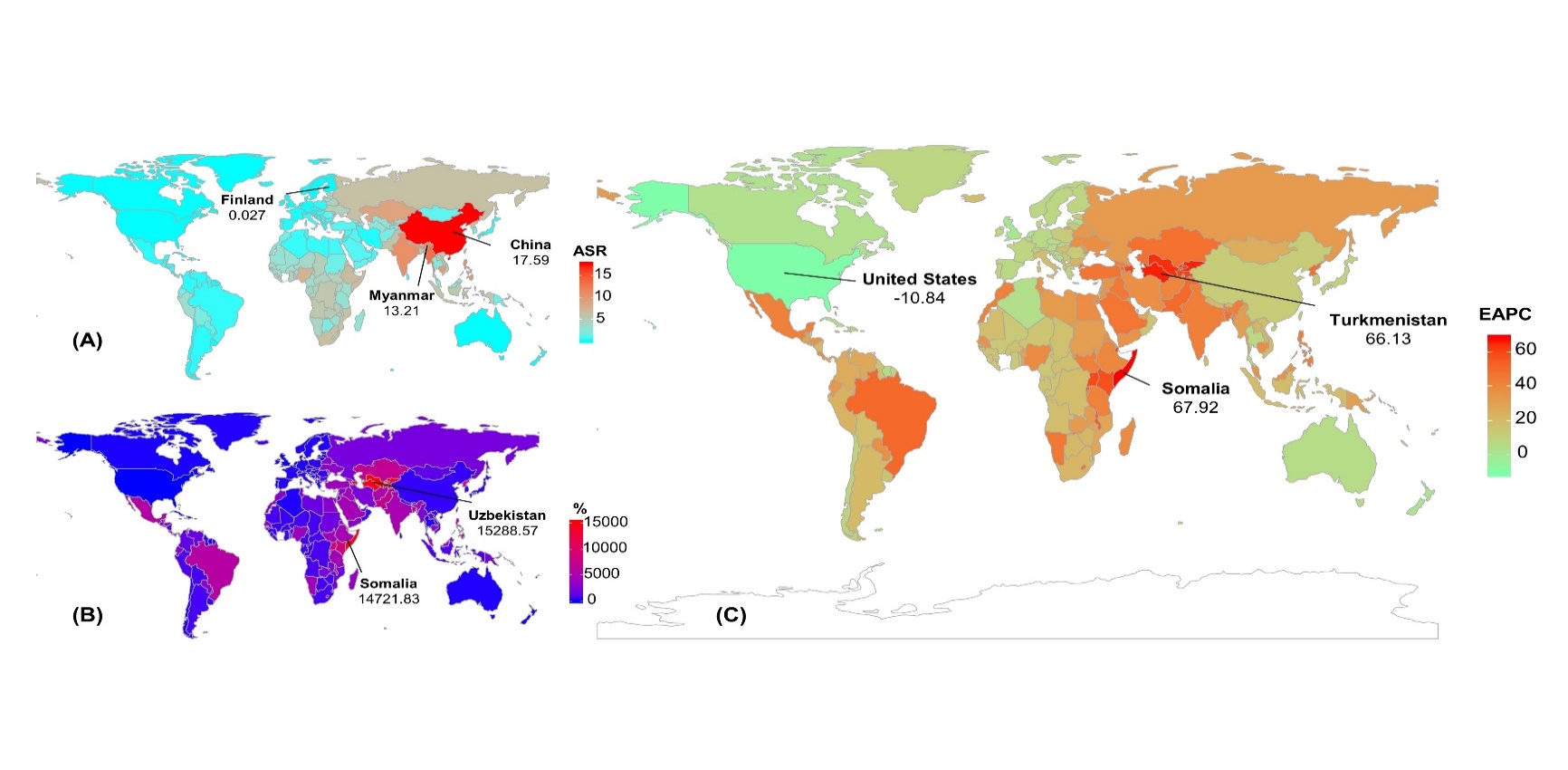
**

**Figure S9**. The distribution of ASR, percentage changes in absolute number, and EAPC of MDR-TB death at a national level during 1990–1999. (A) the ASR of MDR-TB death in 1999 in 195 countries/territories; (B). the percentage changes in absolute number of MDR-TB death between 1990 and 1999 in countries/territories; (C). the EAPCs of MDR-TB death in countries/territories from 1990 to 1999. Countries/territories with an extreme value were annotated. Abbreviations: MDR-TB: multidrug resistant tuberculosis; ASR, age-standardized rate; EAPC, estimated annual percentage change.

**
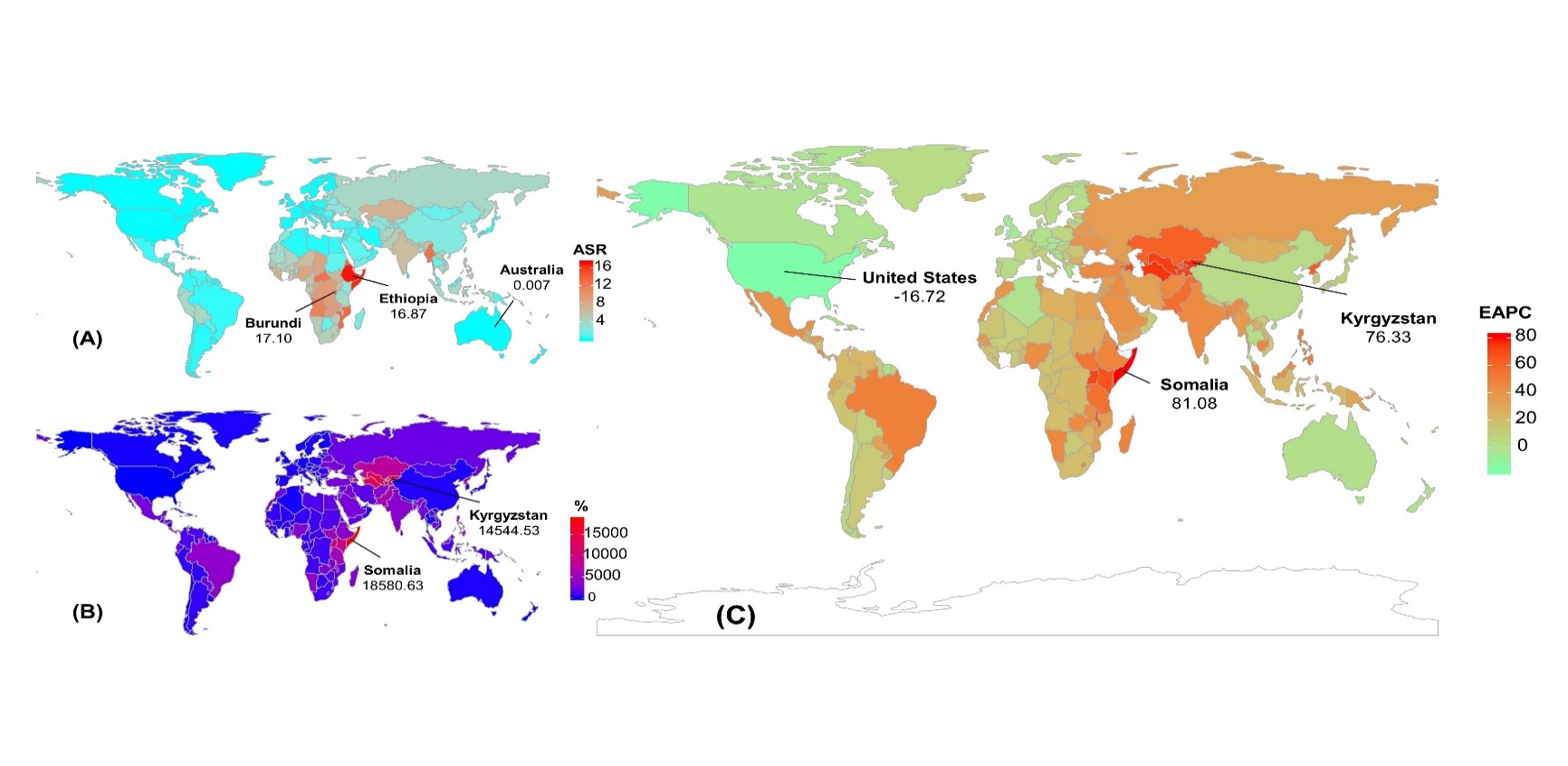
**

**Figure S10**. The distribution of ASR, percentage changes in absolute number, and EAPC of MDR-TB DALYs at a national level during 1990–1999. (A) the ASR of MDR-TB DALYs in 1999 in 195 countries; (B). the percentage changes in absolute number of MDR-TB DALYs between 1990 and 1999; (C). the EAPCs of MDR-TB DALYs in countries from 1990 to 1999. Countries/territories with an extreme value were annotated. Abbreviations: MDR-TB: multidrug resistant tuberculosis; DALYs, disability- adjusted life-years; ASR, age-standardized rate; EAPC, estimated annual percentage change.

**
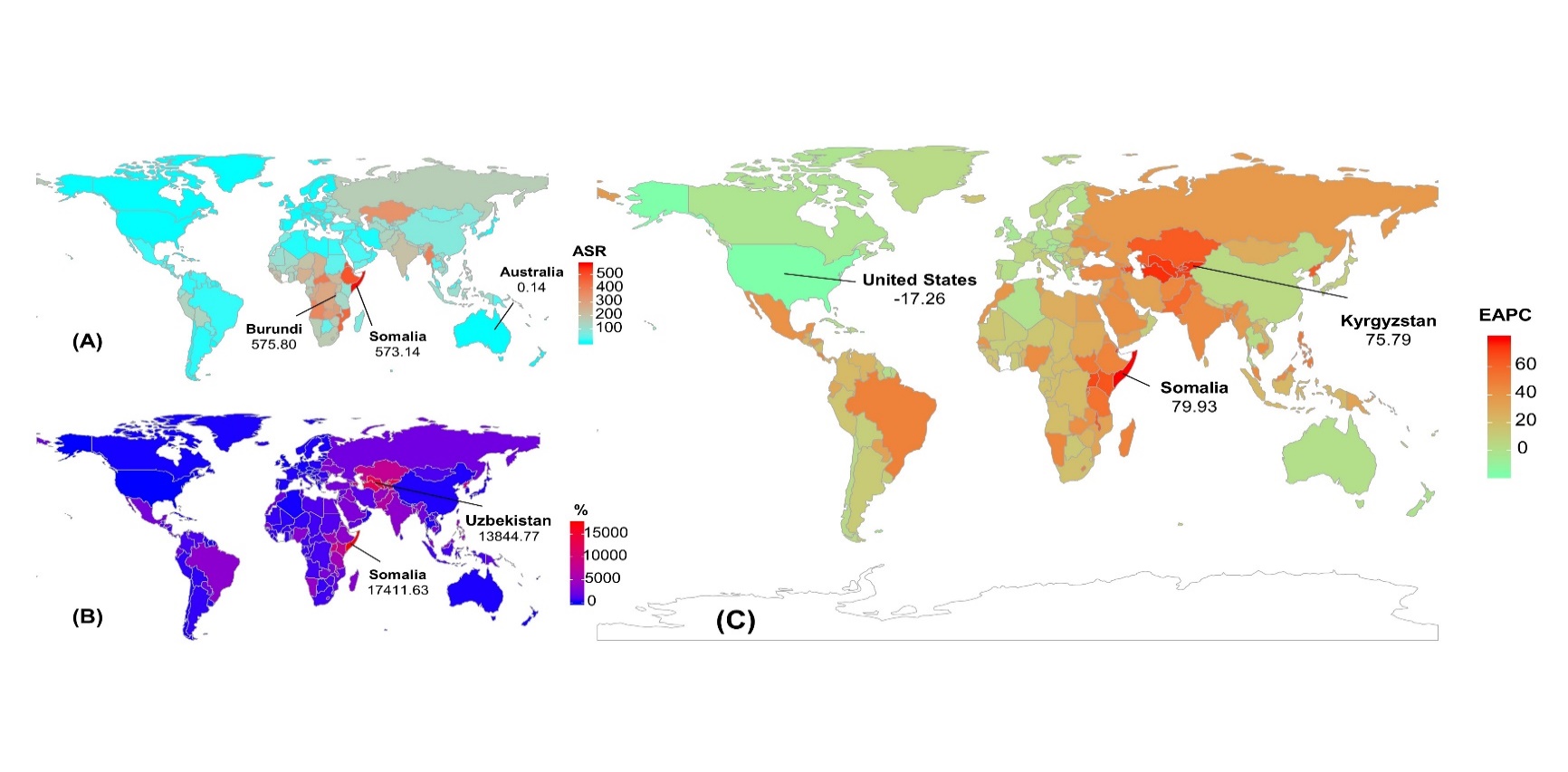
**

**Table S1**. the percentage changes in number and EAPCs of MDR-TB prevalence from 1990 to 2017 in global, sexes, SDI areas, and geographic regions

|  | 1999 | | 1990–1999 | | 2017 | | 2000–2017 | |
| --- | --- | --- | --- | --- | --- | --- | --- | --- |
| **Characteristics** | Number  ×10^3^ (95% UI) | ASR/100,000  (95% UI) | Change in  number (%) | EAPC  (95%CI) | Number  ×10^3^ (95% UI) | ASR/100,000  (95% UI) | Change in  number (%) | EAPC  (95%CI) |
| **Overall** | 402.46  (291.50–565.66) | 7.03  (5.11–9.78) | 552.27 | 17.57  (11.51–23.95) | 464.12  (229.12–863.33) | 5.85  (2.90–10.83) | 9.92 | -1.32  (-1.38–-1.26) |
| **Sex** |  |  |  |  |  |  |  |  |
| Male | 253.14  (183.13–354.58) | 9.14  (6.64–12.75) | 551.31 | 17.89  (12.58–23.46) | 285.82  (136.55–544.61) | 7.32  (3.51–14.02) | 6.74 | -1.45  (-1.58–-1.32) |
| Female | 149.33  (109.48–207.16) | 5.08  (3.74–7.03) | 553.90 | 17.01  (9.82–24.67) | 178.30  (91.88–317.29) | 4.48  (2.33–7.92) | 15.45 | -1.16  (-1.32–-0.99) |
| **SDI** |  |  |  |  |  |  |  |  |
| Low | 46.37  (21.6–103.01) | 7.58  (3.44–17) | 2389.96 | 35.01  (26.58–44.01) | 110.12  (43.45–254.99) | 11.67  (4.39–27.77) | 113.56 | 1.81  (1.62–2.00) |
| Low-middle | 79.55  (40.58–151.64) | 8.24  (4.23–15.65) | 1303.24 | 26.62  (19.35–34.32) | 146.75  (62.89–288.18) | 10.24  (4.28–20.33) | 68.84 | 0.77  (0.51–1.03) |
| Middle | 168.93  (105.46–246.31) | 11.09  (6.95–16.28) | 418.1 | 14.28  (8.33–20.56) | 143.47  (61.75–285.21) | 6.46  (2.79–12.90) | -17.23 | -3.13  (-3.26–-2.99) |
| High-middle | 97.56  (58.42–153.04) | 7.89  (4.72–12.41) | 419.02 | 15.03  (8.94–21.47) | 58.94  (27.01–140.61) | 3.65  (1.69–8.58) | -41.25 | -4.7  (-4.84–-4.55) |
| High | 6.28  (3.42–11.9) | 0.51  (0.28–0.96) | 235.42 | 10.87  (5.79–16.2) | 3.24  (1.52–7.65) | 0.23  (0.11–0.52) | -48.64 | -5.22  (-5.68–-4.75) |
| **Regions** |  |  |  |  |  |  |  |  |
| East Asia | 226.98  (156.62–320.61) | 17.18  (11.94–24.11) | 325.36 | 12.44  (6.43–18.79) | 93.95  (20.08–288.90) | 5.30  (1.14–16.37) | -58.67 | -6.74  (-6.91–-6.57) |
| South Asia | 99.71  (33.55–236.00) | 9.68  (3.28–23.01) | 4413.21 | 42.79  (32.29–54.12) | 260.63  (66.23–598.30) | 16.55  (4.17–37.91) | 129.76 | 2.34  (1.92–2.76) |
| Southeast Asia | 26.24  (12.90–61.71) | 6.03  (2.96–14.44) | 1073.87 | 22.65  (13.19–32.9) | 31.69  (18.50–50.16) | 4.90  (2.86–7.71) | 15.88 | -1.02  (-1.23–-0.80) |
| Central Asia | 2.75  (1.76–4.37) | 3.94  (2.54–6.24) | 8039.61 | 50.23  (34.78–67.46) | 6.59  (4.78–8.80) | 7.34  (5.33–9.80) | 99.59 | 1.97  (0.08–3.9) |
| High-income Asia Pacific | 1.28  (0.93–1.86) | 0.57  (0.42–0.84) | 265.61 | 11.3  (5.97–16.89) | 0.47  (0.11–1.33) | 0.17  (0.04–0.49) | -63.29 | -8.77  (-9.98–-7.55) |
| Oceania | 0.06  (0.01–0.19) | 0.97  (0.20–3.07) | 1465.33 | 28.34  (21.4–35.67) | 0.50  (0.25–0.87) | 4.82  (2.45–8.63) | 652.78 | 8.87  (7.41–10.36) |
| Australasia | 0.01  (0–0.02) | 0.03  (0.01–0.07) | 70.88 | 4.13  (1.32–7.02) | 0.03  (0.02–0.05) | 0.11  (0.06–0.17) | 332.75 | 7.58  (7.31–7.86) |
| Eastern Europe | 14.01  (9.80–19.41) | 5.71  (4.02–7.95) | 1780.53 | 31.72  (21.51–42.79) | 16.28  (11.00–22.26) | 6.91  (4.63–9.41) | 6.56 | 0.16  (-0.77–1.10) |
| Western Europe | 0.50  (0.421–0.584) | 0.12  (0.10–0.14) | 146.39 | 9.46  (7.77–11.18) | 0.48  (0.38–0.63) | 0.11  (0.08–0.14) | -6.45 | -1.63  (-2.15–-1.11) |
| Central Europe | 0.49  (0.34–0.79) | 0.36  (0.25–0.59) | 304.79 | 12.94  (5.64–20.74) | 0.34  (0.24–0.47) | 0.25  (0.17–0.34) | -29.80 | -2.69  (-3.53–-1.84) |
| High-income North America | 0.12  (0.10–0.14) | 0.03  (0.03–0.04) | -57.38 | -9.97  (-10.55–-9.39) | 0.11  (0.07–0.16) | 0.03  (0.02–0.04) | 3.17 | -0.53  (-1.09–0.03) |
| Andean Latin America | 1.08  (0.55–1.93) | 2.61  (1.32–4.62) | 832.53 | 23.43  (19–28.03) | 1.02  (0.74–1.52) | 1.70  (1.24–2.52) | -12.28 | -3.66  (-4.15–-3.16) |
| Central Latin America | 0.43  (0.25–0.80) | 0.26  (0.15–0.48) | 2350.01 | 31.95  (21–43.9) | 0.58  (0.27–1.23) | 0.23  (0.11–0.48) | 24.20 | -1.93  (-2.57–-1.29) |
| Caribbean | 0.06  (0.03–0.17) | 0.16  (0.07–0.43) | 170.59 | 8.67  (4.37–13.14) | 0.04  (0.02–0.09) | 0.08  (0.03–0.18) | -38.47 | -5.32  (-7.19–-3.41) |
| Tropical Latin America | 0.55  (0.15–1.36) | 0.33  (0.09–0.83) | 5402.06 | 49  (40.23–58.32) | 0.78  (0.18–2.12) | 0.33  (0.08–0.90) | 30.04 | -1.01  (-1.94–-0.08) |
| Southern Latin America | 0.11  (0.06–0.18) | 0.19  (0.11–0.33) | 477.23 | 18.54  (14.99–22.2) | 0.07  (0.02–0.19) | 0.10  (0.03–0.28) | -40.27 | -5.76  (-6.78–-4.74) |
| Eastern Sub-Saharan Africa | 8.65  (5.32–14.00) | 4.87  (3.00–8.09) | 4069.01 | 40.93  (30.36–52.36) | 18.14  (11.25–29.96) | 5.77  (3.60–9.97) | 91.60 | 0.30  (-0.21–0.81) |
| Southern Sub-Saharan Africa | 3.22  (1.69–7.06) | 5.50  (2.87–12.05) | 651.59 | 22.04  (20.98–23.1) | 4.79  (2.71–8.43) | 6.24  (3.57–10.79) | 29.98 | -0.80  (-2.04–0.45) |
| Western Sub-Saharan Africa | 10.65  (4.69–23.92) | 5.85  (2.56–13.13) | 1175.03 | 24.94  (17.44–32.92) | 18.77  (8.58–40.02) | 6.13  (2.77–13.12) | 61.50 | -0.44  (-1.04–0.15) |
| North Africa  and Middle East | 2.727  (1.696–4.051) | 0.71  (0.44–1.05) | 1811.55 | 33.7  (28.78–38.8) | 2.87  (1.89–4.74) | 0.49  (0.33–0.80) | -2.97 | -2.62  (-2.81–-2.44) |
| Central Sub-Saharan Africa | 2.831  (0.828–8.526) | 5.44  (1.63–16.56) | 686.24 | 18.15  (10.72–26.08) | 6.00  (1.43–16.40) | 6.66  (1.58–18.46) | 101.25 | 0.67  (0.45–0.89) |

MDR-TB: multidrug resistant tuberculosis; EAPC: estimated annual percentage change; ASR, age-standardized rate; CI, confidence interval; UI: uncertainty interval; SDI: socio-demographic index.

**Table S2**. the percentage changes in absolute number and EAPCs of death due to MDR-TB from 1990 to 2017 in global, sexes, SDI areas and geographic regions

|  | 1999 | | 1990–1999 | | 2017 | | 2000–2017 | |
| --- | --- | --- | --- | --- | --- | --- | --- | --- |
| **Characteristics** | Number  ×10^3^ (95% UI) | ASR/100,00  (95% UI) | Change in  number (%) | EAPC  (95%CI) | Number  ×10^3^ (95% UI) | ASR/100,000  (95% UI) | Change in  number (%) | EAPC  (95%CI) |
| **Overall** | 132.54  (89.22–205.25) | 1.60  (0.88–2.54) | 654.93 | 21.21  (15.96–26.69) | 126.89  (70.06–202.17) | 1.60  (0.88–2.54) | -10.17 | -3.30  (-3.56–-3.04) |
| **Sex** |  |  |  |  |  |  |  |  |
| Male | 85.15  (58.01–128.54) | 3.34  (2.29–5.01) | 690.65 | 21.52  (16.15–27.15) | 80.29  (45.46–127.64) | 2.10  (1.19–3.33) | -5.71 | -3.52  (-3.8–-3.23) |
| Female | 47.40  (30.69–74.57) | 1.68  (1.09–2.64) | 598.26 | 20.40  (15.42–25.6) | 46.60  (24.63–77.36) | 1.14  (0.61–1.89) | -1.68 | -3.01  (-3.27–-2.76) |
| **SDI** |  |  |  |  |  |  |  |  |
| Low | 36.44  (19.48–69.11) | 7.22  (3.83–13.8) | 1761.95 | 35.34  (28.34–42.72) | 47.85  (24.33–89.15) | 6.08  (3.03–11.61) | 21.77 | -1.74  (-1.95–-1.53) |
| Low-middle | 42.56  (23.20–77.57) | 5.26  (2.85–9.56) | 1372.7 | 31.39  (24.94–38.18) | 48.93  (24.59–83.67) | 3.90  (1.95–6.74) | 5.21 | -2.72  (-3.07–-2.36) |
| Middle | 32.44  (21.11–48.79) | 2.44  (1.6–3.67) | 304.11 | 12  (6.94–17.3) | 21.53  (11.29–35.34) | 0.99  (0.52–1.63) | -35.66 | -5.79  (-6.07–-5.51) |
| High-middle | 18.93  (14.3–24.36) | 1.57  (1.18–2.03) | 405.05 | 14.97  (8.97–21.3) | 7.89  (5.84–10.47) | 0.45  (0.33–0.60) | -60.4 | -8.14  (-8.79–-7.49) |
| High | 1.75  (1.29–2.40) | 0.12  (0.09–0.16) | 135.00 | 6.54  (2.54–10.7) | 0.60  (0.39–0.94) | 0.03  (0.02–0.04) | -63.98 | -9.19  (-9.98–-8.39) |
| **Regions** |  |  |  |  |  |  |  |  |
| East Asia | 24.00  (156.62–320.61) | 2.04  (1.33–2.88) | 117.01 | 4.43  (-1.06–10.24) | 3.81  (1.07–9.79) | 0.20  (0.06–0.51) | -83.17 | -13.57  (-14.21–-12.92) |
| South Asia | 50.72  (33.55–236) | 5.89  (1.98–13.57) | 3227.97 | 44.51  (35.74–53.85) | 71.98  (21.83–146.91) | 5.17  (1.55–10.55) | 26.67 | -1.95  (-2.34–-1.56) |
| Southeast Asia | 13.67  (12.90–61.71) | 3.89  (1.92–8.66) | 867.21 | 23.83  (15.94–32.25) | 7.03  (4.25–11.50) | 1.25  (0.77–2.04) | -47.89 | -6.35  (-6.83–-5.87) |
| Central Asia | 2.10  (1.76–4.37) | 3.24  (2.04–4.95) | 8156.05 | 62.35  (47.24–79.01) | 1.91  (1.43–2.36) | 2.14  (1.60–2.63) | -22.35 | -4.52  (-6.04–-2.98) |
| High-income Asia Pacific | 0.68  (0.93–1.86) | 0.26  (0.19–0.38) | 292.01 | 11.22  (6.95–15.67) | 0.19  (0.05–0.52) | 0.04  (0.01–0.10) | -69.35 | -11.65  (-12.9–-10.39) |
| Oceania | 0.02  (0.01–0.19) | 0.49(  0.09–1.61) | 1125.74 | 30.06  (23.96–36.47) | 0.12  (0.06–0.21) | 1.40  (0.71–2.43) | 377.19 | 8.14  (6.10–10.22) |
| Australasia | 0  (0–0.02) | 0.01  (0–0.02) | 54.70 | 0.97  (-2.07–4.1) | 0.01  (0–0.01) | 0.01  (0.01–0.02) | 219.36 | 4.51  (4.11–4.91) |
| Eastern Europe | 9.20  (9.80–19.41) | 3.41  (2.54–4.40) | 1651.72 | 35.61  (24.49–47.72) | 5.27  (3.96–6.51) | 1.88  (1.41–2.33) | -49.25 | -5.07  (-6.13–-3.99) |
| Western Europe | 0.29  (0.42–0.58) | 0.04  (0.03–0.05) | 113.03 | 5.49  (3.51–7.51) | 0.20  (0.15–0.26) | 0.02  (0.01–0.03) | -34.55 | -5.21  (-5.71–-4.71) |
| Central Europe | 0.25  (0.34–0.79) | 0.16  (0.10–0.28) | 227.03 | 12.43  (5.24–20.12) | 0.10  (0.07–0.14) | 0.06  (0.04–0.08) | -57.59 | -7.01  (-7.87–-6.13) |
| High-income North America | 0.05  (0.10–0.14) | 0.01  (0.01–0.02) | -68.42 | -15.91  (-18.52–-13.22) | 0.03  (0.02–0.05) | 0.01  (0–0.01) | -33.10 | -4.12  (-4.88–-3.36) |
| Andean Latin America | 1.18  (0.55–1.93) | 3.34  (1.72–5.63) | 390.27 | 16.64  (11.88–21.59) | 0.59  (0.39–0.88) | 1.05  (0.70–1.57) | -48.12 | -7.42  (-8.02–-6.81) |
| Central Latin America | 0.56  (0.25–0.80) | 0.41  (0.22–0.76) | 1345.62 | 30.59  (21.66–40.17) | 0.40  (0.20–0.76) | 0.17  (0.08–0.32) | -26.92 | -5.72  (-6.31–-5.13) |
| Caribbean | 0.08  (0.03–0.17) | 0.20  (0.07–0.55) | 91.26 | 4.10  (0.09–8.27) | 0.03  (0.01–0.07) | 0.05  (0.02–0.14) | -61.95 | -7.45  (-9.39–-5.47) |
| Tropical Latin America | 0.42  (0.15–1.35) | 0.30  (0.07–0.76) | 3685.82 | 48.38  (41.17–55.95) | 0.40  (0.09–1.00) | 0.17  (0.04–0.43) | -15.10 | -4.37  (-5.21–-3.53) |
| Southern Latin America | 0.08  (0.06–0.18) | 0.15  (0.08–0.25) | 292.64 | 11.96  (8.07–16) | 0.03  (0.01–0.09) | 0.04  (0.02–0.11) | -55.76 | -8.10  (-9.05–-7.13) |
| Eastern Sub-Saharan Africa | 11.61  (5.32–14.00) | 9.35  (5.30–16.25) | 3718.39 | 48.86  (39.59–58.73) | 14.03  (8.81–21.30) | 7.11  (4.42–10.83) | 14.88 | -1.81  (-2.3–-1.31) |
| Southern Sub-Saharan Africa | 2.10  (1.69–7.06) | 4.66  (2.35–9.59) | 730.78 | 21.24  (18.49–24.06) | 3.15  (1.52–5.80) | 4.94  (2.41–8.97) | 9.88 | -2.96  (-4.79–-1.10) |
| Western Sub-Saharan Africa | 10.06  (4.69–23.91) | 7.49  (3.42–16.16) | 1860.77 | 26.09  (19.28–33.29) | 11.45  (5.60–21.87) | 5.39  (2.65–10.26) | 2.62 | -3.24  (-3.73–-2.74) |
| North Africa  and Middle East | 1.88  (1.70–4.05) | 0.69  (0.38–1.18) | 1608.82 | 36.07  (30.69–41.67) | 1.18  (0.72–2.00) | 0.26  (0.16–0.42) | -39.55 | -6.53  (-6.88–-6.19) |
| Central Sub-Saharan Africa | 3.60  (0.83–8.53) | 9.74  (2.87–28.27) | 655.26 | 20.83  (14.32–27.72) | 4.98  (1.28–13.33) | 8.29  (2.06–22.15) | 34.32 | -1.03  (-1.18–-0.87) |

MDR-TB: multidrug resistant tuberculosis; DALYs: disability-adjusted life-years; EAPC: estimated annual percentage change; ASR, age-standardized rate; CI, confidence interval; UI: uncertainty interval; SDI: socio-demographic index.

**Table S3**. the percentage changes in absolute number and EAPCs of DALYs due to MDR-TB from 1990 to 2017 in global, sexes, SDI areas and geographic regions

|  | 1999 | | 1990–1999 | | 2017 | | 2000–2017 | |
| --- | --- | --- | --- | --- | --- | --- | --- | --- |
| **Characteristics** | Number  ×10^3^ (95% UI) | ASR/100,000  (95% UI) | Change in  number (%) | EAPC  (95%CI) | Number  ×10^3^ (95% UI) | ASR/100,000  (95% UI) | Change in  number (%) | EAPC  (95%CI) |
| **Overall** | 5316.10  (3629.10–8190.46) | 91.50  (62.35–140.86) | 659.48 | 21.90  (16.55–27.50) | 4647.99  (2663.04–7224.23) | 59.02  (33.99–91.37) | -17.71 | -3.32  (-3.59–-3.06) |
| **Sex** |  |  |  |  |  |  |  |  |
| Male | 3304.35  (2281.63–4971.77) | 115.93  (79.90–175.26) | 691.75 | 22.24  (16.76–27.97) | 2908.71  (1705.8–4532.12) | 74.01  (43.52–115.1) | -17.50 | -3.39  (-3.68–-3.10) |
| Female | 2011.75  (1306.89–3193.91) | 68.25  (44.27–108.37) | 611.83 | 21.25  (16.12–26.62) | 1739.28  (971.36–2783.97) | 44.63  (25.28–70.51) | -18.05 | -3.24  (-3.48–-2.99) |
| **SDI** |  |  |  |  |  |  |  |  |
| Low | 1583.62  (869.82–2958.63) | 235.92  (126.45–447.21) | 1685.9 | 35.29  (28.27–42.69) | 1828.11  (966.92–3315.25) | 187.57  (95.9–348.11) | 7.73 | -1.98  (-2.15–-1.82) |
| Low-middle | 1710.17  (952.93–3086.74) | 167.70  (92.24–305.94) | 1293.22 | 30.75  (24.36–37.47) | 1776.81  (937.92–2970.72) | 120.75  (61.88–205.40) | -4.58 | -2.83  (-3.19–-2.48) |
| Middle | 1228.33  (802.7–1849.21) | 78.94  (51.7–118.94) | 277.29 | 11.96  (6.85–17.31) | 725.94  (374.76–1187.1) | 32.39  (16.84–52.79) | -42.23 | -5.70  (-5.99–-5.4) |
| High-middle | 735.98  (559.92–942.02) | 59.15  (45.04–76.3) | 441.88 | 16.54  (10.36–23.06) | 302.43  (222.97–396.22) | 17.76  (13.05–23.42) | -61.12 | -7.84  (-8.54–-7.13) |
| High | 41.85  (30.3–57.99) | 3.17  (2.27–4.41) | 112.37 | 5.61  (1.39–10.02) | 11.56  (7.67–18.10) | 0.65  (0.44–1.01) | -70.79 | -9.56  (-10.27–-8.85) |
| **Regions** |  |  |  |  |  |  |  |  |
| East Asia | 910.89  (591.4–1295.6) | 72.32  (47.06–103.37) | 108.05 | 5.06  (-0.61–11.07) | 130.17  (33.96–351.32) | 7.19  (1.89–19.52) | -84.55 | -13.02  (-13.58–-12.46) |
| South Asia | 2027.32  (696.79–4674.64) | 188.14  (63.79–433.72) | 3136.15 | 44.18  (35.51–53.39) | 2495.54  (787.09–5092.26) | 157.24  (49.04–321.63) | 10.36 | -2.15  (-2.53–-1.76) |
| Southeast Asia | 483.98  (229.66–1086.61) | 110.25  (52.93–249.28) | 840.44 | 24.54  (16.38–33.26) | 217.81  (129.55–364.22) | 34.29  (20.53–57.28) | -54.02 | -6.55  (-6.96–-6.14) |
| Central Asia | 99.35  (62.77–151.56) | 144.33  (91.45–220.19) | 8030.83 | 62.87  (47.83–79.44) | 85.89  (65.06–105.22) | 91.64  (69.32–112.19) | -26.23 | -4.81  (-6.34–-3.25) |
| High-income Asia Pacific | 14.44  (10.37–21.62) | 5.98  (4.27–9.03) | 208.06 | 8.96  (4.86–13.24) | 2.84  (0.65–7.50) | 0.78  (0.18–2.15) | -78.56 | -12.62  (-13.85–-11.38) |
| Oceania | 1.09  (0.18–3.77) | 16.89  (2.92–57.28) | 1175.89 | 30.49  (24.51–36.76) | 5.53  (2.66–9.53) | 50.87  (24.87–87.99) | 376.81 | 8.32  (6.35–10.33) |
| Australasia | 0.05  (0.02–0.10) | 0.17  (0.08–0.38) | 38.21 | 0.39  (-2.44–3.30) | 0.13  (0.08–0.20) | 0.31  (0.18–0.48) | 185.44 | 4.49  (4.12–4.86) |
| Eastern Europe | 368.26  (274.92–472.87) | 143.54  (107.28–184.41) | 1807.91 | 36.88  (25.58–49.19) | 206.88  (154.87–257.02) | 79.59  (59.45–98.82) | -50.53 | -5.09  (-6.21–-3.95) |
| Western Europe | 5.42  (4.37–6.59) | 0.93  (0.75–1.12) | 90.16 | 4.65  (2.63–6.7) | 3.08  2.32–4.04) | 0.41  (0.31–0.54) | -44.61 | -5.44  (-5.93–-4.94) |
| Central Europe | 8.56  (5.14–15.21) | 6.16  (3.63–11.12) | 225.37 | 12.83  (5.18–21.04) | 3.27  (2.10–4.65) | 2.16  (1.37–3.10) | -60.68 | -7.16  (-8.06–-6.26) |
| High–income North America | 1.18  (0.92–1.48) | 0.31  (0.24–0.39) | -71.47 | -16.58  (-19.16–-13.92) | 0.72  (0.47–1.08) | 0.14  (0.09–0.22) | -33.16 | -3.87  (-4.59–-3.14) |
| Andean Latin America | 52.39  (26.31–88.8) | 124.44  (62.92–211.48) | 330.68 | 15.64  (11.09–20.37) | 20.68  (13.75–30.79) | 34.83  (23.12–51.86) | -58.86 | -8.08  (-8.69–-7.47) |
| Central Latin America | 20.92  (11.71–38.09) | 12.79  (7.03–23.45) | 1200.35 | 29.84  (20.82–39.54) | 13.04  (6.37–24.68) | 5.21  (2.54–9.83) | -35.66 | -5.71  (-6.28–-5.13) |
| Caribbean | 3.48  (0.99–9.83) | 8.92  (2.58–25.1) | 74.41 | 3.55  (-0.36–7.62) | 1.03  (0.32–2.85) | 2.22  (0.68–6.23) | -67.35 | -7.73  (-9.65–-5.78) |
| Tropical Latin America | 16.51  (4.09–41.9) | 10.44  (2.59–26.55) | 3210.22 | 47.41  (40.17–55.02) | 13.47  (3.17–33.66) | 5.70  (1.35–14.21) | -24.98 | -4.42  (-5.3–-3.54) |
| Southern Latin America | 2.55  (1.42–4.36) | 4.67  (2.59–7.98) | 254.86 | 11.21  (7.31–15.25) | 0.99  (0.33–2.65) | 1.35  (0.44–3.69) | -59.86 | -8.2  (-9.11–-7.29) |
| Eastern Sub-Saharan Africa | 527.41  (310.53–884.27) | 305.62  (175.1–522.12) | 3504.25 | 47.75  (38.48–57.65) | 588.37  (373.25–888.01) | 219.14  (137.5–332.37) | 6.75 | -2.03  (-2.53–-1.53) |
| Southern Sub-Saharan Africa | 90.00  (45.74–183.67) | 168.85  (85.68–340.88) | 648.65 | 19.64  (16.76–22.59) | 127.96  (61.63–245.11) | 177.79  (85.65–337.01) | 2.36 | -3.40  (-5.33–-1.44) |
| Western Sub-Saharan Africa | 433.95  (200.64–944.19) | 218.73  (102.49–474.07) | 926.07 | 25.15  (18.38–32.31) | 477.57  (231.46–943.68) | 149.98  (73.14–289.23) | -0.77 | -3.47  (-3.94–-3.01) |
| North Africa  and Middle East | 84.97  (45.48–147.39) | 22.93  (12.33–39.76) | 1542.92 | 36.41  (30.74–42.33) | 48.13  (27–88.54) | 8.71  (5.03–15.69) | -44.62 | -6.32  (-6.68–-5.96) |
| Central Sub-Saharan Africa | 163.37  (46.52–457.54) | 303.42  (88.59–869.75) | 643.87 | 20.63  (14.08–27.56) | 204.89  (53.23–549.80) | 243.74  (63.12–648.80) | 21.90 | -1.36  (-1.52–-1.2) |

MDR-TB: multidrug resistant tuberculosis; DALYs: disability-adjusted life-years; EAPC: estimated annual percentage change; ASR, age-standardized rate; CI, confidence interval; UI: uncertainty interval; SDI: socio-demographic index.

**Table S4**. the percentage change in absolute number and EAPCs of MDR-TB at national level and both sexes from 1990 to 1999

| **Characteristics** | **Incidence** | | **Prevalence** | | | **Death** | | | **DALYs** | | | | |
| --- | --- | --- | --- | --- | --- | --- | --- | --- | --- | --- | --- | --- | --- |
|  | Change in  number (%) | EAPC  (95%CI) | Change in number (%) | EAPC  (95%CI) | | Change in  number (%) | | EAPC  (95%CI) | | Change in  number (%) | | EAPC  (95%CI) | |
| Afghanistan | 5058.42 | 40.6  (30.66–51.28) | 5199.92 | | 40.98  (31.06–51.66) | 3549.16 | 46.17  (37.59–55.28) | | 3911.90 | | 45.19  (36.97–53.89) | |  |
| Albania | 413.67 | 14.35  (6.65–22.62) | 422.04 | | 14.38  (6.72–22.59) | 168.53 | 8.15  (1.64–15.09) | | 127.67 | | 6.67  (0.13–13.63) | |  |
| Algeria | 81.83 | 3.79  (1.98–5.63) | 82.78 | | 3.91  (2.09–5.75) | 24.78 | 0.13  (-1.07–1.35) | | 10.92 | | 0.05  (-1.14–1.26) | |  |
| American Samoa | 459.56 | 14.15  (6.25–22.64) | 442.88 | | 13.5  (5.43–22.19) | 289.08 | 13.97  (6.04–22.5) | | 299.42 | | 14.07  (6.21–22.5) | |  |
| Andorra | 55.96 | 2.37  (-1.15–6.02) | 80.16 | | 3.46  (-0.32–7.38) | 67.08 | 0.82  (-3.74–5.59) | | 52.42 | | 0.87  (-3.56–5.51) | |  |
| Angola | 698.08 | 17.91  (10.88–25.37) | 733.40 | | 18.5  (11.53–25.91) | 636.57 | 19.74  (13.48–26.33) | | 634.42 | | 19.48  (13.15–26.17) | |  |
| Antigua and Barbuda | 213.34 | 8.74  (4.08–13.61) | 214.01 | | 8.92  (4.18–13.89) | 114.16 | 5.17  (1.04–9.46) | | 111.85 | | 4.62  (0.53–8.88) | |  |
| Argentina | 610.83 | 21.29  (17.69–24.99) | 578.44 | | 20.4  (16.25–24.7) | 365.12 | 13.64  (9–18.48) | | 315.47 | | 12.77  (8.2–17.53) | |  |
| Armenia | 6764.20 | 52.6  (39.71–66.68) | 7253.84 | | 50.92  (35.86–67.66) | 7165.75 | 61.38  (46.69–77.54) | | 6228.22 | | 59.67  (45.29–75.46) | |  |
| Australia | 93.08 | 5.52  (2.26–8.89) | 93.13 | | 5.26  (2.01–8.61) | 73.29 | 1.67  (-1.97–5.45) | | 55.75 | | 1.21  (-2.23–4.77) | |  |
| Austria | 87.45 | 6.91  (6.27–7.55) | 65.88 | | 5.29  (4.63–5.94) | -17.01 | -5.25  (-7.13–-3.33) | | -17.43 | | -5.24  (-7.12–-3.32) | |  |
| Azerbaijan | 11125.57 | 59.74  (45.44–75.46) | 11351.30 | | 59.84  (45.46–75.64) | 10098.86 | 69.59  (55.5–84.95) | | 9638.74 | | 69.29  (55.58–84.2) | |  |
| Bahrain | 3736.16 | 41.48  (33.36–50.09) | 3720.77 | | 41.31  (33.07–50.06) | 2862.73 | 41.85  (34.1–50.04) | | 2974.58 | | 41.87  (34.25–49.91) | |  |
| Bangladesh | 5159.30 | 46.89  (36.79–57.73) | 5424.69 | | 48.04  (37.85–58.98) | 3504.97 | 49.29  (40.47–58.66) | | 3348.69 | | 48.75  (40.01–58.03) | |  |
| Barbados | 122.51 | 7.03  (1.08–13.33) | 127.88 | | 7.17  (1.01–13.69) | 63.98 | 4.33  (-1.05–10.01) | | 61.61 | | 4.42  (-1.04–10.18) | |  |
| Belarus | 2600.87 | 34.47  (21.19–49.2) | 2682.80 | | 34.67  (21.2–49.64) | 2554.13 | 39.1  (27.14–52.2) | | 2754.62 | | 40.03  (28.02–53.16) | |  |
| Belgium | 144.22 | 9.35  (7.24–11.51) | 144.19 | | 8.87  (5.93–11.9) | 109.67 | 5.59  (2.4–8.88) | | 98.65 | | 5.19  (2.12–8.36) | |  |
| Belize | 200.44 | 8.73  (3.73–13.96) | 210.08 | | 9.07  (4.04–14.33) | 173.69 | 8.7  (4.54–13.02) | | 148.57 | | 8.19  (3.92–12.64) | |  |
| Benin | 1158.56 | 29.69  (28.69–30.69) | 1157.09 | | 29.77  (28.76–30.79) | 874.19 | 24.96  (23.32–26.62) | | 833.45 | | 24.42  (22.78–26.08) | |  |
| Bermuda | 188.76 | 9.55  (4.19–15.18) | 207.08 | | 10.13  (4.86–15.67) | 66.41 | 2.66  (-2.19–7.76) | | 58.36 | | 2.54  (-2.53–7.89) | |  |
| Bhutan | 690.29 | 20.6  (13.38–28.29) | 705.34 | | 20.44  (13.16–28.19) | 532.06 | 18.65  (11.92–25.78) | | 476.13 | | 18.25  (11.47–25.46) | |  |
| Bolivia | 614.29 | 18.19  (12.72–23.93) | 620.62 | | 18.62  (13.06–24.46) | 295.52 | 11.33  (6.03–16.89) | | 258.58 | | 10.62  (5.31–16.2) | |  |
| Bosnia and Herzegovina | 38.01 | 3.26  (0.55–6.06) | 55.43 | | 3.96  (0.85–7.16) | 4.43 | -3.49  (-8.5–1.81) | | 2.07 | | -3.31  (-8.45–2.11) | |  |
| Botswana | 590.55 | 22.28  (19.6–25.02) | 535.28 | | 20.72  (18.18–23.31) | 387.24 | 13.87  (11.38–16.41) | | 377.84 | | 13.76  (11.27–16.3) | |  |
| Brazil | 5663.46 | 49.86  (41.1–59.16) | 5752.34 | | 50.34  (41.51–59.73) | 3887.56 | 49.92  (42.62–57.59) | | 3354.05 | | 48.66  (41.38–56.32) | |  |
| Brunei | 380.65 | 12.05  (5.34–19.18) | 380.62 | | 12.09  (5.37–19.23) | 265.41 | 10.6  (4.53–17.03) | | 264.25 | | 10.44  (4.52–16.71) | |  |
| Bulgaria | 853.84 | 25.49  (17.94–33.52) | 768.79 | | 22.78  (14.32–31.86) | 854.64 | 27.7  (18.91–37.14) | | 809.62 | | 27.81  (19.13–37.14) | |  |
| Burkina Faso | 540.71 | 16.68  (10.25–23.48) | 562.25 | | 17.28  (10.82–24.13) | 396.94 | 15.4  (9.11–22.06) | | 373.48 | | 14.89  (8.62–21.53) | |  |
| Burundi | 3135.20 | 38.87  (28.02–50.63) | 3265.19 | | 39.51  (28.58–51.36) | 3566.73 | 50.2  (40.62–60.43) | | 3321.74 | | 48.81  (39.06–59.24) | |  |
| Cambodia | 3202.28 | 35.7  (22.82–49.94) | 3638.68 | | 37.59  (24.84–51.64) | 2966.12 | 46.41  (35.21–58.55) | | 2697.80 | | 45.55  (34.42–57.59) | |  |
| Cameroon | 547.85 | 14.78  (7.6–22.45) | 535.59 | | 14.7  (7.51–22.37) | 570.93 | 18.03  (11.21–25.27) | | 566.33 | | 17.85  (10.95–25.17) | |  |
| Canada | 54.49 | 3.52  (0.96–6.15) | 51.56 | | 2.95  (0.19–5.79) | 24.04 | -1.01  (-4.13–2.21) | | 13.89 | | -1.51  (-4.57–1.65) | |  |
| Cape Verde | 648.05 | 18.68  (12.02–25.73) | 652.40 | | 18.9  (12.75–25.39) | 576.28 | 19.61  (13.54–26.01) | | 566.67 | | 19.48  (13.33–25.96) | |  |
| Central African Republic | 622.26 | 17.52  (10.36–25.14) | 608.33 | | 17.06  (9.82–24.77) | 637.45 | 20.36  (13.63–27.49) | | 655.04 | | 20.44  (13.68–27.6) | |  |
| Chad | 604.71 | 17.38  (11.03–24.09) | 597.03 | | 17.39  (11–24.14) | 569.66 | 19.4  (13.77–25.3) | | 589.14 | | 19.27  (13.68–25.12) | |  |
| Chile | 172.75 | 9.08  (6.3–11.93) | 186.96 | | 9.83  (7.73–11.96) | 121.18 | 6.25  (4.23–8.3) | | 84.33 | | 4.47  (2.46–6.53) | |  |
| China | 269.42 | 11.23  (3.94–19.02) | 322.14 | | 12.36  (6.37–18.68) | 114.12 | 4.25  (-1.24–10.04) | | 105.78 | | 4.94  (-0.74–10.95) | |  |
| Colombia | 1327.57 | 25.74  (17.29–34.81) | 1343.13 | | 25.87  (17.21–35.18) | 821.26 | 22.31  (14.67–30.45) | | 701.37 | | 21.34  (13.74–29.45) | |  |
| Comoros | 3338.10 | 39.07  (28.52–50.48) | 3397.49 | | 39.02  (28.54–50.35) | 3645.78 | 48.42  (40.31–56.99) | | 3202.96 | | 46.64  (38.36–55.41) | |  |
| Congo | 562.85 | 16.01  (8.87–23.63) | 539.02 | | 15.5  (8.46–23.01) | 537.63 | 18.69  (12.22–25.54) | | 554.52 | | 18.71  (12.21–25.6) | |  |
| Costa Rica | 4583.78 | 39  (26.15–53.17) | 4632.6 | | 39.06  (25.84–53.67) | 3233.36 | 43.46  (32.79–55) | | 3172.34 | | 43.81  (33.25–55.22) | |  |
| Cote d'Ivoire | 446.85 | 12.72  (4.67–21.39) | 439.31 | | 12.53  (4.58–21.08) | 478.72 | 15.5  (7.89–23.66) | | 453.76 | | 15.41  (7.74–23.63) | |  |
| Croatia | 49.58 | 4.35  (0.77–8.06) | 37.20 | | 3.34  (0.81–5.93) | -3.03 | -2.22  (-4.06–-0.34) | | -5.60 | | -2.18  (-4.22–-0.1) | |  |
| Cuba | 69.68 | 4.19  (0.68–7.83) | 68.13 | | 4.08  (0.55–7.74) | 22.24 | -0.29  (-6.08–5.84) | | 21.94 | | -0.05  (-5.55–5.77) | |  |
| Cyprus | 548.54 | 19.89  (16.6–23.27) | 530.72 | | 19.3  (16.09–22.6) | 364.86 | 15.45  (12.93–18.04) | | 343.16 | | 15.2  (12.61–17.85) | |  |
| Czech Republic | 91.45 | 6.12  (3.83–8.45) | 67.17 | | 4.77  (3.02–6.56) | -9.56 | -3.85  (-5.63–-2.03) | | -5.78 | | -3.15  (-4.83–-1.44) | |  |
| Democratic Republic  of Congo | 680.61 | 18.41  (10.89–26.43) | 692.22 | | 18.35  (10.76–26.46) | 684.91 | 21.54  (14.94–28.52) | | 662.92 | | 21.35  (14.73–28.36) | |  |
| Denmark | 124.12 | 9.25  (8.53–9.98) | 111.57 | | 7.94  (6.26–9.66) | 59.23 | 4.01  (1.55–6.53) | | 55.21 | | 3.63  (1.38–5.92) | |  |
| Djibouti | 9251.53 | 62.22  (56.24–68.42) | 9866.59 | | 62.6  (56.13–69.33) | 13630.11 | 73.64  (67.41–80.1) | | 12342.66 | | 72.26  (65.64–79.14) | |  |
| Dominica | 53.46 | 3.78  (-2.54–10.51) | 52.31 | | 3.7  (-2.74–10.57) | 9.68 | -0.71  (-6.8–5.77) | | 8.79 | | -0.56  (-6.59–5.86) | |  |
| Dominican Republic | 219.91 | 9.76  (5.18–14.53) | 231.02 | | 10.31  (5.84–14.97) | 115.69 | 5.1  (1.87–8.44) | | 82.27 | | 4.03  (0.77–7.4) | |  |
| Ecuador | 3126.76 | 34.56  (22.32–48.02) | 3011.87 | | 33.9  (21.27–47.85) | 1734.09 | 30.51  (19.91–42.04) | | 1644.93 | | 30.31  (19.82–41.72) | |  |
| Egypt | 2815.28 | 38.79  (31.27–46.74) | 2754.84 | | 38.4  (30.81–46.44) | 1636.52 | 35.98  (29.13–43.19) | | 1549.59 | | 35.29  (28.54–42.39) | |  |
| El Salvador | 1383.52 | 25.14  (14.07–37.29) | 1576.04 | | 27.37  (16.62–39.1) | 686.47 | 22.38  (13.11–32.42) | | 642.51 | | 22.57  (13.39–32.5) | |  |
| Equatorial Guinea | 491.88 | 13.17  (5.86–20.99) | 505.75 | | 14.11  (7–21.69) | 254.87 | 11.49  (3.86–19.68) | | 261.62 | | 10.74  (3.02–19.04) | |  |
| Eritrea | 3197.26 | 36.57  (24.38–49.96) | 3286.37 | | 37.7  (25.91–50.59) | 3285.83 | 45.61  (36.73–55.07) | | 3133.64 | | 44.88  (35.68–54.7) | |  |
| Estonia | 197.76 | 12.74  (9.98–15.58) | 217.30 | | 12.95  (9.34–16.68) | 239.37 | 14.98  (9.67–20.55) | | 245.43 | | 15.44  (9.94–21.22) | |  |
| Ethiopia | 4442.63 | 39.48  (27.04–53.15) | 4584.77 | | 40.35  (27.82–54.11) | 3487.71 | 47.81  (37.17–59.28) | | 3446.72 | | 47.46  (36.79–58.96) | |  |
| Federated States  of Micronesia | 417.40 | 14.92  (7.28–23.1) | 389.06 | | 14.1  (6.37–22.39) | 250.23 | 14.13  (7.47–21.2) | | 245.18 | | 13.8  (7.13–20.88) | |  |
| Fiji | 306.21 | 13.5  (8.93–18.26) | 309.31 | | 13.24  (8.62–18.06) | 306.55 | 16.08  (12.04–20.26) | | 294.66 | | 15.77  (11.85–19.82) | |  |
| Finland | 116.26 | 8.1  (7.38–8.83) | 120.40 | | 8.27  (8.08–8.47) | 57.03 | 1.40  (-0.11–2.93) | | 43.01 | | 0.77  (-0.67–2.22) | |  |
| France | 117.83 | 8.39  (7.48–9.3) | 123.41 | | 8.61  (8.29–8.92) | 148.20 | 7.09  (5.62–8.57) | | 124.25 | | 6.08  (4.66–7.52) | |  |
| Gabon | 583.81 | 16.95  (9.7–24.68) | 560.05 | | 16.39  (9.05–24.21) | 502.80 | 18.91  (12.62–25.55) | | 490.51 | | 18.54  (12.22–25.23) | |  |
| Georgia | 3937.53 | 48.44  (38.79–58.75) | 3870.85 | | 43.29  (29.72–58.28) | 2833.76 | 45.82  (32.81–60.11) | | 2659.61 | | 45.11  (32.3–59.15) | |  |
| Germany | 106.93 | 8.65  (8.31–9) | 81.65 | | 6.55  (5.88–7.23) | 28.99 | 0.64  (-0.82–2.11) | | 14.04 | | -0.76  (-2.2–0.72) | |  |
| Ghana | 452.73 | 13.9  (6.61–21.7) | 457.98 | | 13.95  (6.65–21.75) | 378.82 | 13.59  (7.15–20.41) | | 352.48 | | 13.3  (6.88–20.11) | |  |
| Greece | 185.52 | 10.12  (6.86–13.47) | 184.38 | | 9.92  (6.76–13.17) | 66.25 | 0.91  (-2.21–4.12) | | 65.94 | | 1.49  (-1.57–4.65) | |  |
| Greenland | -10.33 | -1.27  (-2.96–0.45) | -7.78 | | -1.14  (-2.85–0.6) | -21.24 | -5.67  (-7.60–-3.69) | | -29.63 | | -6.18  (-8.08–-4.23) | |  |
| Grenada | 158.75 | 7.19  (3.12–11.43) | 150.17 | | 6.99  (2.89–11.24) | 33.11 | 0.27  (-3.82–4.53) | | 41.37 | | 0.27  (-3.74–4.45) | |  |
| Guam | 722.94 | 21.3  (15.71–27.17) | 711.11 | | 20.48  (15.06–26.16) | 544.46 | 18.03  (12.41–23.92) | | 522.69 | | 18.36  (12.84–24.14) | |  |
| Guatemala | 2292.12 | 28.1  (14.35–43.5) | 2346.75 | | 28.79  (14.45–44.92) | 1077.32 | 25.41  (12.9–39.31) | | 1000.83 | | 25.38  (13.03–39.07) | |  |
| Guinea | 511.17 | 15.69  (9.01–22.79) | 538.31 | | 16.24  (9.52–23.37) | 471.22 | 17.01  (10.77–23.60) | | 408.79 | | 16.02  (9.78–22.61) | |  |
| Guinea-Bissau | 452.13 | 15.33  (9.23–21.76) | 470.07 | | 15.98  (9.91–22.39) | 376.45 | 15.64  (9.71–21.89) | | 364.51 | | 15.06  (9.12–21.33) | |  |
| Guyana | 290.70 | 16  (14.63–17.39) | 325.41 | | 17.08  (15.22–18.98) | 270.55 | 13.44  (10.65–16.3) | | 272.42 | | 14.43  (11.75–17.17) | |  |
| Haiti | 135.65 | 6  (2–10.15) | 146.65 | | 6.69  (2.55–11) | 66.75 | 1.76  (-2.45–6.16) | | 61.35 | | 1.49  (-2.73–5.91) | |  |
| Honduras | 3399.83 | 35.57  (22.86–49.59) | 3507.38 | | 36.23  (23.18–50.66) | 2095.76 | 38.58  (28.32–49.67) | | 1756.16 | | 36.38  (26.29–47.26) | |  |
| Hungary | 664.94 | 21.55  (14.89–28.6) | 513.26 | | 17.92  (10.18–26.21) | 241.87 | 11.4  (4.19–19.1) | | 245.04 | | 11.62  (4.22–19.54) | |  |
| Iceland | 483.28 | 19.82  (16.89–22.82) | 453.95 | | 18.6  (15.33–21.95) | 335.31 | 15.43  (12.37–18.58) | | 307.83 | | 14.74  (11.91–17.64) | |  |
| India | 4757.27 | 43.5  (32.37–55.57) | 4814.09 | | 43.35  (32.09–55.56) | 3457.42 | 44.87  (35.52–54.87) | | 3367.91 | | 44.75  (35.46–54.69) | |  |
| Indonesia | 666.35 | 17.83  (7.54–29.1) | 819.58 | | 19.33  (8.59–31.13) | 731.32 | 23.06  (13.98–32.87) | | 630.79 | | 21.93  (12.92–31.65) | |  |
| Iran | 2340.98 | 38.15  (31.87–44.73) | 2291.62 | | 37.44  (31.21–43.98) | 1407.42 | 34.33  (29.21–39.65) | | 1169.21 | | 33.58  (28.33–39.04) | |  |
| Iraq | 4173.71 | 44.78  (39.77–49.97) | 4234.95 | | 45.06  (40.03–50.28) | 3107.88 | 43.9  (38.24–49.8) | | 3209.61 | | 44.03  (38.32–49.98) | |  |
| Ireland | 130.53 | 7.6  (3.87–11.46) | 130.78 | | 6.93  (1.63–12.51) | 58.25 | 3.07  (0.87–5.32) | | 53.54 | | 2.8  (0.61–5.04) | |  |
| Israel | 377.08 | 13.55  (8.34–19.01) | 424.75 | | 14.35  (8.61–20.4) | 403.14 | 14.76  (9.43–20.34) | | 359.70 | | 14.3  (9.08–19.78) | |  |
| Italy | 365.01 | 19.35  (17.67–21.06) | 358.45 | | 18.62  (18.15–19.1) | 307.05 | 11.86  (9.51–14.26) | | 256.02 | | 11.08  (8.84–13.36) | |  |
| Jamaica | 216.04 | 9.67  (3.63–16.07) | 232.72 | | 10.3  (4.26–16.69) | 189.34 | 10.04  (4.33–16.05) | | 193.99 | | 10.62  (5.01–16.53) | |  |
| Japan | 266.15 | 10.4  (4.27–16.89) | 243.58 | | 9.92  (4.08–16.1) | 309.29 | 11.66  (6.66–16.9) | | 247.71 | | 10.51  (5.48–15.77) | |  |
| Jordan | 2755.30 | 34.42  (24.34–45.32) | 2761.35 | | 34.49  (24.43–45.36) | 1901.15 | 36.37  (27.73–45.59) | | 1828.57 | | 35.66  (27.14–44.76) | |  |
| Kazakhstan | 5737.25 | 46.41  (30.32–64.49) | 6829.97 | | 48.11  (30.99–67.47) | 7719.93 | 62.58  (45.31–81.9) | | 7830.73 | | 63.48  (46.09–82.94) | |  |
| Kenya | 8339.85 | 58.07  (50.79–65.71) | 8326.56 | | 57.87  (50.27–65.86) | 9067.39 | 67.16  (60.73–73.85) | | 7886.26 | | 65.39  (58.81–72.24) | |  |
| Kiribati | 480.50 | 15.44  (7.57–23.89) | 500.99 | | 15.92  (8.11–24.31) | 442.23 | 18.55  (11.3–26.28) | | 440.12 | | 18.59  (11.41–26.24) | |  |
| Kuwait | 1511.46 | 30.55  (23.1–38.44) | 1497.51 | | 29.88  (22–38.26) | 1412.53 | 32.32  (26.18–38.76) | | 1231.96 | | 32.58  (26.15–39.35) | |  |
| Kyrgyzstan | 12126.55 | 63.94  (51.99–76.84) | 14528.84 | | 65.68  (52.13–80.44) | 14544.53 | 76.33  (62.45–91.39) | | 13633.70 | | 75.79  (62.66–89.98) | |  |
| Laos | 1103.01 | 20.96  (9.28–33.88) | 1191.55 | | 21.97  (10.15–35.07) | 917.70 | 26.14  (16.2–36.94) | | 879.19 | | 25.61  (15.71–36.35) | |  |
| Latvia | 12.11 | 1.66  (-1.78–5.22) | 17.33 | | 1.9  (-2.26–6.23) | 40.74 | 3.66  (-2.53–10.26) | | 41.88 | | 3.88  (-2.63–10.82) | |  |
| Lebanon | 1636.50 | 28.07  (19.39–37.39) | 1587.94 | | 27.62  (18.98–36.9) | 912.04 | 25.84  (18.73–33.39) | | 895.70 | | 25.67  (18.52–33.25) | |  |
| Lesotho | 3751.26 | 46.48  (40.27–52.97) | 3938.92 | | 46.99  (40.78–53.48) | 3952.72 | 48.54  (43–54.3) | | 3829.86 | | 48.49  (43.06–54.12) | |  |
| Liberia | 536.31 | 14.08  (5.32–23.57) | 593.96 | | 15.62  (7.34–24.54) | 478.34 | 19.9  (12.45–27.84) | | 440.42 | | 18.78  (11.42–26.61) | |  |
| Libya | 1550.63 | 32.77  (29.01–36.63) | 1563.13 | | 32.81  (29.11–36.61) | 952.77 | 26.62  (23.5–29.83) | | 910.94 | | 26.71  (23.6–29.91) | |  |
| Lithuania | 203.05 | 11.98  (9.26–14.78) | 217.35 | | 11.89  (8.08–15.85) | 223.73 | 11.54  (5.41–18.03) | | 218.74 | | 11.5  (5.12–18.27) | |  |
| Luxembourg | 179.79 | 10.22  (7.08–13.44) | 173.15 | | 9.56  (6.02–13.22) | 101.04 | 5.97  (3.06–8.96) | | 90.79 | | 5.49  (2.49–8.57) | |  |
| Macedonia | 528.04 | 16.98  (9.52–24.95) | 501.60 | | 16.24  (8.77–24.23) | 425.62 | 16.46  (9.31–24.08) | | 370.37 | | 15.24  (8.16–22.79) | |  |
| Madagascar | 3424.80 | 38.08  (27.36–49.7) | 3521.86 | | 38.74  (28.23–50.11) | 3283.04 | 48.96  (40.05–58.43) | | 3199.19 | | 47.94  (38.93–57.55) | |  |
| Malawi | 6412.32 | 55.5  (47.71–63.71) | 6170.33 | | 53.95  (46.17–62.13) | 6807.07 | 65.13  (57.46–73.18) | | 5837.63 | | 63.27  (55.22–71.74) | |  |
| Malaysia | 3398.39 | 32.66  (18.06–49.06) | 4093.04 | | 36.36  (22.69–51.55) | 3040.66 | 45.57  (33.66–58.54) | | 2991.41 | | 44.27  (32.66–56.9) | |  |
| Maldives | 808.11 | 16.92  (5.35–29.75) | 856.44 | | 17.46  (5.86–30.34) | 444.32 | 16.79  (6.95–27.53) | | 362.24 | | 15.51  (5.8–26.12) | |  |
| Mali | 397.31 | 13.13  (6.42–20.27) | 410.40 | | 13.92  (7.17–21.09) | 239.22 | 11.15(  4.91–17.77) | | 241.64 | | 10.66  (4.39–17.31) | |  |
| Malta | 333.89 | 16.26  (13.84–18.74) | 345.75 | | 16.2  (13.65–18.81) | 222.34 | 10.6  (8–13.26) | | 212.63 | | 10.81  (8.23–13.45) | |  |
| Marshall Islands | 2025.74 | 31.95  (22.4–42.24) | 2032.75 | | 31.91  (22.31–42.25) | 1702.90 | 36.63  (28.37–45.43) | | 1786.93 | | 36.6  (28.43–45.29) | |  |
| Mauritania | 594.32 | 18.58  (13.2–24.21) | 587.48 | | 18.5  (13.05–24.21) | 384.57 | 15.2  (9.49–21.22) | | 397.96 | | 14.82  (9.11–20.82) | |  |
| Mauritius | 1332.75 | 26.09  (15.71–37.4) | 1604.36 | | 27.48  (16.62–39.35) | 782.89 | 25.57  (16.72–35.1) | | 823.25 | | 25.43  (16.61–34.93) | |  |
| Mexico | 5824.11 | 42.95  (29.29–58.05) | 5556.99 | | 42.88  (28.71–58.61) | 2404.54 | 41.45  (32.38–51.13) | | 2300.50 | | 41.28  (32.13–51.06) | |  |
| Moldova | 1544.44 | 27.74  (15.05–41.83) | 1935.16 | | 30.14  (16.68–45.16) | 2358.78 | 38.49  (25.28–53.08) | | 2227.91 | | 37.73  (24.77–52.03) | |  |
| Mongolia | 1200.08 | 25.25  (16.12–35.09) | 1194.28 | | 25.32  (16.26–35.08) | 941.74 | 28.29  (19.96–37.2) | | 830.03 | | 27.7  (19.25–36.73) | |  |
| Montenegro | 546.32 | 20.34  (15.84–25) | 523.36 | | 19.52  (14.94–24.27) | 583.81 | 20.33  (16.01–24.82) | | 555.73 | | 20.18  (15.87–24.66) | |  |
| Morocco | 2793.59 | 41.81  (36.59–47.21) | 2833.49 | | 42.06  (36.83–47.5) | 2058.65 | 40.46  (35.62–45.48) | | 1819.85 | | 39.28  (34.46–44.26) | |  |
| Mozambique | 1511.54 | 27.2  (17.3–37.93) | 1629.05 | | 28.75  (18.67–39.68) | 1652.32 | 34.67  (25.51–44.50) | | 1444.97 | | 32.78  (23.53–42.71) | |  |
| Myanmar | 2277.43 | 31.45  (18.91–45.32) | 2497.44 | | 32.44  (19.77–46.45) | 2133.78 | 37.46  (26.26–49.65) | | 2001.15 | | 36.87  (25.73–49.01) | |  |
| Namibia | 4028.72 | 44.99  (38.72–51.54) | 4203.20 | | 45.76  (39.24–52.59) | 4049.31 | 46.72  (40.71–52.99) | | 3853.46 | | 46.47  (40.61–52.57) | |  |
| Nepal | 507.56 | 19.62  (18.69–20.57) | 522.67 | | 19.87  (18.81–20.94) | 347.38 | 14.34  (12.46–16.26) | | 309.36 | | 13.52  (11.67–15.4) | |  |
| Netherlands | 49.13 | 3.95  (1.16–6.81) | 45.14 | | 3.3  (0.64–6.02) | 34.90 | 0.3  (-2.63–3.32) | | 25.74 | | -0.16  (-2.87–2.61) | |  |
| New Zealand | 22.62 | 1.35  (-0.21–2.94) | 28.61 | | 1.57  (-0.36–3.53) | 21.97 | -0.34  (-2.45–1.81) | | 12.16 | | -0.92  (-2.96–1.17) | |  |
| Nicaragua | 797.77 | 19.03  (10.61–28.1) | 844.32 | | 19.99  (11.57–29.03) | 563.56 | 19.7  (12.34–27.54) | | 467.30 | | 18.72  (11.56–26.35) | |  |
| Niger | 545.01 | 15.21  (7.99–22.91) | 561.84 | | 15.62  (8.38–23.34) | 406.28 | 15.76  (9.12–22.82) | | 369.65 | | 14.55  (7.89–21.61) | |  |
| Nigeria | 3900.83 | 38.93  (27.87–50.94) | 3802.81 | | 39.04  (28.07–50.94) | 3129.47 | 43.55  (33.39–54.48) | | 3151.28 | | 42.97  (32.85–53.85) | |  |
| North Korea | 7834.70 | 47.92  (31.24–66.72) | 8057.11 | | 48.14  (32–66.26) | 8027.46 | 64.53  (50.92–79.36) | | 7970.22 | | 63.48  (50.11–78.03) | |  |
| Northern Mariana Islands | 1140.89 | 23.86  (19.32–28.58) | 1007.39 | | 21.84  (17.3–26.55) | 545.33 | 17.13  (13.31–21.08) | | 580.19 | | 17.3  (13.51–21.21) | |  |
| Norway | 144.24 | 9.17  (5.37–13.11) | 117.51 | | 7.38  (2.86–12.09) | 77.95 | 4.83  (1.09–8.71) | | 60.73 | | 4.12  (0.43–7.95) | |  |
| Oman | 504.60 | 15.5  (7.86–23.67) | 514.00 | | 15.66  (8–23.86) | 243.40 | 11.71  (5.64–18.13) | | 204.95 | | 10.76  (4.76–17.1) | |  |
| Pakistan | 6838.15 | 49.71  (37.99–62.41) | 6843.18 | | 50.06  (38.36–62.74) | 6091.48 | 57.78  (47.42–68.86) | | 6166.06 | | 57.44  (46.98–68.66) | |  |
| Palestine | 1133.24 | 24.71  (19.52–30.11) | 1170.03 | | 25.02  (19.76–30.52) | 659.94 | 22.74  (18.31–27.33) | | 626.78 | | 22.01  (17.64–26.53) | |  |
| Panama | 1863.24 | 28.89  (18.41–40.29) | 1960.14 | | 29.63  (18.89–41.34) | 1253.22 | 28.17  (18.65–38.45) | | 1133.58 | | 27.78  (18.32–37.99) | |  |
| Papua New Guinea | 1865.96 | 31.14  (24.04–38.64) | 1803.28 | | 31.03  (23.78–38.71) | 1356.31 | 33.27  (26.9–39.96) | | 1400.85 | | 33.14  (26.94–39.64) | |  |
| Paraguay | 2776.29 | 36.11  (27.25–45.58) | 2919.42 | | 37.02  (28.25–46.38) | 1893.09 | 33.08  (24.97–41.71) | | 1782.08 | | 33.1  (24.82–41.93) | |  |
| Peru | 718.21 | 22.35  (18.89–25.91) | 752.53 | | 23.22  (19.94–26.6) | 331.94 | 16.31  (12.22–20.56) | | 279.99 | | 15.35  (11.45–19.39) | |  |
| Philippines | 5364.31 | 43.74  (31.41–57.22) | 5763.42 | | 44.38  (31.51–58.52) | 4348.21 | 52.3  (41.58–63.84) | | 4072.14 | | 51.45  (41.02–62.66) | |  |
| Poland | 327.20 | 14.07  (8.9–19.49) | 253.73 | | 12.26  (7.93–16.77) | 147.68 | 7.37  (3.3–11.61) | | 144.89 | | 7.19  (3.03–11.51) | |  |
| Portugal | 157.80 | 8.37  (3.8–13.14) | 160.51 | | 8.52  (4.03–13.2) | 132.98 | 7.49  (4.13–10.97) | | 120.86 | | 7.34  (3.94–10.85) | |  |
| Puerto Rico | 804.34 | 21.5  (13.75–29.77) | 844.79 | | 21.24  (12.64–30.49) | 507.97 | 17.52  (9.37–26.28) | | 479.32 | | 17.59  (9.36–26.43) | |  |
| Qatar | 396.44 | 12.61  (5.11–20.65) | 398.83 | | 12.63  (5.14–20.66) | 231.24 | 11.4  (3.3–20.14) | | 197.74 | | 9.83  (1.76–18.54) | |  |
| Romania | 378.21 | 14.29  (5.5–23.81) | 348.20 | | 13.4  (4.46–23.12) | 306.41 | 15.74  (6.42–25.89) | | 276.99 | | 15.14  (5.86–25.22) | |  |
| Russian Federation | 1926.75 | 32.39  (22.18–43.45) | 2038.25 | | 32.94  (22.23–44.6) | 1838.12 | 37.01  (25.31–49.8) | | 1976.96 | | 38.04  (26.21–50.98) | |  |
| Rwanda | 6267.77 | 55.16  (47.73–62.96) | 6310.95 | | 55.76  (48.26–63.64) | 6051.23 | 62.08  (53.48–71.15) | | 6580.92 | | 62.79  (53.73–72.38) | |  |
| Saint Lucia | 139.38 | 6.27  (0.44–12.44) | 127.45 | | 5.83  (-0.15–12.18) | 53.58 | 1.73  (-3.72–7.49) | | 51.85 | | 1.98  (-3.49–7.76) | |  |
| Saint Vincent and  the Grenadines | 44.70 | 2.57  (-3.37–8.86) | 41.81 | | 2.37  (-3.73–8.86) | 0.49 | -2.61  (-8.16–3.27) | | 1.11 | | -2.32  (-7.91–3.6) | |  |
| Samoa | 674.15 | 20.81  (13.96–28.07) | 703.93 | | 21.06  (14.23–28.3) | 545.14 | 23.41  (17.31–29.84) | | 504.50 | | 22.59  (16.58–28.91) | |  |
| Sao Tome and Principe | 592.96 | 19.32  (12.77–26.24) | 608.67 | | 19.55  (13–26.49) | 566.09 | 21.6  (15.44–28.09) | | 534.38 | | 21.04  (14.77–27.66) | |  |
| Saudi Arabia | 4309.80 | 46.41  (40.14–52.97) | 4474.39 | | 46.71  (40.32–53.4) | 2536.62 | 42.69  (37–48.62) | | 2562.67 | | 42.12  (36.48–47.98) | |  |
| Senegal | 3057.96 | 36.29  (25.94–47.48) | 3123.97 | | 36.49  (26.19–47.63) | 2413.16 | 39.31  (29.94–49.36) | | 2277.14 | | 38.89  (29.53–48.92) | |  |
| Serbia | 290.24 | 13.12  (6.97–19.62) | 289.85 | | 12.75  (6.46–19.4) | 295.25 | 13.73  (7.44–20.39) | | 270.25 | | 13.47  (7.2–20.11) | |  |
| Seychelles | 1618.95 | 27.54  (16.32–39.84) | 1857.21 | | 28.66  (17.02–41.44) | 1382.76 | 36.2  (26.05–47.16) | | 1425.34 | | 35.6  (25.56–46.45) | |  |
| Sierra Leone | 289.37 | 14.61  (11.46–17.86) | 332.52 | | 16.13  (13.59–18.73) | 338.56 | 17.63  (15.19–20.11) | | 291.60 | | 16.68  (14.23–19.19) | |  |
| Singapore | 81.33 | 3.79  (0.67–7) | 83.06 | | 3.67  (1.03–6.38) | -14.08 | -7.26  (-10.13–-4.3) | | 8.63 | | -3.7  (-6.46–-0.86) | |  |
| Slovakia | 106.82 | 6.99  (4.77–9.25) | 92.98 | | 6.26  (4.49–8.06) | 36.45 | 0.63  (-1.41–2.72) | | 36.61 | | 0.67  (-1.26–2.64) | |  |
| Slovenia | 152.49 | 8.27  (3.08–13.71) | 119.78 | | 6.86  (2.47–11.45) | 43.44 | 1.65  (-2.08–5.53) | | 39.56 | | 1.6  (-2.28–5.64) | |  |
| Solomon Islands | 506.45 | 14.35  (6.87–22.35) | 521.46 | | 14.62  (7.11–22.65) | 360.68 | 14.11  (7.54–21.08) | | 358.69 | | 14.02  (7.53–20.9) | |  |
| Somalia | 13892.06 | 66.08  (55.93–76.89) | 14721.83 | | 67.92  (58.28–78.16) | 18580.63 | 81.08  (72.54–90.05) | | 17411.63 | | 79.93  (70.87–89.47) | |  |
| South Africa | 684.32 | 23.11  (22.62–23.61) | 623.37 | | 21.79  (21.08–22.49) | 666.18 | 19.76  (16.8–22.79) | | 586.30 | | 17.9  (14.79–21.09) | |  |
| South Korea | 249.70 | 11.09  (5.52–16.94) | 278.93 | | 11.87  (6.73–17.26) | 280.14 | 12.11  (8.36–16) | | 191.36 | | 8.8  (5.06–12.68) | |  |
| South Sudan | 4089.65 | 42.53  (31.47–54.52) | 4294.68 | | 43.1  (31.87–55.29) | 5051.68 | 55.24  (45.22–65.96) | | 4637.46 | | 53.39  (43.09–64.42) | |  |
| Spain | 144.12 | 9.22  (6.85–11.64) | 113.82 | | 7.17  (4.44–9.96) | 94.49 | 3.7  (1.07–6.4) | | 61.85 | | 2.21  (-0.45–4.94) | |  |
| Sri Lanka | 881.12 | 19.33  (8.13–31.68) | 940.42 | | 19.59  (8.2–32.19) | 654.52 | 24.91  (15.87–34.66) | | 661.89 | | 25.11  (16.03–34.91) | |  |
| Sudan | 1740.92 | 30.96  (24.31–37.98) | 1765.14 | | 31.34  (24.68–38.36) | 1047.86 | 29.46  (23.69–35.49) | | 995.60 | | 28.88  (23.08–34.96) | |  |
| Suriname | 78.06 | 4  (-1.51–9.82) | 83.12 | | 4.4  (-1.07–10.17) | 44.63 | 0.68  (-4.40–6.02) | | 38.45 | | 0.7  (-4.56–6.25) | |  |
| Swaziland | 388.71 | 14.15  (13.31–14.99) | 367.28 | | 13.72  (13.19–14.25) | 507.58 | 16.28  (14.33–18.26) | | 489.82 | | 16.53  (14.63–18.46) | |  |
| Sweden | 137.65 | 9.93  (8.29–11.59) | 111.89 | | 7.9  (5.4–10.45) | 96.67 | 4.45  (2.67–6.27) | | 75.39 | | 3.55  (1.83–5.3) | |  |
| Switzerland | 206.41 | 13.19  (12.89–13.48) | 191.28 | | 12.12  (11.39–12.86) | 134.34 | 6.93  (5.89–7.98) | | 102.82 | | 5.54  (4.33–6.77) | |  |
| Syria | 3065.07 | 35.45  (24.39–47.49) | 3035.45 | | 35.04  (23.91–47.17) | 1563.74 | 35.12  (25.41–45.57) | | 1391.98 | | 33.37  (23.88–43.59) | |  |
| Taiwan  (Province of China) | 484.83 | 13.73  (2.73–25.91) | 554.65 | | 14.53  (3.95–26.19) | 365.13 | 13.1  (3.87–23.14) | | 306.80 | | 11.89  (2.83–21.76) | |  |
| Tajikistan | 12221.33 | 61.1  (46.44–77.23) | 12192.71 | | 60.79  (46.19–76.85) | 11176.07 | 73.21  (59.34–88.29) | | 10122.52 | | 72.2  (58.48–87.11) | |  |
| Tanzania | 4489.87 | 42.97  (33.02–53.67) | 4266.08 | | 41.94  (31.86–52.79) | 5362.75 | 56.72  (48.09–65.86) | | 4853.01 | | 55.04  (46.15–64.47) | |  |
| Thailand | 158.38 | 6.5  (-1.31–14.93) | 185.91 | | 6.85  (-1.03–15.36) | 93.58 | 2.26  (-4.67–9.68) | | 93.93 | | 3.21  (-3.85–10.79) | |  |
| The Bahamas | 786.89 | 20.87  (13.96–28.2) | 811.65 | | 20.98  (13.52–28.93) | 560.87 | 18.00  (10.33–26.21) | | 538.21 | | 17.84  (10.44–25.74) | |  |
| The Gambia | 484.83 | 14.43  (7.7–21.58) | 506.38 | | 14.94  (8.35–21.93) | 445.11 | 15.65  (9.74–21.87) | | 383.93 | | 14.72  (8.81–20.96) | |  |
| Timor-Lester | 969.85 | 20.84  (9.59–33.24) | 1092.08 | | 21.97  (10.57–34.56) | 737.56 | 23.84  (14.27–34.21) | | 624.54 | | 22.47  (12.93–32.82) | |  |
| Togo | 504.54 | 14.94  (7.96–22.37) | 512.96 | | 15.03  (8.14–22.35) | 468.01 | 16.56  (10.15–23.34) | | 438.05 | | 16.07  (9.65–22.87) | |  |
| Tonga | 404.39 | 14.53  (6.71–22.93) | 406.09 | | 14.27  (6.3–22.83) | 339.29 | 15.56  (8.43–23.17) | | 322.70 | | 15.44  (8.39–22.94) | |  |
| Trinidad and Tobago | 160.86 | 8.11  (3.33–13.1) | 180.31 | | 8.57  (3.17–14.24) | 142.34 | 5.68  (-0.16–11.87) | | 136.12 | | 5.97  (0.18–12.1) | |  |
| Tunisia | 2259.30 | 36.58  (30.2–43.28) | 2328.53 | | 36.96  (30.58–43.64) | 1562.03 | 35.01  (29.73–40.49) | | 1248.92 | | 33.85  (28.51–39.41) | |  |
| Turkey | 4087.30 | 47.01  (39.84–54.55) | 4011.33 | | 46.23  (38.87–53.99) | 2588.42 | 45.24  (38.46–52.34) | | 2260.48 | | 44.79  (38.07–51.83) | |  |
| Turkmenistan | 13623.38 | 66.78  (55.5–78.89) | 14456.67 | | 66.13  (53.53–79.76) | 13477.71 | 75.07  (62.32–88.81) | | 12613.14 | | 75.4  (63–88.75) | |  |
| Uganda | 8643.27 | 58.49  (49.84–67.63) | 8796.97 | | 59.13  (50.86–67.84) | 10500.52 | 70.99  (63.45–78.88) | | 9282.32 | | 69.17  (61.42–77.29) | |  |
| Ukraine | 2604.40 | 36.23  (24.14–49.48) | 3108.03 | | 37.55  (23.58–53.09) | 2498.13 | 40.89  (27.36–55.87) | | 2863.05 | | 42.9  (29.16–58.11) | |  |
| United Arab Emirates | 1649.72 | 26.18  (19.7–33.02) | 1677.50 | | 26.68  (20.2–33.51) | 1032.69 | 25.16  (20.61–29.87) | | 1005.58 | | 24.54  (19.89–29.37) | |  |
| United Kingdom | 9.95 | 1.13  (-1.81–4.15) | -0.96 | | 0.04  (-3.27–3.46) | -18.46 | -4.11  (-7.46–-0.64) | | -20.94 | | -3.96  (-7.23–-0.58) | |  |
| United States | -41.51 | -6.78  (-8.19–-5.36) | -61.00 | | -10.84  (-11.4–-10.28) | -70.99 | -16.72  (-19.41–-13.94) | | -73.45 | | -17.26  (-19.91–-14.54) | |  |
| Uruguay | 328.35 | 16.65  (15.16–18.15) | 321.67 | | 16.2  (14.25–18.19) | 184.41 | 9.83  (7.73–11.96) | | 188.70 | | 11.01  (9.14–12.9) | |  |
| Uzbekistan | 11081.21 | 56.4  (41.34–73.07) | 15288.57 | | 64.11  (50.06–79.47) | 14297.73 | 73.14  (60.84–86.39) | | 13844.77 | | 73.89  (61.4–87.36) | |  |
| Vanuatu | 325.68 | 12.28  (7.7–17.06) | 315.20 | | 12.1  (7.63–16.77) | 278.75 | 13.83  (9.7–18.13) | | 283.41 | | 13.64  (9.57–17.85) | |  |
| Venezuela | 1551.62 | 26.58  (17.11–36.82) | 1574.42 | | 26.9  (17.21–37.39) | 937.23 | 24.23  (16.28–32.73) | | 854.64 | | 24.14  (16.33–32.48) | |  |
| Vietnam | 600.45 | 18.05  (11.06–25.48) | 686.00 | | 19  (11.95–26.49) | 427.71 | 15.09  (8.44–22.15) | | 386.97 | | 14.4  (7.76–21.45) | |  |
| Virgin Islands, U.S. | 163.20 | 8.53  (3.46–13.84) | 170.09 | | 8.61  (3.49–13.98) | 92.95 | 3.69  (-0.75–8.32) | | 75.42 | | 3.52  (-0.82–8.05) | |  |
| Yemen | 2760.41 | 35.35  (26.19–45.18) | 2836.6 | | 35.95  (26.71–45.86) | 1620.15 | 35.13  (27.57–43.14) | | 1506.22 | | 34.48  (26.88–42.53) | |  |
| Zambia | 2298.64 | 34.39  (24.43–45.16) | 2157.87 | | 32.55  (22.89–42.97) | 2922.13 | 44.44  (34.66–54.93) | | 2501.84 | | 42.32  (32.49–52.88) | |  |
| Zimbabwe | 693.50 | 22.46  (18.51–26.53) | 678.89 | | 21.45  (17.48–25.56) | 873.63 | 24.07  (21.93–26.24) | | 834.30 | | 24.67  (22.66–26.72) | |  |

MDR-TB: multidrug-resistant tuberculosis; DALYs: disability-adjusted life-years; EAPC: estimated annual percentage change; ASR, age-standardized rate; CI, confidence interval; UI: uncertainty interval. Percentage change in absolute number was calculated based on the crew data.

**Table S5**. the percentage change in absolute number and EAPCs of MDR-TB at national level and both sexes from 2000 to 2017

| **Characteristics** | **Incidence** | | **Prevalence** | | | **Death** | | | | | **DALYs** | | | |
| --- | --- | --- | --- | --- | --- | --- | --- | --- | --- | --- | --- | --- | --- | --- |
|  | Change in  number (%) | EAPC  (95%CI) | Change in number (%) | EAPC  (95%CI) | | Change in  number (%) | | EAPC  (95%CI) | | Change in  number (%) | | EAPC  (95%CI) | | |
| Afghanistan | 93.39 | 2.00  (0.5–3.53) | 98.67 | | 2.30  (0.74–3.88) | -15.15 | -3.10  (-4.46–-1.72) | | -17.78 | | | | -3.55  (-4.92–-2.16) |  |
| Albania | -31.71 | -1.69  (-3.05–-0.31) | -19.71 | | -1.03  (-2.42–0.37) | -60.78 | -8.53  (-9.34–-7.72) | | -67.19 | | | | -8.02  (-8.75–-7.3) |  |
| Algeria | -5.43 | -2.27  (-3–-1.53) | -5.34 | | -2.31  (-3.02–-1.6) | -34.16 | -5.27  (-5.88–-4.66) | | -47.68 | | | | -5.77  (-6.45–-5.08) |  |
| American Samoa | -53.31 | -6.1  (-7.36–-4.83) | -28.21 | | -3.40  (-4.3–-2.49) | -46.85 | -5.10  (-6.34–-3.85) | | -49.89 | | | | -4.98  (-6.19–-3.75) |  |
| Andorra | -18.58 | -2.64  (-3.59–-1.68) | -7.92 | | -2.39  (-3.32–-1.46) | -24.37 | -5.00  (-5.79–-4.2) | | -32.40 | | | | -5.06  (-5.85–-4.26) |  |
| Angola | 92.08 | -0.55  (-1.06–-0.05) | 103.71 | | -0.19  (-0.76–0.38) | 8.04 | -3.25  (-3.97–-2.51) | | -6.63 | | | | -3.7  (-4.44–-2.96) |  |
| Antigua and Barbuda | -61.08 | -8.37  (-10.71–-5.96) | -62.65 | | -8.99  (-11.56–-6.35) | -80.71 | -11.64  (-14.25–-8.96) | | -80.74 | | | | -11.24  (-13.83–-8.57) |  |
| Argentina | -46.33 | -6.61  (-7.78–-5.43) | -48.16 | | -6.74  (-7.9–-5.57) | -64.22 | -9.13  (-10.22–-8.02) | | -65.59 | | | | -9.01  (-10.03–-7.98) |  |
| Armenia | 60.89 | 2.93  (0.58–5.33) | 70.92 | | 2.74  (0.87–4.65) | -16.96 | -2.78  (-5.35–-0.13) | | -26.30 | | | | -3.21  (-5.76–-0.6) |  |
| Australia | 411.40 | 9.15  (8.66–9.65) | 402.48 | | 8.81  (8.5–9.11) | 277.08 | 5.57  (4.99–6.15) | | 245.52 | | | | 5.79  (5.23–6.35) |  |
| Austria | 240.38 | 7.15  (4.33–10.04) | 276.06 | | 7.57  (4.69–10.53) | 188.02 | 4.82  (2.33–7.38) | | 153.31 | | | | 4.55  (2.06–7.1) |  |
| Azerbaijan | 331.53 | 5.36  (3.55–7.19) | 241.58 | | 4.4  (2.84–5.99) | 34.28 | -1.38  (-2.83–0.1) | | 24.93 | | | | -1.55  (-2.95–-0.12) |  |
| Bahrain | 75.04 | -2.84  (-3.64–-2.02) | 70.62 | | -2.81  (-3.66–-1.96) | -0.99 | -6.04  (-7.04–-5.02) | | -5.78 | | | | -6.03  (-7.08–-4.97) |  |
| Bangladesh | 31.21 | -1.42  (-2.34–-0.49) | 51.71 | | -1.12  (-2.02–-0.2) | -37.28 | -6.36  (-7.49–-5.21) | | -50.38 | | | | -7.01  (-8.02–-5.98) |  |
| Barbados | -82.23 | -12.32  (-15.31–-9.23) | -81.78 | | -12.51  (-15.49–-9.42) | -87.46 | -13.88  (-16.99–-10.66) | | -88.24 | | | | -13.86  (-16.91–-10.69) |  |
| Belarus | 26.77 | 1.69  (0.62–2.78) | 49.89 | | 2.58  (1.18–4) | -35.81 | -4.05  (-5.85–-2.21) | | -37.88 | | | | -4.06  (-5.94–-2.14) |  |
| Belgium | -23.90 | -1.27  (-1.71–-0.83) | -23.54 | | -1.37  (-1.74–-0.99) | -50.13 | -5.72  (-6.28–-5.15) | | -56.63 | | | | -5.81  (-6.35–-5.27) |  |
| Belize | -38.18 | -8.02  (-10.01–-5.98) | -42.88 | | -8.58  (-10.63–-6.47) | -60.65 | -10.2  (-12.52–-7.81) | | -63.02 | | | | -10.12  (-12.4–-7.78) |  |
| Benin | 56.27 | -2.19  (-3–-1.37) | 66.17 | | -1.89  (-2.73–-1.05) | 12.03 | -3.67  (-4.46–-2.86) | | 4.18 | | | | -3.88  (-4.69–-3.06) |  |
| Bermuda | -63.51 | -9.09  (-12–-6.08) | -58.97 | | -9.06  (-11.94–-6.08) | -81.22 | -12.53  (-15.8–-9.13) | | -81.31 | | | | -11.8  (-14.9–-8.58) |  |
| Bhutan | 61.54 | -0.56  (-0.82–-0.31) | 75.67 | | -0.23  (-0.51–0.04) | -31.19 | -5.43  (-5.78–-5.07) | | -39.62 | | | | -6.02  (-6.38–-5.66) |  |
| Bolivia | 3.91 | -2.93  (-4.43–-1.41) | 16.84 | | -2.23  (-3.63–-0.81) | -39.88 | -5.33  (-6.76–-3.88) | | -53.80 | | | | -6.27  (-7.67–-4.84) |  |
| Bosnia and Herzegovina | -12.80 | -1.02  (-2.23–0.21) | -6.77 | | -1.01  (-2.25–0.25) | -32.16 | -4.01  (-5.28–-2.73) | | -43.12 | | | | -4.41  (-5.71–-3.09) |  |
| Botswana | 421.01 | 8.12  (7.21–9.04) | 395.25 | | 7.31  (6.36–8.27) | 255.98 | 4.57  (3.17–5.99) | | 233.89 | | | | 4.21  (2.92–5.51) |  |
| Brazil | 42.00 | -0.33  (-1.21–0.55) | 32.50 | | -0.89  (-1.79–0.02) | -14.41 | -4.35  (-5.18–-3.52) | | -24.39 | | | | -4.39  (-5.26–-3.52) |  |
| Brunei | -18.65 | -5  (-7.03–-2.94) | -17.94 | | -4.99  (-7.02–-2.92) | -26.27 | -4.61  (-7.39–-1.74) | | -32.63 | | | | -4.54  (-7.27–-1.74) |  |
| Bulgaria | -8.55 | 2.78  (0.32–5.29) | 2.62 | | 2.97  (0.75–5.24) | -43.56 | -2.68  (-4.48–-0.84) | | -45.17 | | | | -2.69  (-4.48–-0.86) |  |
| Burkina Faso | 145.15 | 1.97  (0.51–3.46) | 148.08 | | 1.87  (0.43–3.33) | 76.54 | 0.2  (-1.12–1.54) | | 69.28 | | | | 0.17  (-1.1–1.46) |  |
| Burundi | 143.7 | 2.04  (1.37–2.7) | 134.36 | | 1.82  (1.29–2.35) | 52.44 | 0.14  (-0.74–1.03) | | 45.32 | | | | -0.34  (-1.3–0.62) |  |
| Cambodia | -56.91 | -7.38  (-7.9–-6.86) | -49.97 | | -6.93  (-7.46–-6.39) | -79.54 | -12.61  (-13.67–-11.54) | | -83.35 | | | | -12.99  (-13.96–-12.01) |  |
| Cameroon | 102.41 | -0.03  (-0.95–0.91) | 107.71 | | 0.07  (-0.87–1.02) | 34.93 | -1.88  (-2.96–-0.79) | | 27.53 | | | | -2.11  (-3.18–-1.03) |  |
| Canada | 1.91 | -1.29  (-1.48–-1.1) | 12.11 | | -0.87  (-1.04–-0.69) | -27.51 | -4.96  (-5.58–-4.33) | | -30.82 | | | | -4.51  (-5.11–-3.9) |  |
| Cape Verde | 29.28 | -0.95  (-1.38–-0.51) | 41.21 | | -0.72  (-1.25–-0.2) | -3.97 | -3.67  (-4.31–-3.02) | | -15.82 | | | | -4.13  (-4.75–-3.51) |  |
| Central African  Republic | 30.96 | -0.79  (-1.15–-0.44) | 31.93 | | -0.75  (-1.07–-0.44) | 15.95 | -1.63  (-2.11–-1.15) | | 10.85 | | | | -1.62  (-2.09–-1.14) |  |
| Chad | 81.61 | -0.26  (-0.97–0.46) | 88.64 | | 0.07  (-0.67–0.82) | 21.89 | -2.08  (-2.86–-1.29) | | 22.98 | | | | -2.24  (-3.01–-1.47) |  |
| Chile | 10.91 | -1.47  (-2.08–-0.86) | 14.53 | | -1.33  (-1.88–-0.79) | -15.87 | -5.42  (-6.16–-4.67) | | -26.16 | | | | -5.42  (-6.05–-4.79) |  |
| China | -79.59 | -11.26  (-11.94–-10.57) | -59.34 | | -6.82  (-6.99–-6.66) | -84.23 | -13.93  (-14.57–-13.3) | | -85.48 | | | | -13.38  (-13.92–-12.83) |  |
| Colombia | 109.74 | 1.3  (0.86–1.75) | 108.09 | | 0.98  (0.49–1.48) | 25.38 | -3.45  (-4.13–-2.77) | | 4.93 | | | | -3.39  (-4.04–-2.73) |  |
| Comoros | 363.84 | 7.36  (5.73–9.02) | 246.09 | | 5.39  (4.2–6.59) | 190.35 | 4.12  (2.88–5.36) | | 145.01 | | | | 3.56  (2.33–4.8) |  |
| Congo | 40.53 | -1.16  (-1.81–-0.5) | 40.06 | | -1.23  (-1.81–-0.65) | -4.80 | -3.04  (-3.73–-2.35) | | -12.90 | | | | -3.38  (-4.07–-2.68) |  |
| Costa Rica | -40.44 | -5.31  (-6.07–-4.54) | -27.52 | | -4.39  (-5.13–-3.65) | -48.00 | -8.17  (-9.62–-6.69) | | -50.94 | | | | -7.84  (-9.24–-6.42) |  |
| Cote d'Ivoire | -8.38 | -2.8  (-3.22–-2.39) | -3.24 | | -2.6  (-2.96–-2.23) | -27.93 | -4.37  (-4.68–-4.06) | | -32.32 | | | | -4.48  (-4.83–-4.13) |  |
| Croatia | -60.52 | -6.01  (-7.62–-4.37) | -56.35 | | -5.37  (-6.75–-3.97) | -79.27 | -12.35  (-14–-10.67) | | -82.49 | | | | -12.42  (-13.95–-10.85) |  |
| Cuba | -17.61 | -1.45  (-2.91–0.03) | -3.23 | | -0.87  (-2.13–0.4) | -20.40 | -3  (-4.84–-1.14) | | -25.38 | | | | -2.73  (-4.55–-0.88) |  |
| Cyprus | -21.16 | -2.62  (-5.26–0.09) | -19.09 | | -2.6  (-5.3–0.17) | -56.46 | -8.98  (-10.93–-6.98) | | -59.05 | | | | -8.57  (-10.56–-6.54) |  |
| Czech Republic | 1.77 | -0.25  (-0.94–0.45) | 23.62 | | 0.26  (-0.45–0.97) | -22.95 | -4.36  (-5.4–-3.31) | | -29.74 | | | | -4.08  (-5.05–-3.11) |  |
| Democratic Republic  of Congo | 109.34 | 1.15  (0.94–1.37) | 107.54 | | 1.06  (0.82–1.31) | 48.13 | -0.21  (-0.38–-0.05) | | 35.92 | | | | -0.44  (-0.63–-0.26) |  |
| Denmark | 117.29 | 3.7  (3.26–4.14) | 135.46 | | 4  (3.38–4.62) | 53.03 | 0.41  (-0.47–1.31) | | 45.45 | | | | 0.45  (-0.43–1.34) |  |
| Djibouti | 218.37 | 3.01  (2.76–3.26) | 231.26 | | 2.86  (2.63–3.1) | 158.83 | 1.68  (1.16–2.19) | | 111.35 | | | | 0.89  (0.42–1.36) |  |
| Dominica | -64.51 | -7.66  (-9.72–-5.54) | -67.07 | | -8.4  (-10.73–-6) | -72.98 | -8.31  (-10.86–-5.68) | | -73.84 | | | | -7.93  (-10.49–-5.29) |  |
| Dominican Republic | -66.05 | -9.79  (-12.55–-6.95) | -63.17 | | -9.49  (-12.21–-6.68) | -71.33 | -9.77  (-12.58–-6.87) | | -75.59 | | | | -10.16  (-12.95–-7.28) |  |
| Ecuador | -38.85 | -6.05  (-6.63–-5.45) | -36.49 | | -6.05  (-6.78–-5.31) | -63.28 | -9.92  (-10.78–-9.05) | | -69.66 | | | | -10.43  (-11.31–-9.54) |  |
| Egypt | 24.66 | -0.7  (-1.16–-0.24) | 23.31 | | -0.76  (-1.27–-0.25) | -15.97 | -4.16  (-4.8–-3.51) | | -20.29 | | | | -4.22  (-4.87–-3.57) |  |
| El Salvador | -54.83 | -6.6  (-7.99–-5.19) | -47.80 | | -5.89  (-7.12–-4.64) | -72.67 | -9.67  (-10.85–-8.47) | | -77.28 | | | | -10.03  (-11.3–-8.74) |  |
| Equatorial Guinea | 147.55 | 0.6  (-0.34–1.54) | 128.43 | | 0.27  (-0.58–1.13) | -15.89 | -3.96  (-5.03–-2.88) | | -23.64 | | | | -4.28  (-5.4–-3.14) |  |
| Eritrea | 179.08 | 2.69  (2.4–2.98) | 178.70 | | 2.53  (2.2–2.85) | 107.16 | 1.57  (1.26–1.88) | | 87.90 | | | | 0.96  (0.64–1.28) |  |
| Estonia | -63.66 | -3.8  (-4.74–-2.86) | -53.65 | | -3.33  (-4.01–-2.65) | -81.54 | -10.51  (-10.89–-10.13) | | -83.93 | | | | -10.84  (-11.22–-10.46) |  |
| Ethiopia | -5.83 | -4.01  (-4.77–-3.24) | 40.92 | | -1.14  (-1.94–-0.33) | -41.84 | -5.64  (-6.43–-4.84) | | -46.05 | | | | -5.84  (-6.6–-5.08) |  |
| Federated States  of Micronesia | -48.81 | -5.04  (-6.02–-4.06) | -29.69 | | -3.66  (-4.45–-2.87) | -57.30 | –5.71  (–7.01––4.39) | | -59.15 | | | | -5.77  (-7.04–-4.48) |  |
| Fiji | 96.99 | 3.97  (2.48–5.47) | 114.99 | | 4.1  (2.64–5.58) | 64.17 | 2.28  (0.18–4.42) | | 45.18 | | | | 2.27  (0.23–4.35) |  |
| Finland | 284.78 | 8.68  (8.03–9.33) | 313.79 | | 8.95  (8.38–9.53) | 138.15 | 2.69  (2.35–3.03) | | 106.15 | | | | 2.6  (2.3–2.9) |  |
| France | 11.85 | -0.4  (-1.08–0.28) | -4.66 | | -1.67  (-2.49–-0.85) | -27.56 | -5.76  (-6.63–-4.88) | | -39.40 | | | | -5.77  (-6.66–-4.88) |  |
| Gabon | 88.22 | 1.4  (1.15–1.65) | 66.80 | | 0.65  (0.42–0.88) | 5.62 | -2.06  (-2.26–-1.86) | | 3.82 | | | | -2.15  (-2.38–-1.92) |  |
| Georgia | 45.76 | 2.35  (0.86–3.86) | 59.94 | | 3.19  (2.27–4.11) | -21.78 | -1.1  (-1.69–-0.5) | | -28.78 | | | | -1.63  (-2.26–-1) |  |
| Germany | 124.08 | 2.66  (0.56–4.81) | 116.00 | | 2.34  (0.55–4.16) | 23.50 | -1.58  (-3.28–0.15) | | 13.82 | | | | -1.46  (-3.13–0.24) |  |
| Ghana | 76.55 | 0.25  (-0.42–0.93) | 67.56 | | -0.02  (-0.73–0.7) | 25.31 | -2.07  (-2.83–-1.3) | | 19.18 | | | | -2.12  (-2.84–-1.38) |  |
| Greece | 68.26 | 3.98  (1.22–6.82) | 77.17 | | 4.61  (1.98–7.29) | 162.35 | 3.42  (0.45–7.44) | | 102.27 | | | | 3.22  (-0.36–6.94) |  |
| Greenland | 31.06 | 1.21  (0.86–1.56) | 29.34 | | 0.87  (0.6–1.15) | -6.73 | -2.84  (-3.34–-2.33) | | -22.09 | | | | -3.21  (-3.72–-2.69) |  |
| Grenada | -25.46 | -4.94  (-7.7–-2.11) | -22.75 | | -5.03  (-7.87–-2.1) | -35.98 | -6.38  (-9.13–-3.54) | | -49.47 | | | | -6.51  (-9.25–-3.68) |  |
| Guam | -35.05 | -2.74  (-5.25–-0.16) | -25.74 | | -2.53  (-5.07–0.08) | -41.63 | -5.81  (-8.16–-3.39) | | -44.32 | | | | -5.09  (-7.47–-2.66) |  |
| Guatemala | -18.09 | -5  (-5.71–-4.29) | -23.68 | | -5.31  (-6.23–-4.38) | -63.97 | -9.78  (-10.86–-8.68) | | -68.60 | | | | -10.17  (-11.22–-9.11) |  |
| Guinea | 47.71 | 0.25  (-0.62–1.13) | 57.02 | | 0.55  (-0.36–1.47) | 9.95 | -0.63  (-1.54–0.3) | | 3.20 | | | | -1.01  (-1.91–-0.1) |  |
| Guinea-Bissau | 46.15 | -1.03  (-2.32–0.27) | 55.45 | | -0.59  (-1.95–0.78) | -1.69 | -3.2  (-4.65–-1.72) | | -8.46 | | | | -3.41  (-4.84–-1.95) |  |
| Guyana | -15.63 | -2.94  (-4.7–-1.16) | -30.37 | | -4.39  (-6.41–-2.32) | -59.80 | -5.71  (-7.79–-3.58) | | -63.72 | | | | -5.62  (-7.62–-3.57) |  |
| Haiti | -23.03 | -5.39  (-7.18–-3.56) | -20.48 | | -5.12  (-6.88–-3.32) | -59.45 | -7.58  (-9.18–-5.96) | | -64.59 | | | | -8.06  (-9.65–-6.45) |  |
| Honduras | 30.41 | -1.92  (-2.36–-1.47) | 42.25 | | -1.41  (-1.81–-1) | 4.80 | -3.58  (-3.93–-3.22) | | -13.87 | | | | -4.24  (-4.64–-3.85) |  |
| Hungary | -67.35 | -4.06  (-6.37–-1.69) | -57.08 | | -3.24  (-5.47–-0.96) | -82.79 | -12.28  (-14.25–-10.26) | | -83.43 | | | | -11.6  (-13.53–-9.64) |  |
| Iceland | -46.42 | -3.59  (-6.95–-0.12) | -48.06 | | -4.11  (-7.24–-0.88) | -69.60 | -10.75  (-13.75–-7.64) | | -72.36 | | | | -10.53  (-13.46–-7.49) |  |
| India | 117.22 | 2.16  (1.52–2.8) | 132.99 | | 2.55  (2.1–3) | 29.18 | -1.82  (-2.21–-1.42) | | 11.79 | | | | -2.01  (-2.41–-1.6) |  |
| Indonesia | -77.57 | -9.85  (-10.4–-9.3) | -74.37 | | -9.27  (-9.89–-8.64) | -80.68 | -12.35  (-13.38–-11.3) | | -83.02 | | | | -12.54  (-13.55–-11.53) |  |
| Iran | -6.75 | -2.21  (-2.59–-1.82) | -5.79 | | -2.51  (-2.91–-2.1) | -18.05 | -4.72  (-5.51–-3.93) | | -39.08 | | | | -5.45  (-6.13–-4.76) |  |
| Iraq | -2.20 | -4.85  (-5.75–-3.93) | -5.43 | | -5.1  (-6–-4.19) | -49.33 | -9.34  (-10.27–-8.39) | | -55.68 | | | | -9.76  (-10.7–-8.81) |  |
| Ireland | 38.23 | -0.08  (-1.75–1.61) | 46.42 | | 0.21  (-1.3–1.74) | -12.83 | -3.08  (-4.9–-1.23) | | -17.96 | | | | -3.23  (-5.04–-1.38) |  |
| Israel | -24.42 | -3.96  (-5.32–-2.59) | -28.57 | | -4.64  (-6.25–-3.01) | -68.09 | -10.25  (-11.81–-8.66) | | -70.57 | | | | -10.12  (-11.73–-8.48) |  |
| Italy | -50.35 | -6.08  (-7.4–-4.75) | -53.12 | | -6.34  (-7.43–-5.23) | -66.00 | -9.94  (-11.11–-8.76) | | -70.94 | | | | -9.86  (-11–-8.71) |  |
| Jamaica | 27.55 | 0.45  (-1.13–2.06) | 30.93 | | 0.42  (-1.24–2.11) | -16.03 | -0.97  (-3.74–1.87) | | -13.35 | | | | -0.23  (-3.04–2.68) |  |
| Japan | -71.40 | -9.14  (-11.29–-6.94) | -76.32 | | -9.68  (-11.82–-7.5) | -66.92 | -10.02  (-11.59–-8.41) | | -77.34 | | | | -11.01  (-12.67–-9.32) |  |
| Jordan | 49.94 | -3.23  (-4.36–-2.09) | 48.50 | | -3.44  (-4.58–-2.28) | -12.98 | -7.65  (-9–-6.29) | | -27.13 | | | | -8.38  (-9.69–-7.05) |  |
| Kazakhstan | -25.30 | -2.61  (-5.65–0.52) | -23.59 | | -2.43  (-4.85–0.05) | -79.22 | -11.71  (-13.77–-9.6) | | -79.96 | | | | -11.91  (-14.03–-9.74) |  |
| Kenya | 44.50 | -1.56  (-2.39–-0.72) | 40.49 | | -2.1  (-2.82–-1.38) | 21.96 | -3.06  (-4.08–-2.03) | | 9.48 | | | | -3.42  (-4.44–-2.39) |  |
| Kiribati | 13.61 | -2.87  (-3.99–-1.73) | 11.68 | | -2.94  (-3.96–-1.91) | -22.92 | -4.28  (-5.54–-3.01) | | -25.56 | | | | -4.4  (-5.63–-3.15) |  |
| Kuwait | 27.30 | -4.25  (-4.58–-3.92) | 17.72 | | -4.76  (-5.25–-4.27) | -42.73 | -10.11  (-11.6–-8.59) | | -48.95 | | | | -10.53  (-12.01–-9.02) |  |
| Kyrgyzstan | 296.75 | 6.43  (4.77–8.11) | 220.39 | | 5.39  (3.6–7.21) | -1.67 | -2.6  (-4.31–-0.86) | | -8.38 | | | | -3.11  (-4.78–-1.41) |  |
| Laos | -75.56 | -10.59  (-11.02–-10.16) | -71.93 | | -9.79  (-10.25–-9.33) | -86.33 | -14.38  (-15.4–-13.34) | | -87.61 | | | | -14.63  (-15.65–-13.59) |  |
| Latvia | -71.12 | -5.9  (-6.3–-5.51) | -65.75 | | -5.47  (-5.99–-4.95) | -82.19 | -10.62  (-11.16–-10.08) | | -83.62 | | | | -10.84  (-11.42–-10.26) |  |
| Lebanon | 30.06 | -2.31  (-3.11–-1.49) | 27.06 | | -2.47  (-3.24–-1.7) | -28.62 | -6.41  (-7.34–-5.47) | | -33.82 | | | | -6.51  (-7.47–-5.55) |  |
| Lesotho | 127.62 | 3.53  (2.33–4.75) | 126.73 | | 2.87  (1.61–4.14) | 104.87 | 2.58  (0.59–4.61) | | 110.27 | | | | 2.73  (0.7–4.81) |  |
| Liberia | 19.39 | -1.58  (-1.95–-1.2) | 25.14 | | -1.38  (-1.88–-0.88) | -26.18 | -3.53  (-3.9–-3.17) | | -31.69 | | | | -3.89  (-4.2–-3.58) |  |
| Libya | -12.14 | -4.42  (-5.46–-3.36) | -10.51 | | -4.16  (-5.12–-3.2) | -25.41 | -6.3  (-7.71–-4.87) | | -28.47 | | | | -6.37  (-7.76–-4.96) |  |
| Lithuania | -27.33 | -1.35  (-2.2–-0.49) | -16.42 | | -0.72  (-1.48–0.04) | -47.70 | -4.54  (-5.54–-3.53) | | -51.54 | | | | -4.59  (-5.69–-3.47) |  |
| Luxembourg | 340.81 | 8.18  (5.55–10.87) | 368.07 | | 8.51  (5.88–11.2) | 114.25 | 2.29  (0.2–4.43) | | 99.80 | | | | 2.36  (0.29–4.46) |  |
| Macedonia | -18.19 | -1.97  (-2.95–-0.98) | -16.06 | | -2.04  (-2.87–-1.21) | -61.13 | -8.72  (-9.69–-7.73) | | -62.99 | | | | -8.33  (-9.23–-7.43) |  |
| Madagascar | 142.89 | 2.68  (0.89–4.51) | 138.15 | | 2.42  (0.68–4.19) | 82.31 | 2.49  (0.29–4.74) | | 72.34 | | | | 2.35  (0.12–4.63) |  |
| Malawi | 69.92 | -2.05  (-3.79–-0.27) | 68.70 | | -2.13  (-3.81–-0.42) | 6.29 | -3.75  (-5.44–-2.03) | | -5.68 | | | | -4.01  (-5.7–-2.28) |  |
| Malaysia | -41.80 | -6.18  (-6.68–-5.68) | -40.02 | | -6.28  (-6.76–-5.79) | -64.7 | -10.93  (-11.59–-10.26) | | -65.09 | | | | -10.05  (-10.63–-9.47) |  |
| Maldives | -64.03 | -10.77  (-11.19–-10.35) | -58.49 | | -10.58  (-10.96–-10.2) | -88.84 | -18.02  (-18.98–-17.05) | | -88.22 | | | | -17.5  (-18.33–-16.66) |  |
| Mali | 93.71 | -0.25  (-0.82–0.33) | 90.28 | | -0.24  (-0.86–0.38) | 19.25 | -2.64  (-3.05–-2.23) | | 14.18 | | | | -2.88  (-3.29–-2.47) |  |
| Malta | -11.42 | -1.6  (-4.65–1.55) | -14.83 | | -2  (-5.01–1.11) | -45.68 | -7.93  (-11.22–-4.52) | | -49.68 | | | | -6.99  (-10.21–-3.66) |  |
| Marshall Islands | -5.28 | 0.86  (-1.28–3.06) | 2.64 | | 0.68  (-1.47–2.87) | -37.05 | -4.19  (-6.52–-1.81) | | -41.72 | | | | -4.04  (-6.36–-1.66) |  |
| Mauritania | 2.09 | -2.44  (-2.98–-1.89) | 12.71 | | -1.96  (-2.65–-1.27) | -30.28 | -5.07  (-5.7–-4.45) | | -38.02 | | | | -5.49  (-6.09–-4.89) |  |
| Mauritius | -53.34 | -3.64  (-4.97–-2.29) | -45.22 | | -3.46  (-4.7–-2.2) | -36.07 | -5.4  (-6.89–-3.9) | | -42.57 | | | | -4.91  (-6.29–-3.5) |  |
| Mexico | 32.51 | -1.85  (-2.54–-1.17) | 19.26 | | -2.2  (-2.93–-1.46) | -27.50 | -5.79  (-6.45–-5.12) | | -30.72 | | | | -5.33  (-5.96–-4.69) |  |
| Moldova | 133.97 | 4.45  (2.7–6.23) | 103.48 | | 3.89  (2.55–5.25) | -28.01 | -2.98  (-4.87–-1.05) | | -31.98 | | | | -3.13  (-4.97–-1.26) |  |
| Mongolia | 475.55 | 8.55  (7.76–9.36) | 481.25 | | 8.56  (7.89–9.25) | 135.39 | 1.89  (1.41–2.37) | | 116.05 | | | | 1.76  (1.23–2.29) |  |
| Montenegro | 5.74 | -2.51  (-4.4–-0.57) | 13.58 | | -2.19  (-4.02–-0.33) | -34.08 | -5.19  (-6.78–-3.56) | | -42.31 | | | | -5.64  (-7.23–-4.03) |  |
| Morocco | -26.61 | -4.09  (-4.91–-3.27) | -27.07 | | -4.12  (-4.93–-3.31) | -41.94 | -7.08  (-7.94–-6.22) | | -51.67 | | | | -7.45  (-8.35–-6.55) |  |
| Mozambique | 142.98 | 0.99  (0.88–1.1) | 133.15 | | 0.63  (0.45–0.81) | 40.77 | -0.26  (-0.55–0.04) | | 30.69 | | | | -0.43  (-0.67–-0.2) |  |
| Myanmar | 30.47 | 0.31  (-0.1–0.71) | 46.28 | | 0.86  (0.58–1.14) | -52.66 | -6.14  (-6.53–-5.75) | | -58.50 | | | | -6.44  (-6.81–-6.06) |  |
| Namibia | 232.50 | 4.23  (2.68–5.82) | 225.86 | | 3.76  (2.23–5.31) | 57.73 | -1.42  (-3.11–0.31) | | 54.34 | | | | -1.44  (-3.14–0.29) |  |
| Nepal | 29.16 | -1.22  (-1.7–-0.73) | 42.25 | | -0.95  (-1.39–-0.5) | -21.29 | -4.41  (-4.88–-3.94) | | -34.39 | | | | -4.85  (-5.34–-4.36) |  |
| Netherlands | 126.21 | 6.79  (5.01–8.6) | 133.48 | | 6.64  (5.05–8.25) | 47.76 | 1.31  (0.07–2.58) | | 36.65 | | | | 1.47  (0.23–2.72) |  |
| New Zealand | 153.83 | 4.16  (3.26–5.06) | 130.78 | | 3.51  (2.57–4.45) | 54.63 | 0.8  (-0.39–2.01) | | 44.57 | | | | 0.8  (-0.32–1.92) |  |
| Nicaragua | -21.05 | -4.24  (-5.03–-3.44) | -15.32 | | -3.83  (-4.67–-2.99) | -59.00 | -8.23  (-9.05–-7.39) | | -65.00 | | | | -8.42  (-9.23–-7.61) |  |
| Niger | 60.69 | -1.33  (-2.02–-0.64) | 64.50 | | -1.06  (-1.76–-0.35) | 4.73 | -3.46  (-4.07–-2.85) | | -8.07 | | | | -3.95  (-4.54–-3.35) |  |
| Nigeria | 67.59 | -0.29  (-0.83–0.26) | 63.36 | | -0.3  (-0.9–0.3) | -2.37 | -3.73  (-4.27–-3.18) | | -3.04 | | | | -4  (-4.53–-3.46) |  |
| North Korea | 21.06 | -0.62  (-1.06–-0.17) | 37.05 | | -0.4  (-0.86–0.05) | 8.85 | -2.66  (-3.24–-2.08) | | -2.70 | | | | -2.53  (-3.12–-1.94) |  |
| Northern Mariana Islands | -65.79 | -10.44  (-14.06–-6.67) | -54.64 | | -9.4  (-12.92–-5.74) | -50.40 | -9.17  (-12.19–-6.04) | | -62.75 | | | | -9.27  (-12.32–-6.11) |  |
| Norway | 18.18 | 1.3  (0.67–1.94) | 4.46 | | 0.04  (-0.3–0.37) | -43.85 | -4.48  (-4.87–-4.09) | | -46.62 | | | | -4.6  (-4.98–-4.22) |  |
| Oman | -19.82 | -6.04  (-7.6–-4.45) | -16.16 | | -5.87  (-7.4–-4.31) | -53.67 | -7.91  (-8.95–-6.85) | | -51.70 | | | | -8.11  (-9.17–-7.04) |  |
| Pakistan | 121.74 | 0.98  (0.34–1.62) | 151.44 | | 1.79  (1.14–2.45) | 34.37 | -1.81  (-2.5–-1.12) | | 27.16 | | | | -2.08  (-2.76–-1.39) |  |
| Palestine | 8.02 | -3  (-3.49–-2.5) | 9.35 | | -3  (-3.46–-2.54) | -9.39 | -4.43  (-4.96–-3.89) | | -21.63 | | | | -4.81  (-5.36–-4.26) |  |
| Panama | -11.37 | -4.71  (-5.84–-3.57) | -8.28 | | -4.35  (-5.31–-3.38) | -32.91 | -6.43  (-7.13–-5.73) | | -37.79 | | | | -6.12  (-6.81–-5.42) |  |
| Papua New Guinea | 808.43 | 9.83  (8.23–11.45) | 743.80 | | 9.28  (7.81–10.78) | 422.65 | 8.55  (6.51–10.62) | | 417.40 | | | | 8.42  (6.46–10.43) |  |
| Paraguay | -15.97 | -3.92  (-5.44–-2.38) | -4.36 | | -3.32  (-4.63–-1.99) | -27.59 | -4.56  (-5.69–-3.43) | | -35.72 | | | | -5.08  (-6.21–-3.94) |  |
| Peru | -19.36 | -3.78  (-4.28–-3.28) | -12.15 | | -3.39  (-3.91–-2.86) | -46.16 | -7.39  (-8.25–-6.53) | | -57.49 | | | | -8.04  (-8.89–-7.17) |  |
| Philippines | 100.67 | 1.99  (1.27–2.71) | 79.38 | | 1.13  (0.75–1.52) | -4.41 | -3.02  (-3.5–-2.54) | | -4.35 | | | | -2.55  (-3.02–-2.09) |  |
| Poland | -67.94 | -8.02  (-8.79–-7.25) | -65.52 | | -7.78  (-8.53–-7.02) | -80.54 | -11.59  (-12.33–-10.84) | | -81.24 | | | | -11.26  (-11.94–-10.56) |  |
| Portugal | -69.60 | -6.63  (-7.17–-6.1) | -71.11 | | -7.17  (-7.91–-6.42) | -72.12 | -9.38  (-10.06–-8.69) | | -78.68 | | | | -10.17  (-10.82–-9.51) |  |
| Puerto Rico | -45.90 | -3.82  (-4.63–-3) | -42.84 | | -4.25  (-4.98–-3.51) | -61.95 | -7.38  (-8.53–-6.22) | | -66.17 | | | | -7.17  (-8.34–-5.99) |  |
| Qatar | 620.24 | 1.94  (1.71–2.16) | 640.78 | | 2.11  (1.9–2.32) | 121.3 | -2.6  (-4.16–-1.02) | | 145.93 | | | | -2.56  (-3.97–-1.13) |  |
| Romania | -16.44 | -1.4  (-2.53–-0.26) | -20.61 | | -1.92  (-2.9–-0.93) | -49.15 | -5.91  (-6.96–-4.86) | | -55.40 | | | | -6.35  (-7.37–-5.31) |  |
| Russian Federation | 10.66 | 0.88  (-0.58–2.35) | -7.24 | | -0.74  (-1.83–0.35) | -56.50 | -6.17  (-7.4–-4.93) | | -57.57 | | | | -6.15  (-7.44–-4.85) |  |
| Rwanda | 41.86 | -1.91  (-2.38–-1.44) | 30.54 | | -2.53  (-3.01–-2.05) | -48.08 | -7.5  (-8.31–-6.67) | | -55.89 | | | | -7.97  (-8.81–-7.13) |  |
| Saint Lucia | -79.97 | -11.69  (-14.25–-9.05) | -79.13 | | -11.68  (-14.23–-9.06) | -82.45 | -13.08  (-15.77–-10.29) | | -84.54 | | | | -13.09  (-15.77–-10.33) |  |
| Saint Vincent and  the Grenadines | -70.06 | -9.85  (-12.15–-7.5) | -71.12 | | -10.03  (-12.45–-7.54) | -75.23 | -10.53  (-13.15–-7.84) | | -78.40 | | | | -10.65  (-13.24–-7.99) |  |
| Samoa | -76.24 | -10.81  (-11.91–-9.69) | -74.28 | | -10.54  (-11.64–-9.43) | -80.59 | -12.32  (-13.62–-10.99) | | -82.38 | | | | -12.62  (-13.93–-11.29) |  |
| Sao Tome and Principe | 112.54 | 0.86  (-0.2–1.92) | 114.55 | | 0.92  (-0.07–1.91) | 25.16 | -1.35  (-2.25–-0.43) | | 13.67 | | | | -1.73  (-2.6–-0.85) |  |
| Saudi Arabia | 7.99 | -3.52  (-3.95–-3.09) | 5.67 | | -3.58  (-3.93–-3.23) | -43.96 | -7.61  (-8.17–-7.05) | | -36.65 | | | | -7.58  (-8.13–-7.04) |  |
| Senegal | 13.67 | -2.63  (-3.37–-1.88) | 21.33 | | -2.35  (-3.14–-1.56) | 0.30 | -3.79  (-4.54–-3.04) | | -14.39 | | | | -4.29  (-5.07–-3.51) |  |
| Serbia | -19.64 | -0.93  (-1.94–0.08) | -14.59 | | -0.81  (-1.72–0.11) | -58.10 | -6.65  (-7.89–-5.4) | | -64.18 | | | | -7.06  (-8.27–-5.84) |  |
| Seychelles | -70.43 | -9.16  (-9.61–-8.7) | -66.97 | | -8.9  (-9.36–-8.43) | -81.70 | -12.44  (-12.92–-11.96) | | -80.73 | | | | -12.24  (-12.75–-11.73) |  |
| Sierra Leone | 52.65 | -0.9  (-1.42–-0.38) | 65.48 | | -0.45  (-1.11–0.22) | 3.14 | -2.56  (-3.28–-1.84) | | -0.53 | | | | -2.89  (-3.58–-2.2) |  |
| Singapore | 243.61 | 5.24  (4.45–6.04) | 209.73 | | 4.71  (3.73–5.7) | 744.36 | 12.67  (10.6–14.78) | | 380.15 | | | | 9.28  (7.78–10.79) |  |
| Slovakia | -47.15 | -5.33  (-6.59–-4.04) | -45.03 | | -5.28  (-6.42–-4.13) | -71.61 | -10.26  (-11.88–-8.61) | | -72.94 | | | | -10.09  (-11.75–-8.4) |  |
| Slovenia | -91.00 | -14.11  (-15.36–-12.83) | -89.01 | | -13.69  (-15.04–-12.31) | -92.79 | -18.96  (-20.82–-17.06) | | -94.36 | | | | -19.35  (-21.1–-17.55) |  |
| Solomon Islands | -21.81 | -4.81  (-5.72–-3.89) | -12.69 | | -4.46  (-5.38–-3.53) | -36.36 | -5.94  (-7.01–-4.86) | | -39.88 | | | | -6.09  (-7.16–-5.01) |  |
| Somalia | 155.64 | 3.4  (2.6–4.2) | 161.76 | | 3.25  (2.47–4.03) | 117.67 | 2.37  (1.45–3.3) | | 90.41 | | | | 1.81  (0.93–2.7) |  |
| South Africa | -25.06 | -2.95  (-4.59–-1.28) | -10.34 | | -2.93  (-4.27–-1.58) | -43.09 | -7.43  (-9.34–-5.48) | | -51.01 | | | | -8.47  (-10.6–-6.28) |  |
| South Korea | -41.20 | -7.87  (-9.72–-5.99) | -58.37 | | -9.42  (-10.81–-8.01) | -70.96 | -13.5  (-14.88–-12.11) | | -79.23 | | | | -14.02  (-15.33–-12.69) |  |
| South Sudan | 125.78 | 3.09  (3.02–3.15) | 110.99 | | 2.43  (2.37–2.5) | 112.37 | 3.04  (2.85–3.23) | | 108.02 | | | | 3.06  (2.85–3.26) |  |
| Spain | -46.04 | -4.76  (-5.25–-4.26) | -46.96 | | -5.15  (-5.47–-4.82) | -60.74 | -8.13  (-8.54–-7.72) | | -67.04 | | | | -8.49  (-8.89–-8.09) |  |
| Sri Lanka | -34.94 | -2.79  (-3.92–-1.64) | -20.41 | | -2.04  (-3.21–-0.86) | -69.12 | -8.22  (-9.28–-7.14) | | -70.42 | | | | -7.95  (-9.03–-6.87) |  |
| Sudan | -25.56 | -4.6  (-5.53–-3.65) | -23.46 | | -4.52  (-5.49–-3.54) | -62.51 | -7.58  (-8.45–-6.71) | | -67.15 | | | | -8.18  (-9.01–-7.34) |  |
| Suriname | -34.52 | -5.82  (-7.52–-4.09) | -35.88 | | -6.13  (-7.88–-4.34) | -59.45 | -8.83  (-10.62–-6.99) | | -64.29 | | | | -9  (-10.71–-7.26) |  |
| Swaziland | 330.37 | 7.41  (4.71–10.17) | 311.14 | | 6.38  (3.62–9.22) | 175.43 | 2.75  (-0.85–6.48) | | 167.01 | | | | 2.78  (-0.88–6.57) |  |
| Sweden | 192.11 | 7.33  (5.97–8.71) | 170.23 | | 6.31  (5.1–7.53) | 1.37 | -1.36  (-2.55–-0.15) | | -2.63 | | | | -1.03  (-2.11–0.06) |  |
| Switzerland | 150.85 | 4.53  (3.27–5.82) | 149.38 | | 4.15  (2.85–5.47) | 9.33 | -1.76  (-3.24–-0.27) | | 4.10 | | | | -1.53  (-2.98–-0.06) |  |
| Syria | -56.87 | -6.75  (-7.62–-5.88) | -54.62 | | -6.48  (-7.32–-5.63) | -71.83 | -10.61  (-11.81–-9.39) | | -77.41 | | | | -10.73  (-11.91–-9.52) |  |
| Taiwan  (Province of China) | -59.78 | -8.27  (-9.28–-7.26) | -57.79 | | -7.87  (-9.04–-6.69) | -77.91 | -13.32  (-14.71–-11.91) | | -80.47 | | | | -12.86  (-14.27–-11.43) |  |
| Tajikistan | 274.95 | 3.28  (1.4–5.2) | 298.44 | | 4.17  (2.51–5.85) | 125.42 | 1  (-0.55–2.56) | | 112.09 | | | | 0.72  (-0.81–2.27) |  |
| Tanzania | 158.57 | 2.69  (1.49–3.92) | 150.98 | | 2.16  (0.95–3.39) | 89.77 | 1.79  (0.6–2.99) | | 69.53 | | | | 1.39  (0.17–2.64) |  |
| Thailand | 59.36 | 1.9  (1.21–2.61) | 71.69 | | 1.73  (0.96–2.51) | -3.45 | -3.17  (-4.14–-2.2) | | -28.96 | | | | -3.85  (-4.62–-3.07) |  |
| The Bahamas | 171.82 | 3.5  (2.59–4.42) | 174.36 | | 3.52  (2.64–4.4) | 113.64 | 3.39  (1.83–4.97) | | 94.37 | | | | 3.36  (1.8–4.95) |  |
| The Gambia | 62.30 | -0.41  (-1.23–0.43) | 75.22 | | -0.05  (-0.91–0.82) | 53.46 | -1.05  (-1.93–-0.16) | | 37.98 | | | | -1.22  (-2.09–-0.34) |  |
| Timor-Lester | -54.65 | -8.81  (-10.02–-7.57) | -54.46 | | -8.44  (-9.4–-7.48) | -76.44 | -12.69  (-13.53–-11.85) | | -82.23 | | | | -13.05  (-13.9–-12.19) |  |
| Togo | 72.96 | 0.26  (-0.53–1.06) | 83.24 | | 0.42  (-0.43–1.28) | 31.39 | -1.81  (-2.69–-0.92) | | 14.88 | | | | -2.05  (-2.91–-1.18) |  |
| Tonga | -40.70 | -4.70  (-5.74–-3.64) | -30.73 | | -4.07  (-5.03–-3.09) | -55.14 | -6.21  (-7.6–-4.79) | | -55.32 | | | | -5.96  (-7.31–-4.59) |  |
| Trinidad and Tobago | -53.29 | -7.4  (-9.12–-5.64) | -53.73 | | -7.88  (-9.82–-5.89) | -67.29 | -9.89  (-11.96–-7.76) | | -68.67 | | | | -9.68  (-11.69–-7.64) |  |
| Tunisia | -38.69 | -4.89  (-5.94–-3.83) | -38.02 | | -4.82  (-5.85–-3.79) | -48.92 | -7.77  (-8.7–-6.82) | | -57.61 | | | | -8.11  (-9.02–-7.2) |  |
| Turkey | -36.51 | -4.90  (-5.67–-4.12) | -36.31 | | -4.90  (-5.72–-4.07) | -71.57 | -9.27  (-10.42–-8.11) | | -80.42 | | | | -10.79  (-12.26–-9.29) |  |
| Turkmenistan | 134.62 | 3.49  (3.4–3.59) | 148.48 | | 3.44  (2.77–4.12) | 25.64 | -0.99  (-1.8–-0.17) | | 18.49 | | | | -0.9  (-1.63–-0.17) |  |
| Uganda | 111.25 | 0.57  (-0.13–1.28) | 115.36 | | 0.3  (-0.27–0.86) | 39.72 | -1.23  (-1.79–-0.66) | | 27.55 | | | | -1.67  (-2.29–-1.05) |  |
| Ukraine | 71.89 | 3.41  (3.08–3.74) | 49.11 | | 2.44  (2.1–2.78) | -18.99 | -1.48  (-2.31–-0.64) | | -18.96 | | | | -1.47  (-2.37–-0.56) |  |
| United Arab Emirates | 105.06 | -3.46  (-4.44–-2.46) | 105.95 | | -3.44  (-4.38–-2.49) | 98.06 | -4.43  (-5.52–-3.32) | | 96.34 | | | | -4.18  (-5.23–-3.12) |  |
| United Kingdom | 33.98 | 2.46  (1.21–3.72) | 31.18 | | 2.22  (1.08–3.37) | -13.06 | -1.63  (-2.05–-1.2) | | -17.86 | | | | -1.59  (-2.08–-1.1) |  |
| United States | -45.43 | -3.21  (-3.64–-2.77) | 1.89 | | -0.49  (-1.13–0.15) | -33.88 | -4.04  (-4.84–-3.24) | | -33.44 | | | | -3.81  (-4.56–-3.06) |  |
| Uruguay | -11.16 | -0.64  (-2.31–1.06) | -22.37 | | -1.83  (-3.35–-0.29) | -50.51 | -4.52  (-6.12–-2.89) | | -51.25 | | | | -4.22  (-5.78–-2.64) |  |
| Uzbekistan | 287.25 | 4.48  (1.73–7.3) | 235.89 | | 4.46  (2.13–6.84) | 48.44 | -1.44  (-3.42–0.59) | | 34.39 | | | | -2  (-3.95–-0.01) |  |
| Vanuatu | 111.26 | 2.57  (0.98–4.18) | 129.67 | | 2.86  (1.3–4.44) | 82.51 | 1.66  (-0.57–3.93) | | 66.35 | | | | 1.56  (-0.64–3.81) |  |
| Venezuela | -1.05 | -3.58  (-4.3–-2.85) | -1.77 | | -3.68  (-4.45–-2.92) | -31.08 | -6.43  (-7.28–-5.58) | | -36.86 | | | | -6.41  (-7.19–-5.63) |  |
| Vietnam | 77.51 | 2.37  (1.36–3.38) | 97.32 | | 2.46  (1.44–3.5) | -3.49 | -2.61  (-3.7–-1.52) | | -9.70 | | | | -2.57  (-3.68–-1.44) |  |
| Virgin Islands, U.S. | -63.09 | -7.55  (-9.74–-5.31) | -60.32 | | -7.69  (-9.91–-5.43) | -66.24 | -8.71  (-11.55–-5.78) | | -71.62 | | | | -8.80  (-11.63–-5.88) |  |
| Yemen | -23.64 | -5.71  (-6.44–-4.98) | -21.95 | | -5.61  (-6.34–-4.87) | -52.03 | -8.52  (-9.48–-7.56) | | -61.00 | | | | -9.22  (-10.19–-8.24) |  |
| Zambia | 162.66 | 0.91  (-1.12–2.98) | 159.26 | | 0.93  (-1.05–2.96) | 41.96 | -0.76  (-2.75–1.28) | | 28.94 | | | | -1.14  (-3.17–0.94) |  |
| Zimbabwe | 216.16 | 5.34  (3.89–6.81) | 210.45 | | 4.87  (3.43–6.32) | 236.41 | 5  (2.23–7.86) | | 244.66 | | | | 4.96  (2.13–7.86) |  |

MDR-TB: multidrug-resistant tuberculosis; DALYs: disability-adjusted life-years; EAPC: estimated annual percentage change; ASR, age-standardized rate; CI, confidence interval; UI: uncertainty interval. Percentage change in absolute number was calculated based on the crew data.
